# Fluorinating the Sugar and the Nucleotide: Exploring Fluorination Within GDP-Mannose Probes Using Chemoenzymatic Synthesis

**DOI:** 10.1021/jacsau.5c00626

**Published:** 2025-07-31

**Authors:** Jonathan P. Dolan, Sean T. Evans, Caecilie M. M. Benckendorff, Suat Sari, Aisling Ní Cheallaigh, Gavin J. Miller

**Affiliations:** † School of Chemical & Physical Sciences and Centre for Glycoscience, 4212 Keele University, Keele, Staffordshire ST5 5BG, U.K.; ‡ Faculty of Pharmacy, Department of Pharmaceutical Chemistry, 37515 Hacettepe University, Ankara 06100, Turkey

**Keywords:** sugar nucleotide, fluorination, chemical probe, dehydrogenase, glycan

## Abstract

Fluorinated glycans offer a prime opportunity to study the intricacies of their associated binding events with proteins, invoke resistance toward enzymatic hydrolysis, and modulate carbohydrate physicochemical properties. Sugar nucleotides are the key building blocks used by glycosyltransferases and associated enzymes to assemble glycans and, as such, represent a considerable landscape of opportunity to develop fluorinated motifs and enable structure-to-function understanding. Herein, we target the isosteric inclusion of fluorine within the nucleoside diphosphate sugar framework of GDP-mannose using a chemoenzymatic approach. Utilizing chemical synthesis to incorporate bespoke fluorine modifications and a promiscuous pyrophosphorylase, to assemble the sugar nucleotide, enables first-in-class access to GDP-mannoses containing fluorine within the nucleotide alongside double fluorination, within both the pyranose and nucleotide. These materials are utilized to probe a guanosine diphosphate mannose dehydrogenase critical to mucoid *Pseudomonas aeruginosa* alginate biosynthesis. This work provides an exemplar framework for incorporating fluorine within the nucleotide of derived sugar nucleotides and thus the capability to study glycosyltransferesase utilizing GDP-mannose more broadly.

## Graphical abstract

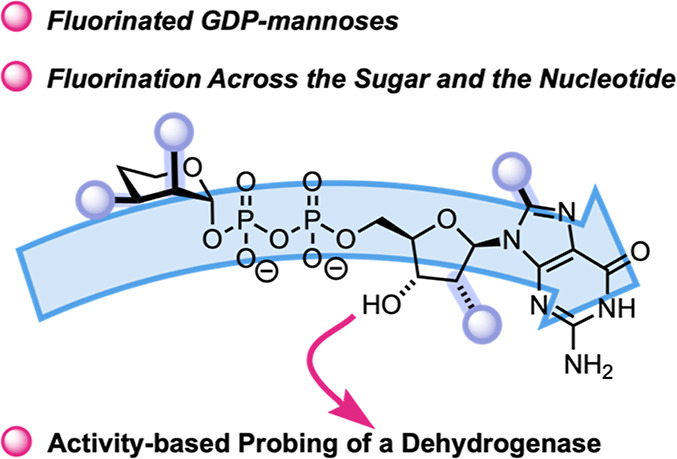

## Introduction

Fluorinated glycans have proven instrumental toward the development and understanding of carbohydrate–protein interactions.^1,2^ Indeed, such materials offer a prime opportunity to study the criticality of amino acids involved in glycan substrate binding without the need for diverse mutant libraries, alongside demonstrating improved resistance to enzymatic hydrolysis.^3–5^ Access to increasingly large libraries of such materials has also proven to enable the development of glycans for biosensing and in diagnostic applications, together with exploring polysaccharide structure-to-function properties.^6–9^

Within glycan biosynthesis, sugar nucleotides are imperative building blocks. Composed of an activated sugar donor, they are used to glycosidate a range of acceptors, releasing an energetic mono- or dinucleotide byproduct.^10,11^ The production of fluorinated sugar nucleotide donors, enzymatic glycosylation with these building blocks, and use of fluorinated acceptors are areas of intensifying importance.^1,10,12^ They have seen use as crystallization partners within structural biology studies and as probes of substrate specificity and equilibrium positions. For example, using STD-NMR polyfluorinated derivatives of UDP-Gal*p* and UDP-Gal*f* were demonstrated to be superior ligands for UDP-galactopyranose mutase, compared to a nonfluorinated parent.^13,14^ Expanding this technique to combine ^19^F NMR with STD-NMR has shown that fluorinated carbohydrates can act as versatile sensors for studying carbohydrate–lectin interactions.^15^ This has further evolved into the development of sensitive assays for these interactions, allowing for elucidation of binding dissociation energies (*K*_D_) and inhibitory activity using ^19^F T2 NMR competition assays.^16^ More recently, the assembly of 7-deoxy-7-fluorosialyl glycosides using sialyltransferases from the corresponding fluorinated CMP-Sia donors was demonstrated, with the resulting glycosides found to be 40–250-fold more resistant toward cleavage by GH33 sialidases.^4^ Despite fluorination being shown to have minimal impact on the conformation of the sugar component,^1^ the success of enzymatic sugar nucleotide synthesis and subsequent utilization of such materials relies on the ability of the involved enzymes to recognize and efficiently utilize fluorinated substrates.^10^ This is complemented by a prerequisite chemical synthesis to install fluorine atom(s) at the required position(s).

In this context, we recently disclosed access to two fluorinated GDP-mannose sugar nucleotides using a chemoenzymatic approach. Chemical synthesis of 4-deoxy-4-fluoro and 6-deoxy-6-fluoro mannose 1-phosphates enabled exploration of a guanosine diphosphate-mannopyranose pyrophosphatase from *Salmonella enterica* (*Se*GDP-Man-PP) to deliver C4- and C6-position fluorinated GDP-Mans.^17^ Considering this, we sought here to broaden the scope of fluorine incorporation across the entire sugar nucleotide construct (Figure 1), envisaging that access to such motifs may offer new capability in exploring critical ligand–protein interactions within associated glycan-utilizing enzymes.^15,16^ Furthermore, a fluorinated nucleotide motif may provide new tools toward studying problematic inhibition of glycosyltransferases by nucleoside diphosphate byproducts, alongside systems/enzymes that are commonly used to regenerate the NTP (or degrade the NDP). Ribose nucleotide fluorination may also present an alternate conformational preference and affect binding to the target enzyme.

**Figure 1:**
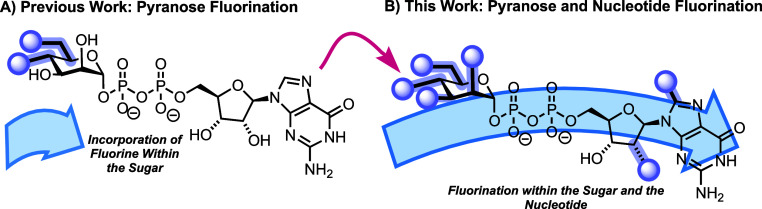
(A) Utilizing a chemoenzymatic approach to access pyranose-only fluorination with GDP-mannose; (B) work herein to expand the coverage of fluorine incorporation, targeting pyranose positions alongside ribose and guanine nucleotide components.

## Results and Discussion

### Pyranose and Nucleotide Building Block Synthesis

Envisaging a chemoenzymatic strategy whereby mannose 1-phosphates and fluorinated guanosine triphosphates (GTPs) were synthesized chemically, followed by enzymatic nucleoside diphosphate formation using *Se*GDP-Man-PP, we first sought 3-deoxy-3-fluoro and 2-deoxy-2-fluoro mannose 1-phosphates **3** and **6** (Scheme 1). The synthesis of 3-deoxy 3-fluoro derivative **3** began from known thioglycoside **1** (Scheme 1a).^18^ Protecting group manipulations to remove the 4,6-*O*-benzylidene under mildly acidic conditions, followed by benzylation of the resulting 4,6-hydroxyl groups, yielded compound **2** in 64% yield over two steps. Installation of a protected anomeric phosphate was completed next, glycosylating **2** with dibenzyl phosphate in the presence of NIS and TfOH and yielding the protected phosphate in 62% yield. Finally, global hydrogenolysis gave the target 3-deoxy-3-fluoro mannose 1-phosphate **3** in 28% yield, following purification using strong-anion exchange (SAX) chromatography.

**Scheme 1:**
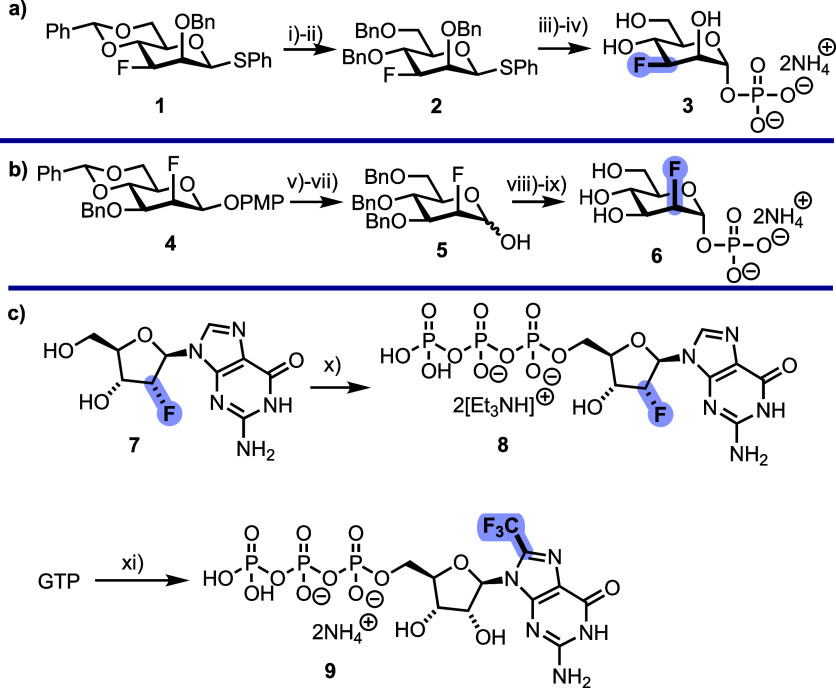
(a) Chemical Synthesis of 3-Deoxy-3-fluoro Mannose 1-Phosphate 3, Reagents and Conditions: (i) *p*-TsOH·H_2_O, MeOH, 89% (ii) BnBr, NaH, DMF, 72% (iii) Dibenzyl Phosphate, NIS, TfOH, CH_2_Cl_2_, 62% (iv) H_2_, Pd­(OH)_2_/C, Pd/C, NaHCO_3_, EtOH/THF (3:1), 28% (b) Chemical Synthesis of 2-Deoxy-2-fluoro Mannose 1-Phosphate **6**, Reagents and Conditions: (v) *p*-TsOH·H_2_O, MeOH, 77% (vi) BnBr, NaH, DMF, 60% (vii) CAN, Toluene/MeCN/H_2_O (1:1.5:1), 86% (viii) Diphenyl Phosphoryl Chloride, DMAP, CH_2_Cl_2_, 50% (ix) H_2_, PtO_2_, Pd­(OH)_2_, NaHCO_3_, EtOH/THF (3:1) Then Pd­(OH)_2_, H_2_O/MeOH (3:1), 82%; α-Linked Stereochemistry was Confirmed by Inspection of the ^3^ *J*_1,2_ Coupling Constants (**3**: *J* = 5.2 Hz, **6**: *J* = 6.0 Hz) (c) Chemical Synthesis of Fluorinated GTPs **8** and **9**, Reagents and Conditions: (x) POCl_3_, PO­(OMe)_3_, Then bis­(tributylammonium)­pyrophosphate, Bu_3_N, MeCN, 13% (xi) Cu­(OTf)_2_, Sodium Triflinate, ^t^BuOOH, H_2_O, 11%. Compounds **8** & **9** were Purified by Manual SAX Chromatography using DEAE-Sepharose with TEAB or Ammonium Bicarbonate Buffer, Respectively

The synthesis of 2-deoxy 2-fluoro mannose 1-phosphate **6** began from our recently reported 2-deoxy-2-fluoro mannose derivative **4** (Scheme 1b).^19^ Following similar protecting group manipulations, to remove the 4,6-benzylidene and install benzyl protecting groups, the anomeric *O*-*p*-methoxyphenol group was removed by treatment with cerium­(IV) ammonium nitrate, yielding hemiacetal **5** in 40% yield over three steps. Reaction of **5** with diphenyl phosphoryl chloride proceeded in 50% yield and, following global deprotection to remove the constituent OBn and OPh groups, delivered the target phosphate **6** in 82% yield. Finally, mannose 1-phosphate was synthesized as previously described (see Supporting Information for details),^20^ to provide material for native GDP-mannose synthesis.

We also sought to prepare GTPs featuring fluorination of the nucleobase or ribose moiety. First, a GTP analogue (**8**) featuring fluorination at the C2′ position of ribose was prepared from commercial material **7** (Scheme 1c). As outlined by Takenishi et al.,^21^ compound **7** was treated with POCl_3_ and conversion to the triphosphate completed by the addition of bis­(tributylammonium)­pyrophosphate.^22^ Reaction progress was monitored by analytical SAX-HPLC until full consumption of the dichlorophosphate intermediate was observed. The target triphosphate **8** was isolated in 13% yield, following flash column chromatography to remove excess pyrophosphate and trimethyl phosphate, before final purification by manual SAX chromatography using DEAE-Sepharose (to remove final traces of mono- and diphosphate). To incorporate fluorine into the guanine, we decided to retain native 2- and 6-position substituents and, upon noting an established instability of the 8-position C–F bond to basic conditions,^23,24^ targeted incorporation of C8-CF_3_ instead. Using modified Montesarchio *et al*. and Langlois *et al*. conditions,^25–27^ commercial GTP was converted to C8-CF_3_ GTP **9** in 13% yield, following purification by flash column and preparative SAX HPLC (Scheme 1c).

### Exploring Enzymatic Sugar Nucleotide Construction

We and others have previously evaluated the ability of *Se*GDP-Man-PP to accept modified mannose 1-phosphates.^17,28,29^ Relatedly, kinetic evaluation has been explored for Cps2L, a thymidylyltransferase from *Streptococcus pneumoniae*. Both enzymes demonstrated tolerance to fluorinated glucose, galactose, and mannose 1-phosphate configured substrates, producing the corresponding dTDP- and GDP-sugar nucleotides.^30^ However, to the best of our knowledge, there are no reports concerning the tolerance of *Se*GDP-Man-PP toward modified nucleoside triphosphates.

Accordingly, modified sugar nucleotide formation was evaluated using glycosyl 1-phosphates **3**, **6** and mannose 1-phosphate (**S3**) alongside triphosphates **8**, **9,** and GTP, first quantifying any formation using analytical SAX-HPLC (Table 1), before successful conversions were scaled up. *Se*GDP-Man-PP demonstrated 82% conversion of 3-deoxy 3-fluoro 1-phosphate **3** to the corresponding GDP-Man **10** after 16 h (Table 1, entry 1). Similarly, 2-deoxy 2-fluoro substrate **6** showed 91% conversion to **11** after 4 h (Table 1, entry 2). These results further ratify the promiscuity of *Se*GDP-Man-PP toward isosteric replacement of mannose ring hydroxyl groups with fluorine. Switching to modified GTPs and mannose 1-phosphate, no reaction was observed for C8-trifluoromethyl GTP **9** (Table 1, entry 3) with analytical SAX-HPLC indicating only hydrolysis of the triphosphate over 16 h (to the corresponding di- and monophosphate). Indicatively, this C8-modification might be too bulky to access the enzyme active site. Conversely, a smaller 2′-deoxy 2′-fluoro modification within GTP **8** was accepted by *Se*GDP-Man-PP, with SAX-HPLC analysis showing 92% conversion to the corresponding sugar nucleotide **13** after 4 h (Table 1, entry 4). Chemoenzymatic synthesis of compounds **10**, **11**, and **13** was successfully scaled to produce multimilligram quantities (37 mg for **10**, 65 mg for **11**, and 13 mg for **13**).

**Table 1:**
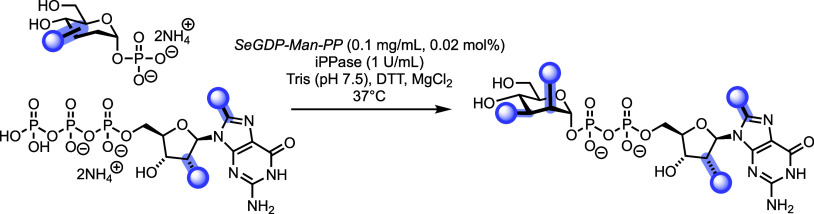

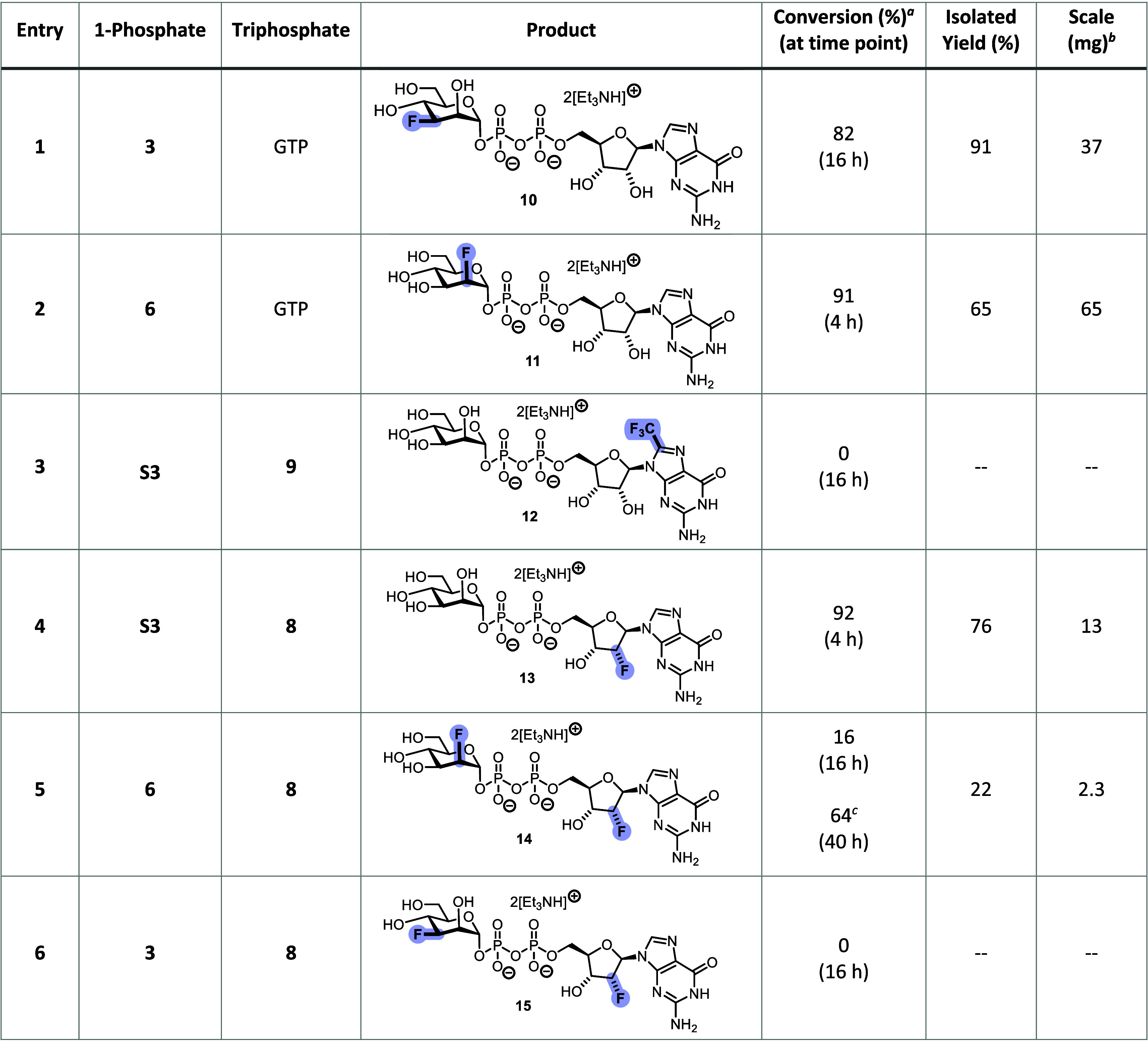
Enzymatic Synthesis of Variably Fluorinated GDP-Mannose Derivatives.^a^ ^,^ ^b^ ^,^ ^c^

a Percentage conversion measured by quantitative analytical SAX-HPLC.

b Of the isolated product.

c Enzyme concentration increased to 1.0 mg/mL (0.2 mol %) after 16 h. Sugar nucleotides **10**, **11**, **13**, and **14** were purified by preparative SAX-HPLC eluting with TEAB buffer.

Concurrently, we explored the ability of *Se*GDP-Man-PP to accept both a modified glycosyl 1-phosphate and modified GTP (Table 1, entry 5). Quadruple fluorination has been reported for contiguous substitution within the hexose/pentose framework of UDP-Gal systems,^14^ but multiple fluorination across a sugar nucleotide framework is hitherto unreported. Excitingly, upon pairing 2-deoxy 2-fluoro mannose 1-phosphate **3** with 2′-deoxy 2′-fluoro GTP **8**, formation of single sugar nucleotide product **14** was observed. After 16 h, low-level conversion was noted (16%) in the presence of 0.1 mg/mL of *Se*GDP-Man-PP; to improve conversion, the enzyme concentration was increased to 1.0 mg/mL, and the transformation left for a further 24 h, increasing conversion to 64%. This reaction was scaled up, purified by preparative SAX-HPLC, and the resulting bis-fluorinated sugar nucleotide **14** characterized. Observation of ^31^P­{^1^H} NMR doublets at δ_P_ = −11.4 ppm and δ_P_ = −14.0 ppm was complimented by two distinct ^19^F­{^1^H} chemical shifts (δ_F_ = −203.9 and −204.8 ppm), corresponding to 2′-F and 2″-F respectively (Figure 2A). Interestingly, when 3-deoxy 3-fluoro mannose 1-phosphate **3** was partnered with 2′-deoxy 2′-fluoro GTP **8** instead, no reaction to produce nucleotide sugar **15** was observed, with SAX analysis showing only background hydrolysis of the triphosphate (Table 1, entry 6). The increased reaction times required to synthesize both derivative **14** (40 h) and **10** (16 h) possibly indicate too steep a kinetic penalty to produce a bis-fluorinated GDP-mannose featuring 3″- and 2′-fluorination using *Se*GDP-Man-PP.

**Figure 2:**
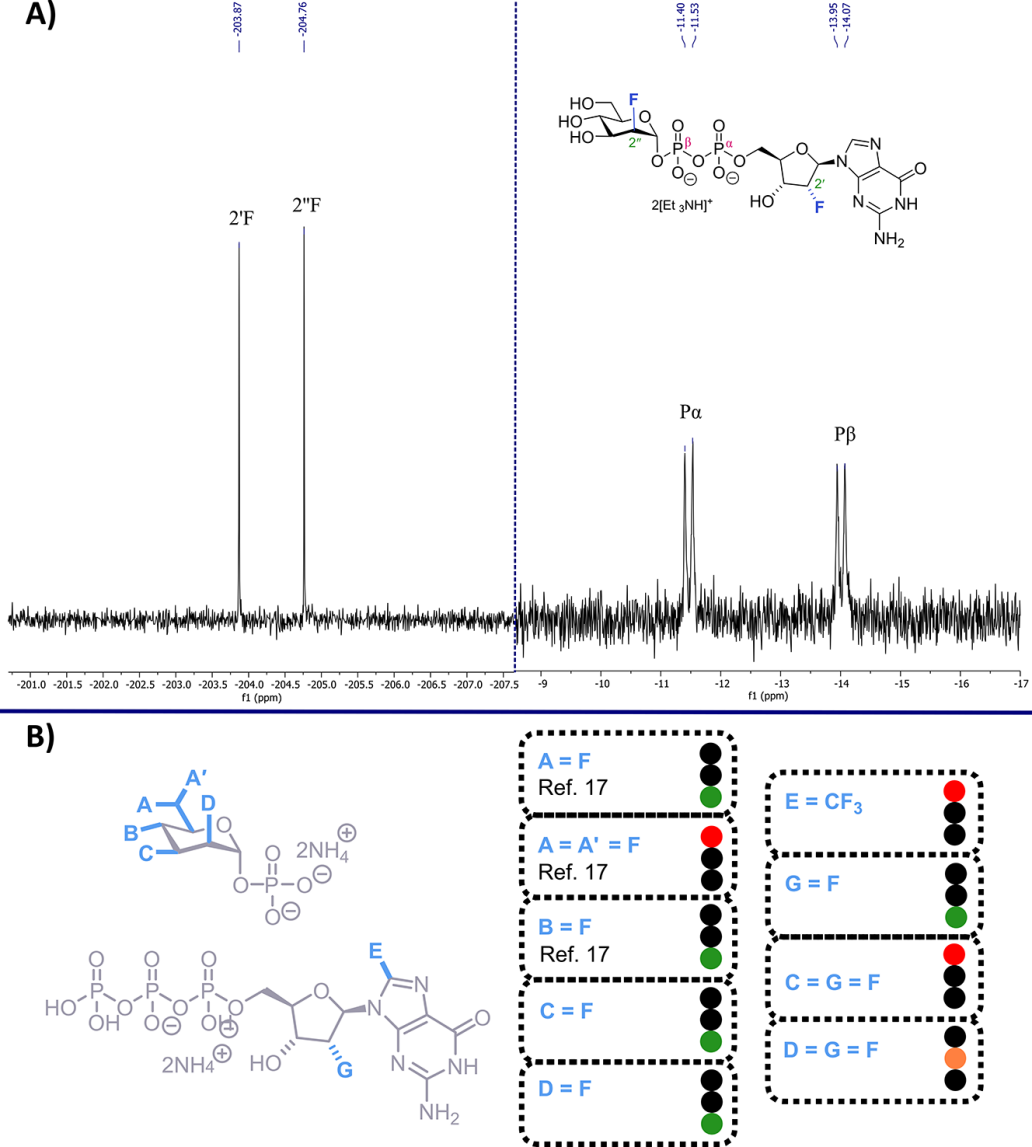
(A) ^19^F­{^1^H} NMR (377 MHz, left) and ^31^P­{^1^H} NMR (162 MHz, right) of bis-fluorinated sugar nucleotide **14** in D_2_O. (B) Expanded SAR profile for *Se*GDP-Man-PP toward variable fluorination within derived sugar nucleotides. Traffic light activity for substrate conversion to non-native GDP-Man indicated by good (green), moderate (amber), or none (red).

Taken together, these results represent a crucial development in the capability for *Se*GDP-Man-PP toward accessing non-native GDP-mannoses. Replacement of guanosine C2′-OH with fluorine is tolerated (**13** and **14**) and enables the first sugar nucleotides featuring a modification to the ribose component. A structural model of SeGDP-ManPP has been built using AlphaFold3 and compound **13** docked to this (see Supporting Information for details). While additional enzyme is required, the inclusion of fluorine on either side of the pyrophosphate linkage is possible, delivering a dual-fluorinated GDP-mannose. Figure 2B highlights an overall structure-to-function capability for *Se*GDP-Man-PP with regard to the inclusion of fluorine substituents.

### Evaluation of Fluorinated Sugar Nucleotides with GDP-Mannose Dehydrogenase

To demonstrate an initial use for fluorinated GDP-mannoses **10**, **11**, **13**, and **14**, we deployed them toward further understanding the sugar nucleotide binding requirements in the active site of the GDP-mannose dehydrogenase (GMD) from *Pseudomonas aeruginosa*, building on our previous explorations in this area.^17,28,31^ GMD is a member of a small group of NAD^+^-dependent four-electron-transfer dehydrogenases and oxidizes C6 of GDP-mannose to GDP-mannuronic acid. The activity and kinetic parameters for sugar nucleotides **10**, **11**, **13**, and **14** were thus evaluated against recombinant GMD, with the release of NAD­(H) monitored calorimetrically at 340 nm (Figure 3A).^32^

**Figure 3:**
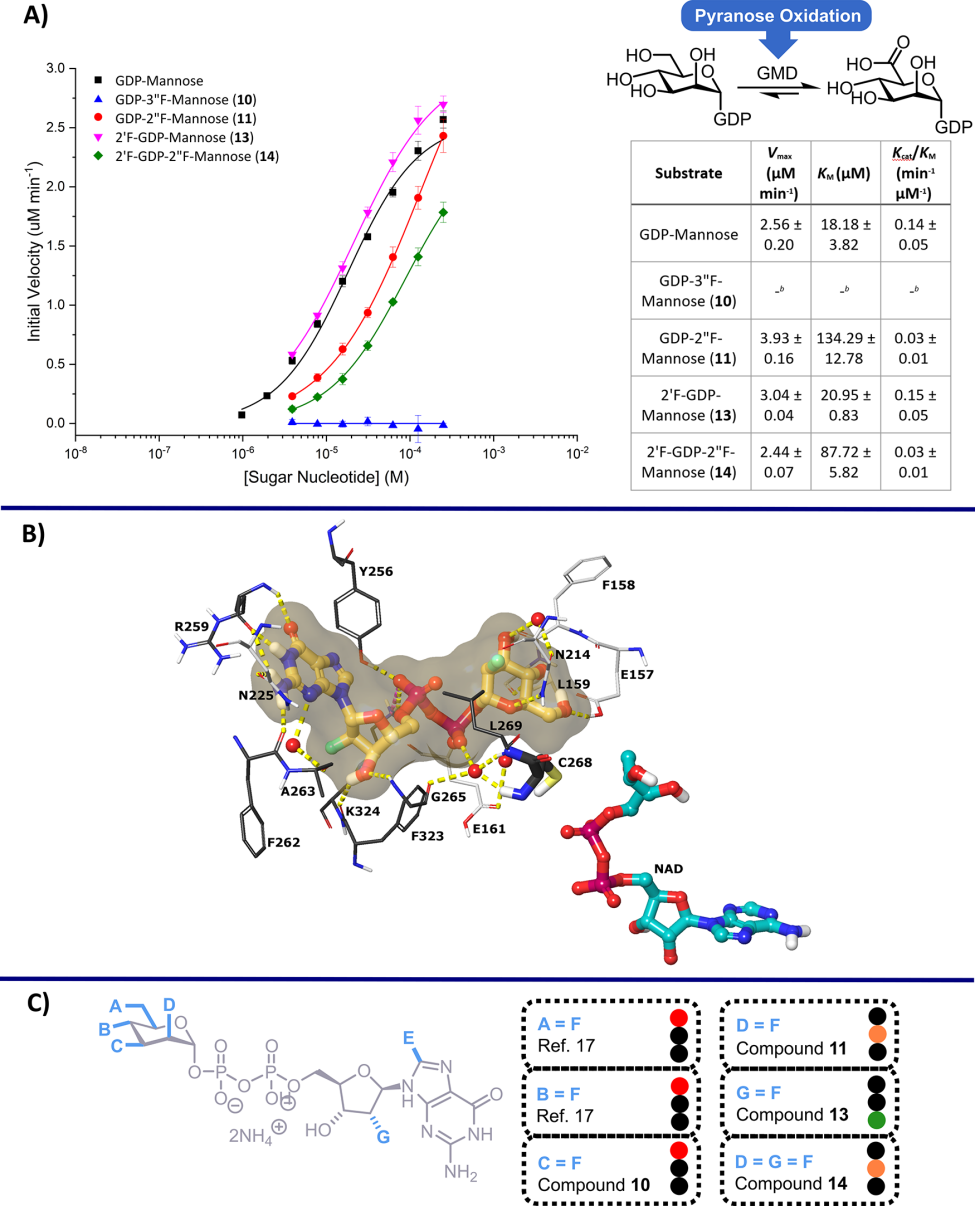
(A) Plot of average velocity versus substrate concentration for oxidation of sugar nucleotides GDP-mannose, **10**, **11**, **13**, and **14**. Data fitted using the Hill model (OriginPro 9.6), and error bars indicate the standard error of three measurements. (B) Predicted binding pose of **14** in the GMD active site. Ligand is displayed in stick-and-ball representation, amino acid residues as thin tubes, except C268 in thick tubes, water molecules as red spheres, electrostatic interactions as color dashed lines. Molecular surface of **14** is rendered light orange; GMD chain A residues are highlighted in light gray, chain B residues in dark gray, and NAD­(H) in teal. Docking score = −16.5 kcal/mol, Δ*G* = −63.5 kcal/mol for **14** versus −19.3 kcal/mol, Δ*G* = −69.4 kcal/mol for GDP-mannose. (C) Expanded SAR profile for GMD from *P. aeruginosa* toward fluorinated sugar nucleotides. Traffic light activity for substrate conversion indicated by good (green), moderate (amber), or none (red).

The observed *V*_max_ (maximum velocity) of analogues **11**, **13**, and **14** were very similar (∼2.5–4.0 μM min^–1^), indicating these were accepted as substrates by GMD. However, no activity was observed for 3″-deoxy-3″-fluoro analogue **10**. Analysis of the crystal structure of GMD (PDB: 1MV8) highlights C3″-OH (in the mannuronate oxidation product) forming a hydrogen bond with the amide backbone of Leu159, which may be critical for binding and thus explain why analogue **10** is not a substrate (see the Supporting Information for details of predicted binding pose of **10** in the GMD active site). This finding also builds upon a similar observation made by us regarding an inactive C4″-F GDP-mannose probe,^17^ noting that C4″-OH makes three hydrogen bonds with proximal Leu159 and Glu157 residues. Taken together, these data suggest that Leu159 is critical for GMD binding GDP mannose.

Beyond similar *V*_max_ values, perturbation in the observed *K*_M_ was seen for analogues **11** and **14**, featuring C2″-fluorination of mannose. C2″-OH of mannose is mostly exposed to solvent in the GMD crystal structure, forming weak hydrogen bonds with the side chains of Asn214 and His217. For analogues **11** and **14**, a 5–7.5-fold increase in *K*_M_ was observed, compared to GDP-mannose, suggesting perturbation of these weaker bonding associations impacts substrate binding. This trend continued for the derived specificity constant (catalytic efficiency, *K*_cat_/*K*_M_), where both substrates displayed a five-fold lower catalytic efficiency compared to GDP-mannose. Near identical kinetic parameters to those of the native substrate were observed for analogue **13**, featuring C2′-fluorination of the nucleoside. C2′-OH of the ribonucleoside is exposed to solvent in the GMD crystal structure and forms no hydrogen bonding interactions with the enzyme, so replacement with fluorine is possible.

Considering bis-fluorinated analogue **14** again, the observed specificity constant matched that observed for C2″-fluorinated analogue **11** (rather than **13**), reinforcing the contribution mannose C2″-OH has on substrate binding and GMD activity, versus ribose 2′-OH. However, we note also that double-fluorinated GDP-Man **14** has a lower *K*_m_ than single C2″-labeled analogue **11**, suggesting that there may be compensatory interactions when a second F is introduced to the molecule. Figure 3B illustrates the predicted binding of compound **14** in the GMD active site, where many of the contacts for the native ligand are retained, but fluorine substitution predictably removes contacts with H217 and Y257. The C2″-fluorine substitution indicates loss of a water-mediated H-bond with A263. Considering these data, we were able to further evolve a structure–activity relationship motif to reveal key positions and interactions within ligand-GMD association (Figure 3C).

## Conclusions

Using a chemoenzymatic strategy, a series of GDP-mannose analogues have been synthesized whereby sugar hydroxyl groups are systematically replaced by fluorine. Chemical synthesis enables the incorporation of fluorine into both the glycosyl 1-phosphate and nucleotide triphosphate components required as substrates for *Se*GDP-Man-PP-mediated sugar nucleotide assembly. Four novel fluorinated sugar nucleotides are produced using this approach on milligram scales and are then characterized and evaluated as tools to study the GDP-mannose dehydrogenase from *P. aeruginosa*, GMD. Replacement of C3″-OH in GDP-mannose with fluorine removes substrate binding capability. Substitution of the adjacent hydroxyl within mannose (C2″-OH) results in ∼five-fold loss in binding affinity and catalytic efficiency, while a first example of substitution of C2′-OH within the nucleotide results in no apparent effect on affinity. Development of these new probes thus reveals important structure-to-function information to support the development of inhibitors for GMD. Intriguingly, these results also indicate a C2′-F inclusion within GDP-Man is benign and could be targeted more widely as a fluorine-based probe within other pathways. For example, those that complete *de novo* GDP-fucose biosynthesis (GDP-mannose 4,6-dehydratase and GDP-keto-6-deoxymannose 3,5-epimerase/4-reductase) or other mannosyltransferases. Furthermore, incorporation of two fluorines at distinct positions across the sugar nucleotide motif (mannose and ribose) enables comparison of the kinetic effect of introducing fluorine at multiple sites; here demonstrating the capability to monitor the effect of pyranose versus pentose fluorination. These findings provide a capability to further explore such structure–activity tools and the influence the nucleotide component may play within substrate binding for biosynthetic transformations that utilize GDP-mannose sugar nucleotides as building blocks. They may also enable, in combination with other technologies, access to cell-penetrating nucleotide sugars.

## Supplementary Material



## Data Availability

The data that support the findings of this study have been included as part of the Supporting Information and are available from the corresponding author upon reasonable request.

## Notes

Full experimental procedures for the expression of *Sc*GDP-ManPP & *Pa*GMD; chemical synthesis of glycosyl 1-phosphates and fluorinated GTP’s; enzymatic synthesis of GDP-mannose analogues; plate based assay to determine GMD activity; and NMR data (^1^H, ^13^C, ^19^F, and ^31^P) (PDF)
