# Supplementary material for: Fluorinating the Sugar and the Nucleotide: Exploring Fluorination Within GDP-Mannose Probes Using Chemoenzymatic Synthesis
Source: JACS Au. 2025 Jul 31;5(8):3994–4001. doi: 10.1021/jacsau.5c00626 (PMC12381709; doi:10.1021/jacsau.5c00626)
Supplement: Supplementary file 1 [file au5c00626_si_001.pdf]

Supporting Information

Fluorinating the Sugar and the Nucleotide: Exploring Fluorination Within GDP-Mannose Probes Using Chemoenzymatic Synthesis

Jonathan P. Dolan,[1] Sean T. Evans,[1] Caecilie M. M. Benckendorff,[1] Suat Sari,[2] Aisling Ní Cheallaigh,[1] and Gavin J. Miller[1]*

[1]School of Chemical & Physical Sciences and Centre for Glycoscience, Keele University, Keele, Staffordshire, ST5 5BG, UK.
[2]Hacettepe University, Faculty of Pharmacy, Department of Pharmaceutical Chemistry, 06100, Ankara, Turkey.
*Authors for correspondence: g.j.miller@keele.ac.uk

General Experimental 2
Chemistry 2
Biochemistry 2
Chemical Synthesis of Glycosyl 1-phosphates 4
General procedure for SAX chromatography 4
Synthesis of α-D-Mannose 1-phosphate (S3) 4
Synthesis of 3-deoxy-3-fluoro mannose-1-phosphate (3) 5
Synthesis of 2-deoxy-2-fluoro mannose-1-phosphate (6) 8
Chemical Synthesis of Fluorinated GTP analogues 12
Expression of *Se*GDP-Man-PP (*rfbM)* 15
Enzymatic Synthesis of GDP-Mannose Analogues 16
Expression of *Pa*GMD 20
Kinetics Assay for GMD Activity 21
Molecular Docking 22
Results and discussion 22
References 25
NMR Spectra 27

General Experimental

Chemistry

All reagents and solvents which were available commercially were purchased from Sapala Organics, Biosynth, Acros, Alfa Aesar, Fisher Scientific, Sigma Aldrich or TCI. All reactions in non-aqueous solvents were conducted with oven-dried glassware with a magnetic stirring device under an inert atmosphere of nitrogen passed through a drying column using a vacuum manifold. Solvents were purified by passing through activated alumina columns and transferred under nitrogen unless otherwise stated. MeCN was obtained by drying over 3 Å molecular sieves and stored in a J Young® flask. Reactions requiring low temperatures used the following cooling baths: -78 °C (dry ice/acetone), -30 °C (dry ice/acetone), and 0 °C (ice/water). Reactions were followed by thin layer chromatography (TLC) using Merck silica gel 60 F_{254} analytical plates (aluminium support) and were developed using short wave UV radiation (245 nm) and 10% H_2SO_4 in methanol/Δ. Purification *via* flash column chromatography was conducted manually using Sigma Aldrich silica gel 60 (0.040-0.063 mm) under a positive pressure of compressed air. Analytical HPLC was performed using Agilent 1220 Infinity LC. Purification via strong ion exchange (SAX) chromatography was conducted on an Agilent 1260 Infinity II. Optical activities were recorded on a Bellingham and Stanley ADP430 polarimeter (concentration in g/100mL). HRMS were obtained on Agilent LC-QToF (UPLC 2390 Infinity II & QToF 6530B) employing electrospray (ES+) ionization or with the assistance of the NMSF at Swansea University.

^{1}H NMR spectra were recorded at 400 MHz, ^{13}C NMR spectra at 100 MHz, 31P NMR spectra at 161 MHz and ^{19}F NMR spectra at 376 MHz respectively using Bruker Magnet system 400'54 Ascend (400 MHz). ^{1}H NMR resonances were assigned with the aid of gDQCOSY. ^{13}C NMR resonances were assigned with the aid of gHSQCAD. Coupling constants are reported in Hertz. Chemical shifts (δ, in ppm) are standardised against the deuterated solvent peak. NMR data were analyzed using Mestrenova. ^{1}H NMR splitting patterns were assigned as follows: br.s (broad singlet), s (singlet), d (doublet), dd (doublet of doublets), ddd (doublet of doublet of doublets), app. t (apparent triplet), t (triplet), quartet (q) or m (multiplet and/or multiple resonances). Assignment of proton, carbon and phosphorous signals follow the numbering system illustrated below:

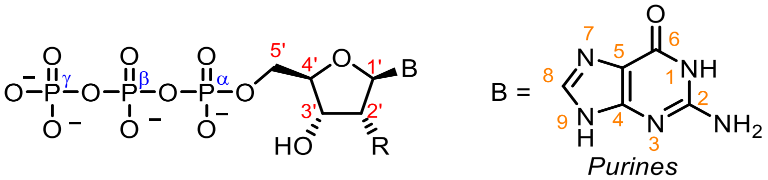

Biochemistry

Sterilisation of media and equipment was carried out in a Prestige Medical bench top autoclave at 121 °C for 15 minutes. Bacterial cultures were incubated in a SciQuip Benchtop INCU-Shake MIDI/MAXI or SciQuip Floor Standing INCU-Shake TL6-5R. LB-agar plates, 96-well assay plates and enzymatic reactions were incubated in a Grant-Bio Orbital Shaker Incubator ES-20, IKA KS4000i Control Incubator or Wolf Laboratories Galaxy R CO_2 Incubator. Centrifugation was performed using either a Sorvall RC5Cplus, SciQuip Sigma 2-16P, Eppendorf 5810 or Beckman Coulter™ Microfuge20. SDS-PAGE was carried out using Bio-Rad mini protean 3 apparatus using Bio-Rad mini-PROTEAN TGX precast gels. Proteins were concentrated either using 10k or 30k MWCO Amicon® Ultra-15 centrifugal filter device.

Analytical grade reagents were used as supplied by commercial suppliers. Bacterial plasmid DNA extraction & purification performed using GeneJET plasmid miniprep kit (ThermoFisher) following the manufactures recommended protocol. His tagged purification was performed on an ÄKTA Start fitted with a 5 mL Cytiva HisTrapFF column using the preset affinity chromatography method. Gel filtration chromatography was performed on an ÄKTA Start fitted with a HiPrep 16/60 Sephacryl S-200 HR column using the preset gel filtration chromatography method.

Chemical Synthesis of Glycosyl 1-phosphates

General procedure for SAX chromatography

A Bio Rad Bio-Scale™ Mini Macro-Prep® High Q Cartridge (5 mL) was flushed with degassed 1M aqueous ammonium formate (15 mL, 0.5 mL/min) followed by degassed water (15 mL, 0.5 mL/min). A solution of the compound to be purified (<50 mg) in degassed water (0.5 mL) was loaded onto the cartridge and the cartridge was flushed with degassed water (15 mL, 0.15 mL/min). The cartridge was then flushed with degassed 1M aqueous ammonium formate (15 mL, 0.15 mL/min) whilst collecting fractions (~2 mL). The fractions were then analysed by TLC and those containing product were combined and lyophilized. Repeated cycles of redissolving the dried compound in water and lyophilization was conducted to remove ammonium formate, affording the product.

Synthesis of α-D-Mannose 1-phosphate (S3)

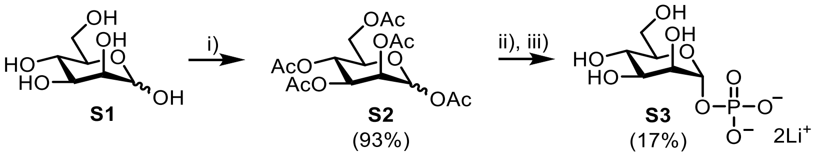

***Reaction conditions**: **i)** Ac_2O, DMAP, Pyr; **ii)** H_3PO_4 (crystalline), 60°C, high vacuum; **iii)** LiOH, THF/H_2O (1:1);*

1,2,3,4,6-Penta-O-Acetyl-α-D-Mannose (S2)

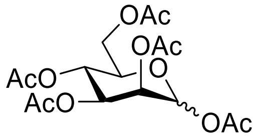

Mannose (5.01 g, 27.8 mmol, 1.0 eq.) and DMAP (0.10 g, 0.82 mmol, 0.03 eq.) were suspended in pyridine (70 mL) and cooled to 0°C. Acetic anhydride (18 mL, 190.8 mmol, 6.9 eq.) was added gradually over 2 minutes and allowed to stir at 0°C for 5 minutes before being allowed to warm to room temperature with vigorous stirring. After 2 hours, ice water (100 mL) was added and allowed to stir vigorously before the solvent was removed *in vacuo*. The residue was resuspended in 1:1 EtOAc/H_2O (100 mL), the layers separated, and aqueous layer washed with a further 50 mL of EtOAc. The organic extracts were combined washed with $NaHCO_3$ sat. sol. (3 × 50 mL) and brine (50 mL). The organic layer was dried over anhydrous $MgSO_4$, filtered and concentrated *in vacuo*. The crude residue was purified by flash column chromatography in 1:1 EtOAc/Hexane to yield **S2** with an anomeric ratio of 10:1 (α/β) (10.13 g, 26 mmol, 93 %).

Data is in accordance with those previously reported.[1]

Rf 0.21 (2:1 Hexane/EtOAc); **^{1}H NMR** (400 MHz; MeOD) δ 6.07 (1 H, dd, *J* = 1.9 Hz, **H_1**), 5.41–5.34 (2 H, m, **H_3**, **H_4**), 5.30 (1 H, dd, *J* = 3.2, 1.9 Hz, **H_2**), 4.31 (1 H, dd, *J* = 12.3, 4.4 Hz, **H_{6a}**), 4.10–4.19 (1 H, m, **H_5**), 4.11 (1 H, dd, *J* = 12.5, 2.5 Hz, **H_{6b}**), 2.21 (3 H, s, Ac), 2.20 (3 H, s, Ac), 2.09 (3 H, s, Ac), 2.09 (3 H, s, Ac), 2.01 (3 H, s, Ac); **^{13}C{^{1}H} NMR** (101 MHz; MeOD) δ 172.3 (C=O), 171.5 (C=O), 171.3 (C=O), 169.9 (C=O), 92.0 (**C_1**), 71.8 (**C_5**), 70.4 (**C_3**), 69.7 (**C_2**), 66.6 (**C_4**), 63.2 (**C_6**), 20.6 (Ac-Me), 20.6 (Ac-Me), 20.6 (Ac-Me), 20.5 (Ac-Me), 20.5 (Ac-Me); **HRMS** [ES+] found $[M+NH_4]^+$ 408.1504, $C_{16}H_{26}NO_{11}$ requires 408.1500, error −0.94 ppm;

α-D-Mannose-1-phosphate dilithium salt (S3)

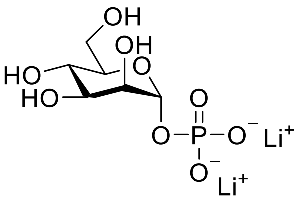

Peracetylated Mannose **S2** (10.1 g, 25.7 mmol, 1.0 eq.) and crystalline phosphoric acid (13.9 g, 141.6 mmol, 5.5 eq.) were separately dried in a vacuum desiccator over phosphorus pentoxide. After 24hrs they were combined in thoroughly dried glassware, placed under a strong vacuum (at least 0.16 bar) and gradually heated to 60°C. After 2 hours the melt was allowed to cool gradually to 0°C then suspended in dry THF (25 mL). The excess phosphoric acid was removed by precipitation with aq. 33% ammonium hydroxide (15.5 mL). After 5 minutes the solution was filtered and filter cake washed with THF (10 mL). Lithium hydroxide (3.15 g, 131.3 mmol, 5.1 eq.) was dissolved in water (25 mL) and added to the THF filtrate containing the peracetylated mannose 1-phosphate. After 90 minutes, the solution was neutralised with amberlite H^+ form, filtered and concentrated *in vacuo*. Methanol (30 mL) was added to the residue to precipitate mannose 1-phosphate. The precipitate was collected by centrifugation and the pellet washed twice with methanol (30 mL) then diethyl ether (30 mL) and dried under a stream of nitrogen to yield **S3** as only the α-anomer (1.13 g, 4.34 mmol, 17%).

Data is in accordance with those previously reported.[1]

^{1}H NMR (400 MHz; D_2O) δ 5.32 (1 H, d, *J* = 8.3 Hz, **H_1**), 3.98–3.90 (2 H, m, **H_2**, **H_3**), 3.90–3.83 (2 H, m, **H_5**, **H_{6a}**), 3.71 (1 H, dd, *J* = 12.1, 6.3 Hz, **H_{6b}**), 3.59 (1 H, t, *J* = 9.5 Hz, **H_4**); **^{13}C{^{1}H} NMR** (101 MHz; D_2O) δ 94.9 (**C_1**), 72.8 (**C_5**), 71.0 (d, *J* = 6.7 Hz, **C_2**), 70.0 (**C_3**), 67.0 (**C_4**), 61.2 (**C_6**); **^{31}P NMR** (162 MHz; D_2O) δ 1.76 (s, 1-OPO_3Li_2); **HRMS** [ES+] found $[M-H]^-$ 259.0225, $C_6H_{12}O_9P$ requires 259.0224, error 0.20 ppm;

Synthesis of 3-deoxy-3-fluoro mannose-1-phosphate (3)

Compound **1** was synthesised from 3-deoxy-3-fluoro glucose according to the procedure of Crich and Li.[2]

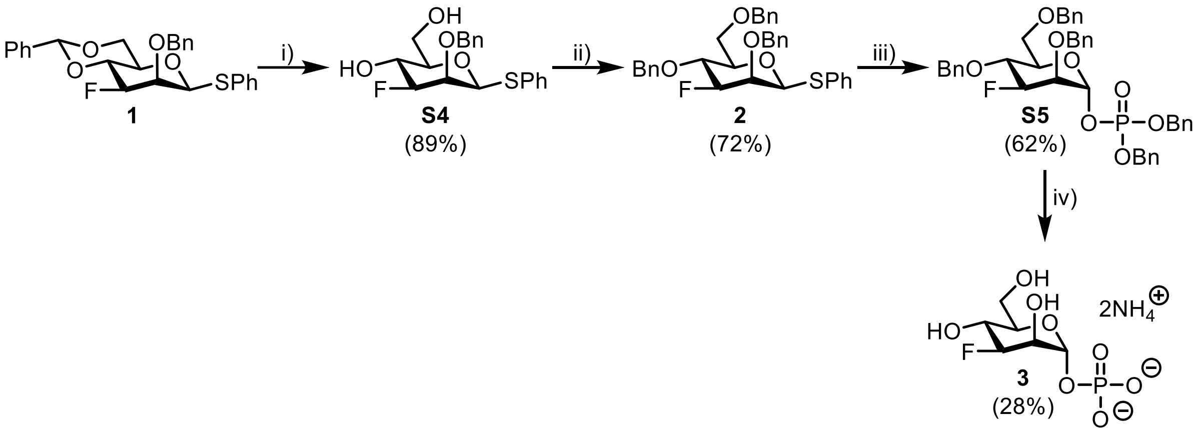

***Reaction conditions**: **i)** p-TsOH·H_2O, MeOH; **ii)** BnBr, NaH, DMF; **iii)** Dibenzyl phosphate, NIS, TfOH, CH_2Cl_2; **iv)** H_2, $Pd(OH)_2/C$, Pd/C, $NaHCO_3$ (5% aq.), EtOH:THF (3:1).*

Phenyl 2-O-benzyl-3-deoxy-3-fluoro-1-thio-β-D-mannopyranoside (S4)

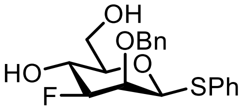

A stirred solution of compound **1** (1.27 g, 2.80 mmol, 1.0 eq.) and *p*-toluenesulfonic acid monohydrate (54 mg, 0.31 mmol, 0.11 eq.) in MeOH (12.5 mL) was heated to reflux for 2 h. The mixture was then cooled to 0 °C, neutralised with NEt_3 and concentrated *in vacuo*. The resulting residue was dissolved in CH_2Cl_2 (100 mL) and washed with water (100 mL). The organic layer was concentrated *in vacuo* and the resulting residue was purified by chromatography on silica gel (50% EtOAc/*n*-hexane) to afford title compound **S4** (904 mg, 2.48 mmol, 89%) as an off-white solid.

R_f 0.1 (40% EtOAc/*n*-hexane); **m.p.** 98-99 °C; **[α]**$_D^{22}$ −66 (c 1.0, $CHCl_3$); **^{1}H NMR** (400 MHz; $CDCl_3$) δ 7.48–7.42 (4H, m, Ar**H**), 7.39–7.26 (6H, m, Ar**H**), 4.93 (1H, d, *J* = 11.2 Hz, CH***H*** Bn), 4.82 (1H, dd, *J* = 1.4, 1.4 Hz, **H_1**), 4.76 (1H, *J* = 11.2 Hz, CH***H*** Bn), 4.52 (1H, ddd, *J* = 48.7, 9.3, 3.3 Hz, **H_3**), 4.30–4.18 (2H, m, **H_2**, **H_4**), 3.97–3.90 (1H, m, **H_{6a}**), 3.90–3.81 (1H, m, **H_{6b}**), 3.33 (1H, dddd, *J* = 9.7, 4.9, 3.4, 1.2 Hz, **H_5**), 2.64 (1H, d, *J* = 3.6 Hz, C6-O***H***), 2.29 (1H, dd, *J* = 6.7, 6.7 Hz, C4-O***H***); **^{13}C{^{1}H} NMR** (101 MHz; $CDCl_3$) δ 137.4 (**C_q**), 134.5 (**C_q**), 131.0 (**C**H), 129.1 (**C**H), 128.5 (**C**H), 128.3 (**C**H), 128.0 (**C**H), 127.6 (**C**H), 95.8 (d, $^1J_{C\text{-}F}$ = 189.5 Hz, **C_3**), 87.0 (d, $^3J_{C\text{-}F}$ = 8.4 Hz, **C_1**), 79.0 (d, $^3J_{C\text{-}F}$ = 6.6 Hz, **C_5**), 77.7 (d, $^2J_{C\text{-}F}$ = 15.5 Hz, **C_2**), 75.5 (d, $^4J_{C\text{-}F}$ = 4.1 Hz, **C**H_2 Bn), 66.7 (d, $^2J_{C\text{-}F}$ = 19.0 Hz, **C_4**), 62.4 (d, $^4J_{C\text{-}F}$ = 2.0 Hz, **C_6**); **^{19}F NMR** (376 MHz; $CDCl_3$) δ −195.7 (ddd, *J* = 49.0, 11.9, 5.4 Hz); **HRMS** [ES−] found $[M\text{-}H]^-$ 363.1071, $C_{19}H_{20}O_4FS$ requires 363.1072.

Phenyl 2,4,6-tri-*O*-benzyl-3-deoxy-3-fluoro-1-thio-β-D-mannopyranoside (2)

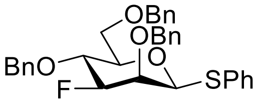

To a stirred solution of compound **S4** (100 mg, 0.274 mmol, 1.0 eq.) in DMF (1.1) mL at 0 °C was added sodium hydride (60% in mineral oil, 26 mg, 0.658 mmol, 2.4 eq.). After 15 min benzyl bromide (78 µL, 0.658 mmol, 2.4 eq.) was added dropwise and the solution was allowed to warm to room temperature. After stirring for 1 h, the reaction was cooled to 0 °C and slowly quenched with water. The mixture was diluted with CH_2Cl_2 (50 mL) and water (25 mL). The organic layer was separated and the aqueous layer was extracted with CH_2Cl_2 (2 × 25 mL). The combined organic layers were dried ($MgSO_4$) and concentrated *in vacuo*. Purification by chromatography on silica gel (10% EtOAc/*n*-hexane) afforded the title compound **2** (108 mg, 0.198 mmol, 72%) as a white solid.

R_f 0.3 (10% Et_2O/pet. ether); **m.p.** 92-93 °C; **[α]**$_D^{22}$ −27 (c 0.5, $CHCl_3$); **^{1}H NMR** (400 MHz; $CDCl_3$) δ 7.54–7.46 (4H, m, Ar**H**), 7.42–7.25 (13H, m, Ar**H**), 7.24–7.17 (3H, m, Ar**H**), 4.97 (2H, d, *J* = 11.4 Hz, 2 × C***H***H Bn), 4.85–4.79 (2H, m, 2 × C***H***H Bn), 4.76 (1H, dd, *J* = 1.4, 1.4 Hz, **H_1**), 4.75–4.49 (4H, m, **H_3**, 3 × CH***H*** Bn), 4.22 (1H, ddd, *J* = 6.1, 3.4, 1.1 Hz, **H_2**), 4.01 (1H, ddd, *J* = 10.8, 9.4, 9.4 Hz, **H_4**), 3.86 (1H, dd, *J* = 11.3, 1.6 Hz, **H_{6a}**), 3.74 (1H, dd, *J* = 11.0, 6.4 Hz, **H_{6b}**), 3.49 (1H, dddd, *J* = 9.6, 6.3, 1.7, 1.7 Hz, **H_5**); **^{13}C{^{1}H} NMR** (101 MHz; $CDCl_3$) δ 138.4 (**C_q**), 137.8 (**C_q**), 137.7 (**C_q**), 135.2 (**C_q**), 130.9 (**C**H), 129.0 (**C**H), 128.5 (**C**H), 128.4 (**C**H), 128.3 (**C**H), 128.3 (**C**H), 128.1 (**C**H), 127.9 (**C**H), 127.9 (**C**H), 127.8 (**C**H), 127.5 (**C**H), 127.3 (**C**H), 96.8 (d, $^1J_{C\text{-}F}$ = 191.6 Hz, **C_3**), 86.8 (d, $^3J_{C\text{-}F}$ = 8.4 Hz, **C_1**), 79.0 (d, $^3J_{C\text{-}F}$ = 8.1 Hz, **C_5**), 78.1 (d, $^2J_{C\text{-}F}$ = 15.7 Hz, **C_2**), 75.4 (d, $^4J_{C\text{-}}$

$_F$ = 4.1 Hz, **C**H_2 Bn), 74.8 (d, $^4J_{C\text{-}F}$ = 2.8 Hz, **C**H_2 Bn), 73.7 (d, $^2J_{C\text{-}F}$ = 17.3 Hz, **C$_4$**), 73.6 (**C**H_2 Bn), 69.5 (d, $^4J_{C\text{-}F}$ = 2.4 Hz, **C$_6$**); **^{19}F NMR** (376 MHz; $CDCl_3$) δ −191.3 (ddd, *J* = 48.8, 10.9, 6.2 Hz); **HRMS** [ES+] found $[M+NH_4]^+$ 562.2429, $C_{33}H_{37}FNO_4S$ requires 562.2427.

<u>Di-*O*-benzyl (2,4,6-tri-*O*-benzyl-3-deoxy-3-fluoro)-1-phosphate-α-D-mannopyranoside (S5)</u>

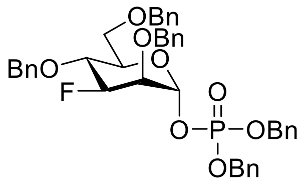

A solution of thioglycoside **2** (150 mg, 0.275 mmol, 1.0 eq.) and dibenzyl phosphate (153 mg, 0.551 mmol, 2.0 eq.) in anhydrous CH_2Cl_2 (3 mL) under an atmosphere of nitrogen was stirred over activated 4 Å molecular sieves for 30 min at room temperature. The mixture was then cooled to −40 °C and NIS (93 mg, 0.413 mmol, 1.5 eq.) was added followed by slow addition of TfOH (2.5 µL, 28 µmol, 0.1 eq.). The mixture was allowed to slowly warm up to 5°C over 2 hrs whilst stirring. The mixture was then filtered making sure to wash the molecular sieves thoroughly with CH_2Cl_2 (5 × 5 mL). The combined organic layers were washed with saturated aqueous $NaHCO_3$ (5 mL) and water (10 mL), dried ($MgSO_4$) and concentrated *in vacuo*. Purification by chromatography on silica gel (25% EtOAc/*n*-hexane) afforded the title compound **S5** (121 mg, 0.170 mmol, 62%) as a colourless oil.

R$_f$ 0.3 (30% EtOAc/*n*-hexane); **$[\alpha]_D^{22}$** +9 (c 1.0, $CHCl_3$); **^{1}H NMR** (400 MHz; $CDCl_3$) δ 7.36–7.17 (25H, m, Ar**H**), 5.73 (1H, ddd, *J* = 6.6, 4.8, 2.0 Hz, **H$_1$**), 5.10–4.93 (4H, m, 2 × C**H$_2$** Bn phosphate ester), 4.85–4.66 (3H, m, **H$_3$**, 2 × C***H***H Bn), 4.65–4.54 (2H, m, 2 × C***H***H Bn), 4.51 (1H, d, *J* = 10.9 Hz, C*H*H Bn), 4.43 (1H, d, *J* = 12.0 Hz, C***H***H Bn), 4.13 (1H, ddd, *J* = 11.3, 9.5, 9.5 Hz, **H$_4$**), 3.86–3.81 (1H, m, **H$_5$**), 3.76 (1H, ddd, *J* = 6.0, 3.4, 2.1 Hz, **H$_2$**), 3.69 (1H, dd, *J* = 11.1, 4.3, **H$_{6a}$**), 3.54 (1H, dd, *J* = 11.1, 1.9 Hz, **H$_{6b}$**); **^{13}C{^{1}H} NMR** (101 MHz; $CDCl_3$) δ 138.1 (**C**$_q$), 137.9 (**C**$_q$), 137.5 (**C**$_q$), 135.5 (2C, apparent dd, *J* = 9.6, 6.6 Hz, 2 × **C**$_q$ phosphate ester), 128.7 (**C**H), 128.6 (**C**H), 128.6 (**C**H), 128.4 (**C**H), 128.4 (**C**H), 128.3 (**C**H), 128.0 (**C**H), 128.0 (**C**H), 128.0 (**C**H), 127.8 (**C**H), 127.8 (**C**H), 127.8 (**C**H), 127.7 (**C**H), 127.6 (**C**H), 96.4 (dd, *J* = 8.3, 6.4, **C$_1$**), 92.6 (d, $^1J_{C\text{-}F}$ = 188.2 Hz, **C$_3$**), 75.6 (dd, *J* = 16.0, 9.8 Hz, **C$_2$**), 74.7 (d, *J* = 2.5 Hz, **C**H_2 Bn), 73.6-72.9 (3C, m, **C$_5$**, 2 × **C**H_2 Bn), 69.6 (2C, apparent dd, *J* = 10.2, 5.5 Hz, 2 × **C**H_2 Bn phosphate ester), 68.2 (d, $^4J_{C\text{-}F}$ = 1.4 Hz, **C$_6$**); **^{13}C-GATED** (101 MHz; $CDCl_3$): 96.4 ($^1J_{C1\text{-}H1}$ = 182 Hz, **C$_1$**); **^{19}F NMR** (376 MHz; $CDCl_3$) δ −201.7 (dddd, *J* = 49.3, 12.0, 5.8, 5.8 Hz); **^{31}P NMR** (162 MHz; $CDCl_3$) δ -2.80 (pd, *J* = 8.8, 6.5 Hz); **HRMS** [ES+] found $[M+Na]^+$ 735.2484, $C_{41}H_{42}FNaO_8P$ requires 735.2499.

<u>3-Deoxy-3-fluoro-α-D-mannose-1-phosphate (bis ammonium salt) (3)</u>

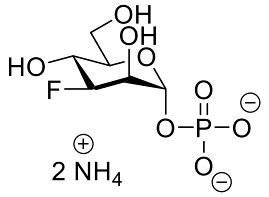

To a solution of compound **S5** (335 mg, 0.470 mmol, 1.0 eq.) in EtOH:THF (3:1, 5.36 mL) was added 5% (w/v) aqueous $NaHCO_3$ (0.67 mL), Pd/C (40 mg) and $Pd(OH)_2$/C (40 mg). The mixture was placed under an atmosphere of hydrogen by repeated cycles of vacuum and hydrogen using a three-way tap before being stirred at room temperature for 9 days. The reaction

mixture was then filtered through a syringe filter which was subsequently washed with water. The filtrate was concentrated *in vacuo* to afford a colourless oil. Purification by strong anion exchange chromatography (see **General experimental**) afforded the title compound **3** (39 mg, 0.132 mmol, 28%) as a white solid.

^{1}H NMR (400 MHz; D_2O) δ 5.40 (1H, ddd, *J* = 8.2, 5.2, 1.9 Hz, **H_1**), 4.91–4.75 (1H, m obscured by HDO peak, **H_3**), 4.29–4.19 (1H, m, **H_2**), 3.99–3.85 (3H, m, **H_4, H_5, H_{6a}**), 3.77 (1H, dd, *J* = 12.2, 5.2 Hz, **H_{6b}**); **^{13}C{^{1}H} NMR** (101 MHz; D_2O) δ 95.3 (dd, *J* = 8.2, 4.8 Hz, **C_1**), 92.0 (d, *J* = 181.2 Hz, **C_3**), 72.6 (d, *J* = 7.1 Hz, **C_5**), 69.1 (dd, *J* = 15.8, 7.8 Hz, **C_2**), 65.3 (d, *J* = 18.5 Hz, **C_4**), 60.6 (d, *J* = 1.9 Hz, **C_6**); **^{13}C-GATED** (101 MHz; D_2O): 95.3 ($^1J_{C1\text{-}H1}$ = 174 Hz, **C_1**); **^{19}F NMR** (376 MHz; D_2O) δ −204.0 (dddd, *J* = 48.9, 12.0, 6.1, 6.1 Hz); **^{31}P NMR** (162 MHz; D_2O) δ 0.36 (d, *J* = 8.4 Hz); **HRMS** [ES−] found $[M-H]^-$ 261.0181, $C_6H_{11}FO_8$ requires 261.0176.

Synthesis of 2-deoxy-2-fluoro mannose-1-phosphate (6)

Compound **4** was synthesised from β-D-glucose pentaacetate according to the procedure of Evans *et al.*[3]

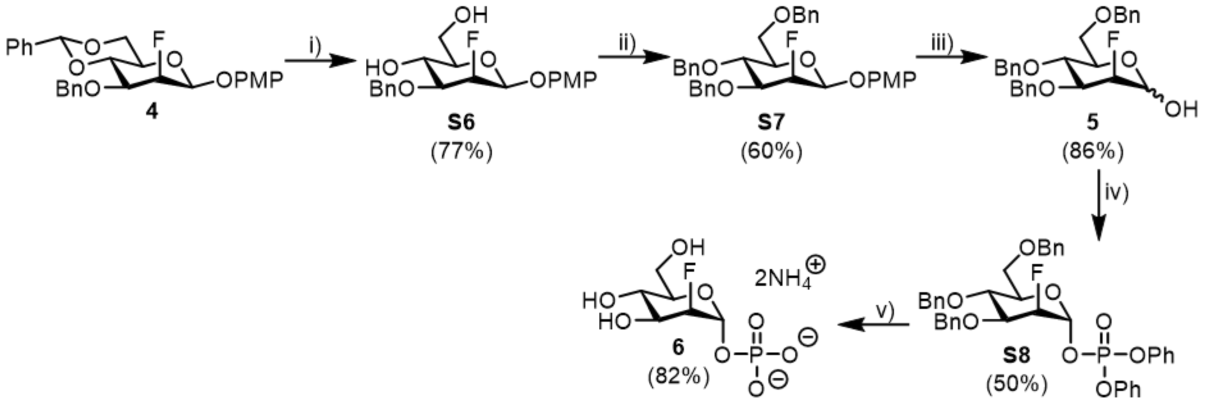

***Reaction conditions**: **i)** p-TsOH·H_2O, MeOH; **ii)** BnBr, NaH, DMF; **iii)** CAN, Tol:MeCN:H_2O (1:1.5:1); **iv)** diphenyl chlorophosphate, DMAP, CH_2Cl_2; **v)** PtO_2, $Pd(OH)_2$, $NaHCO_3$ (5% aq.), EtOH:THF (3:1) then $Pd(OH)_2$, H_2O:MeOH (3:1).*

p-Methoxyphenyl 3-*O*-benzyl-2-deoxy-2-fluoro-β-D-mannopyranoside (S6)

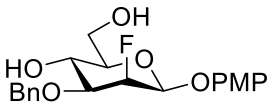

A stirred solution of compound **4** (288 mg, 0.617 mmol, 1 eq.) and *p*-TsOH·H_2O (240 mg, 1.26 mmol, 2.0 eq.) in MeOH (3 mL) was heated to reflux for 1 h. Saturated aqueous $NaHCO_3$ (5 mL) was added and the reaction mixture was concentrated *in vacuo*. The residue was dissolved in EtOAc (20 mL) and the organic layer was washed with water (10 mL). The organic layer was dried ($MgSO_4$) and concentrated *in vacuo*. The crude product was purified by chromatography on silica gel (70% EtOAc/*n*-hexane) to afford the title compound **S6** (180 mg, 0.475 mmol, 77%) as a white solid.

R_f 0.4 (70% EtOAc/*n*-hexane); **m.p.** 99-100 °C; **$[\alpha]_D^{20}$** −58 (c 1.0, $CHCl_3$); **^{1}H NMR** (400 MHz; $CDCl_3$) δ 7.46–7.40 (2H, m, Ar**H**), 7.38–7.23 (3H, m, Ar**H**), 7.05–6.96 (2H, m, Ar**H**), 6.86–6.78 (2H, m, Ar**H**), 5.11 (1H, d, *J* =18.4 Hz, **H_1**), 4.98 (1H, dd, *J* = 18.4 Hz, **H_2**), 4.78 (1H, d, *J* = 11.7 Hz, C*H*H Bn), 4.72 (1H, d, *J* = 11.7 Hz, C*H*H Bn), 3.91 (1H, dd, *J* = 12.1, 2.3 Hz, **H_{6a}**), 3.78 (1H, ddd,

J = 9.7, 9.6, 1.3 Hz, **H4**), 3.75–3.70 (1H, m, **H6b**), 3.72 (3H, s, OC**H3**), 3.58 (1H, ddd, J = 28.6, 9.4, 2.4 Hz, **H3**), 3.42 (1H, dddd, J = 9.7, 5.9, 2.4, 0.8 Hz, **H5**); **^{13}C{^{1}H} NMR** (101 MHz; $CDCl_3$) δ 155.3 (**C**q PMP), 151.1 (**C**q PMP), 138.2 (**C**q), 128.0 (**C**H), 127.6 (**C**H), 127.4 (**C**H), 117.3 (**C**H PMP), 114.2 (**C**H PMP), 97.5 (d, J = 15.3 Hz, **C1**), 87.2 (d, J = 187.1 Hz, **C2**), 80.1 (d, J = 17.2 Hz, **C3**), 77.1 (**C5**), 71.6 (**C**H_2 Bn), 66.2 (d, J = 1.7 Hz, **C4**), 61.2 (**C6**), 54.7 (OCH_3); **^{19}F NMR** (376 MHz; $CDCl_3$) δ −221.3 (ddd, J = 52.0, 28.7, 18.6 Hz); **HRMS** [ES+] found $[M+NH_4]^+$ 396.1825, $C_{20}H_{27}FNO_6$ requires 396.1822.

p-Methoxyphenyl 3,4,6-tri-*O*-benzyl-2-deoxy-2-fluoro-β-D-mannopyranoside (S7)

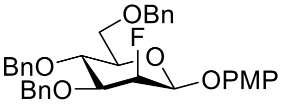

To a stirred solution of compound **S6** (1.02 g, 2.69 mmol, 1.0 eq.) in DMF (10.8 mL) at 0 °C was added NaH (60% in mineral oil , 258 mg, 6.46 mmol, 2.4 eq.). After 10 min BnBr (766 µL, 6.46 mmol, 2.4 eq,) was added dropwise and the mixture was stirred at room temperature overnight. Water (30 mL) was added to the reaction mixture and a white solid precipitated from solution. Recrystallisation of this solid from hot toluene afforded the title compound **S7** (907 mg, 1.62 mmol, 60%) as a white crystalline solid.

R$_f$ 0.6 (30% EtOAc/*n*-hexane); **m.p.** 166-167 °C decomposed; **[α]**$_D^{23}$ −20 (c 1.0, $CHCl_3$); **^{1}H NMR** (400 MHz; $CDCl_3$) δ 7.42–7.20 (15H, m, Ar**H**), 7.10–6.94 (2H, m, Ar**H** PMP), 6.82–6.73 (2H, m, Ar**H** PMP), 5.05–4.84 (3H, m, **H1**, **H2**, C**H**H Bn), 4.80 (1H, d, J = 11.8 Hz, C***H***H Bn), 4.72 (1H, d, J = 11.8 Hz, C***H***H Bn), 4.64–4.50 (3H, m, 3 × C***H***H Bn), 3.92 (1H, ddd, J = 9.5, 9.4, 1.1 Hz, **H4**), 3.84 (1H, dd, J = 11.0, 1.9 Hz, **H6a**), 3.75 (3H, s, OCH_3), 3.75-3.71 (1H, m, **H6b**), 3.65 (1H, ddd, J = 28.4, 9.3, 2.5 Hz, **H3**), 3.58 (1H, ddd, J = 9.8, 5.7, 1.7 Hz, **H5**); **^{13}C{^{1}H} NMR** (101 MHz; $CDCl_3$) δ 155.3 (**C**q), 151.0 (**C**q), 138.3 (**C**q), 138.0 (**C**q), 137.4 (**C**q), 128.6 (**C**H), 128.4 (**C**H), 128.3 (**C**H), 128.1 (**C**H), 128.1 (**C**H), 127.9 (**C**H), 127.9 (**C**H), 127.7 (**C**H), 127.5 (**C**H), 118.0 (**C**H), 114.5 (**C**H), 97.9 (d, $^{2}J_{C-F}$ = 15.5 Hz, **C1**), 87.0 (d, $^{1}J_{C-F}$ = 189.1 Hz, **C2**), 80.3 (d, $^{2}J_{C-F}$ = 17.6 Hz, **C3**), 75.8 (**C5**), 75.4 (**C**H_2 Bn), 74.4 (d, $^{3}J_{C-F}$ = 1.1 Hz, **C4**), 73.6 (**C**H_2 Bn), 71.9 (**C**H_2 Bn), 69.0 (**C6**), 55.6 (OCH_3); **^{19}F NMR** (376 MHz; $CDCl_3$) δ −219.0 (ddd, J = 51.4, 28.4, 18.0 Hz); **HRMS** [ES+] found $[M+Na]^+$ 581.2319, $C_{34}H_{35}FNaO_6$ requires 581.2315.

3,4,6-tri-*O*-benzyl-2-deoxy-2-fluoro-α/β-D-mannopyranoside (5)

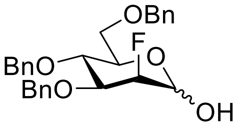

To a solution of compound **S7** (555 mg, 0.993 mmol, 1.0 eq.) in toluene:MeCN:H_2O (1:1.5:1, 19.9 mL) was added cerium(IV) ammonium nitrate (2.71 g, 4.94 mmol, 5.0 eq.). The mixture was stirred at room temperature for 1 h then was diluted with water (50 mL) and EtOAc (50 mL). The organic layer was separated, and the aqueous layer was extracted with EtOAc (5 × 20 mL). The combined organic layers were dried ($MgSO_4$) and concentrated *in vacuo*. Purification by chromatography on silica gel (20% EtOAc/*n*-hexane) afforded the title compound **5** (386 mg, 0.853 mmol, 86%, α:β=1:0.11) as an orange oil.

R$_f$ 0.3 (30% EtOAc/*n*-hexane); **α anomer ^{1}H NMR** (400 MHz; $CDCl_3$) δ 7.41–7.21 (13H, m, Ar**H**), 7.21–7.13 (2H, m, Ar**H**), 5.37–5.32 (1H, m, **H1**), 4.86 (1H, d, J = 10.9 Hz, C***H***H Bn), 4.79–4.61

(3H, m, C**H$_2$** Bn, **H$_2$**), 4.61–4.44 (3H, m, CH$_2$ Bn, C***H***H Bn), 4.04 (1H, ddd, *J* = 10.0, 6.1, 2.1 Hz, **H$_5$**), 3.92 (1H, ddd, *J* = 29.7, 9.4, 2.5 Hz, **H$_3$**), 3.78 (1H, ddd, *J* = 9.6, 9.6, 1.1 Hz, **H$_4$**), 3.73–3.59 (2H, m, **H$_{6a}$, H$_{6b}$**), 3.48–3.41 (1H, m, O**H**); **α anomer ^{13}C{^{1}H} NMR** (101 MHz; CDCl$_3$) δ 138.1 (**C**$_q$), 137.9 (**C**$_q$), 137.8 (**C**$_q$), 128.5 (**C**H), 128.4 (**C**H), 128.4 (**C**H), 128.0 (**C**H), 127.8 (**C**H), 127.7 (**C**H), 92.1 (d, $^2J_{C\text{-}F}$ = 29.7 Hz, **C$_1$**), 87.0 (d, $^1J_{C\text{-}F}$ = 175.9 Hz, **C$_2$**), 78.1 (d, $^2J_{C\text{-}F}$ = 17.2 Hz, **C$_3$**), 75.2 (**C**H$_2$ Bn), 74.6 (**C$_4$**), 73.4 (**C**H$_2$ Bn), 72.2 (**C**H$_2$ Bn), 71.2 (**C$_5$**), 69.2 (**C$_6$**); **α anomer ^{13}C-GATED** (101 MHz; CDCl$_3$): 92.1 ($^1J_{C1\text{-}H1}$ = 173 Hz, **C$_1$**);**^{19}F NMR** (376 MHz; CDCl$_3$) δ −204.5 (ddd, *J* = 49.9, 29.9, 7.1 Hz, α anomer), −222.8 (ddd, *J* = 50.8, 29.7, 18.0 Hz, β-anomer); **HRMS** [ES+] found [M+Na]$^+$ 475.1897, $C_{27}H_{29}FNaO_5$ requires 475.1897.

Di-*O*-phenyl (2-deoxy-2-fluoro-3,4,6-tri-*O*-benzyl)-1-phosphate-α-D-mannopyranoside (S8)

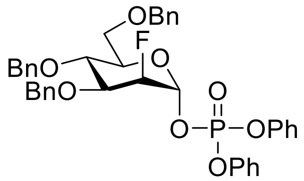

To a stirred solution of compound **5** (460 mg, 1.02 mmol, 1 eq.) and DMAP (283 mg, 2.31 mmol, 2.3 eq.) in CH_2Cl_2 (8.3 mL) at 0 °C was added diphenyl phosphoryl chloride (1.06 mL, 5.11 mmol, 5.0 eq.) and the reaction mixture was then stirred overnight at room temperature. The mixture was diluted with CH_2Cl_2 (50 mL) and washed with 1M HCl (25 mL), half saturated aqueous sodium bicarbonate (25 mL), water (25 mL) and concentrated *in vacuo*. The resulting residue was purified by sequential chromatographic purification (1→2% acetone/toluene then 40% Et_2O/pet. ether) to afford the title compound **S8** (352 mg, 0.514 mmol, 50%) as a colourless oil.

R$_f$ 0.2 (40% EtOAc/*n*-hexane); **[α]$_D$**^{23} +47 (c 1.0, CHCl$_3$); **^{1}H NMR** (400 MHz; CDCl$_3$) δ 7.38–7.23 (16H, m, Ar**H**), 7.23–7.12 (9H, m, Ar**H**), 6.06 (1H, ddd, *J* = 7.1, 5.3, 2.1 Hz, **H$_1$**), 4.84 (1H, d, *J* = 10.7 Hz, C***H***H Bn), 4.77–4.56 (4H, m, **H$_2$**, C**H$_2$** Bn, C***H***H Bn), 4.52 (1H, d, *J* = 10.7 Hz, C***H***H Bn), 4.45 (1H, d, *J* = 12.1 Hz, C***H***H Bn), 4.05 (1H, ddd, *J* = 9.7, 9.7, 1.1 Hz, **H$_4$**), 3.90–3.78 (2H, m, **H$_3$**, **H$_5$**), 3.71 (1H, dd, *J* = 11.2, 3.7 Hz, **H$_{6a}$**), 3.48 (1H, dd, *J* = 11.2, 1.8 Hz, **H$_{6b}$**); **^{13}C{^{1}H} NMR** (101 MHz; CDCl$_3$) δ 150.3 (dd, *J* = 7.0, 4.8 Hz, **C**$_q$ OPh), 138.0 (**C**$_q$), 137.6 (**C**$_q$), 130.0 (**C**H), 130.0 (**C**H), 129.8 (**C**H), 128.6 (**C**H), 128.5 (**C**H), 128.4 (**C**H), 128.1 (**C**H), 128.1 (**C**H), 127.9 (**C**H), 127.9 (**C**H), 127.7 (**C**H), 125.8 (d, *J* = 1.4 Hz, **C**H), 125.7 (d, *J* = 1.3 Hz, **C**H), 125.4 (d, *J* = 1.3 Hz, **C**H), 120.3–120.2 (m, **C**H), 96.4 (dd, $^2J_{C\text{-}F}$ = 32.3 Hz, $^2J_{C\text{-}P}$ = 5.7 Hz, **C$_1$**), 85.9 (dd, $^1J_{C\text{-}F}$ = 179.7 Hz, $^3J_{C\text{-}P}$ = 10.9 Hz, **C$_2$**), 77.5 (d, $^2J_{C\text{-}F}$ = 17.2 Hz, **C$_3$**), 75.5 (**C**H$_2$ Bn), 74.0 (**C$_5$**), 73.5 (**C**H$_2$ Bn), 73.5 (d, $^3J_{C\text{-}F}$ = 1.5 Hz, **C$_4$**), 72.4 (**C**H$_2$ Bn), 67.9 (**C$_6$**); **^{13}C-GATED** (101 MHz; CDCl$_3$): 96.4 ($^1J_{C1\text{-}H1}$ = 181 Hz, **C$_1$**); **^{19}F NMR** (376 MHz; CDCl$_3$) δ −202.8 (ddd, *J* = 49.0, 28.8, 5.4 Hz); **^{31}P NMR** (162 MHz; CDCl$_3$) −14.4 (d, *J* = 6.6 Hz); **HRMS** [ES+] found [M+Na]$^+$ 707.2170, $C_{39}H_{38}FNaO_8P$ requires 707.2186.

2-Deoxy-2-fluoro-α-D-mannose-1-phosphate (bis ammonium salt) (6)

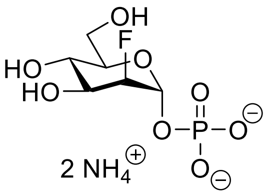

To a stirred solution of compound **S8** (314 mg, 0.456 mmol, 1.0 eq.) in EtOH:THF (3:1, 4.9 mL) was added PtO_2 (2 mg) and $Pd(OH)_2/C$ (37 mg). The mixture was placed under an atmosphere of hydrogen by repeated cycles of vacuum and hydrogen using a three-way tap before being stirred at room temperature for 4 days. The mixture was filtered through a syringe filter which was subsequently washed with water. The filtrate was concentrated *in vacuo* and redissolved in EtOH:THF (3:1, 4.9 mL) and PtO_2 (37 mg) and $Pd(OH)_2/C$ (37 mg) were added. The mixture was placed under an atmosphere of hydrogen and stirred at room temperature for a further 3 days. The mixture was again filtered and concentrated and was redissolved in H_2O:MeOH (3:1, 4.9 mL). PtO_2 (104 mg) was added and the mixture was placed under an atmosphere of hydrogen. The mixture was then stirred at room temperature for a further 12 days, sonicating for 30 min each day to prevent agglomeration of the catalyst. The mixture was filtered through a syringe filter which was subsequently washed with water. The filtrate was concentrated *in vacuo* to afford a colourless oil. Purification by strong anion exchange chromatography (see **General experimental**) afforded the title compound **6** (110 mg, 0.373 mmol, 82%) as a white solid.

Data is in accordance with those previously reported.[4]

^{1}**H NMR** (400 MHz; D_2O) δ 5.48 (1H, ddd, *J* = 8.4, 6.0, 2.2 Hz, **H$_1$**), 4.76 (1H, br. s, O***H***), 4.72 (1H, ddd, *J* = 49.6, 2.4, 2.4 Hz, **H$_2$**), 3.95 (1H, ddd, *J* = 31.2, 9.8, 2.6 Hz, **H$_3$**), 3.88-3.81 (2H, m, **H$_5$**, **H$_{6a}$**), 3.72 (1H, dd, *J* = 12.7, 6.1 Hz, **H$_{6b}$**), 3.68-3.60 (1H, m, **H$_4$**); ^{13}**C{^{1}H} NMR** (101 MHz; D_2O) δ 92.3 (dd, *J* = 29.9, 4.3 Hz, **C$_1$**), 90.3 (dd, *J* = 174.1, 8.0 Hz, **C$_2$**), 72.9 (**C$_5$**), 69.2 (d, *J* = 17.3 Hz, **C$_3$**), 66.7 (**C$_4$**), 60.6 (**C$_6$**); ^{19}**F NMR** (376 MHz; D_2O) δ −203.92 (ddd, *J* = 49.9, 31.2, 6.1 Hz); ^{31}**P NMR** (162 MHz; D_2O) 0.94 (d, *J* = 8.7 Hz); **HRMS** (ESI) *m/z* found: $[M-H]^-$ 261.0183 $C_6H_{11}FO_8$ requires 261.0176.

Chemical Synthesis of Fluorinated GTP analogues

General Method for Analytical SAX Reaction Monitoring

Mono- and triphosphorylation of nucleoside analogues was monitored *via* analytical HPLC.

System:

Column: Agilent PL-SAX 8µm 1000 Å, 150 x 4.6 mm

Flow rate: 0.5 mL/min

Time (min.)	%A (0.01 M NaCl)	%B (1.0 M NaCl)
**0.0**	100	0
**5.0**	75	25
**15.0**	40	60
**16.0**	0	100
**19.0**	0	100
**20.0**	100	0
**25.0**	100	0

Retention times: Uncharged molecules (~4 min); monophosphates (~8 min); diphosphates (~12 min); triphosphates (~16 min).

General Method for the Preparation of bis(tributylammonium)pyrophosphate

$Na_4P_2O_7$ (1.0 eq.) was dissolved in H_2O and loaded onto a H^+ resin column (DOWEX 50W-8X, H^+ form). The column was eluted with H_2O directly into a round-bottom flask containing Bu_3N (1.0 M in EtOH, 2.0 equiv.) at 0°C, until the eluent was pH ~7. The mixture was concentrated *in vacuo*, and the residue was transferred to a Falcon™ tube and lyophilised prior to use.

8-CF3-Guanosine Triphosphate (9)

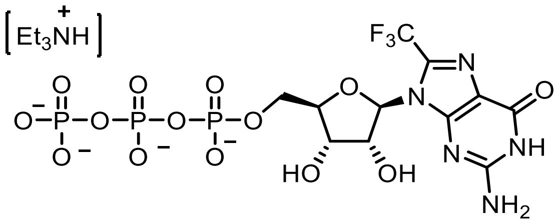

Guanosine 5'-*O*-triphosphate disodium salt (300 mg, 0.53 mmol, 1.0 eq.) was dissolved in H_2O (10.6 mL) and cooled to 0 °C. Sodium triflinate (165 mg, 1.06 mmol, 2.0 eq.) was added in one portion, followed by $Cu(OTf)_2$ (19 mg, 53 µmol, 0.1 eq.) and ^{t}BuOOH (70 wt.% in H_2O, 750 µL, 2.65 mmol, 5.0 eq.) dropwise. The reaction was stirred for a further 18 h at 0 °C, at which point the reaction mixture was concentrated *in vacuo* at 30 °C. The crude residue was purified *via* flash column chromatography on silica gel (0-60% NH_4OH/^{i}PrOH). The fractions containing NTP, visualised *via* TLC analysis (3:2 NH_4OH/^{i}PrOH) were pooled and concentrated *in vacuo* at 30 °C. The resultant residue was further purified *via* preparative SAX HPLC to afford the title compound (35 mg, 59 µmol, 11%) as a white amorphous solid, using the following method; Column: Agilent PL-SAX 1000 Å 150×25mm (PL1251-3102); Flow Rate: 15 mL/min; UV detection: 260 nm.

Time (min.)	%A (H_2O)	%B (2.0 M $NH_4HCO_{3(aq)}$)
**0.0**	100	0
**18**	70	30
**20**	50	50
**25**	50	50
**26**	100	0
**31**	100	0

^{1}H NMR (400 MHz, D_2O): δ 5.92 (1H, d, $^3J_{H1'-H2'}$ = 6.0 Hz, $H_{1'}$), 5.35 (1H, app. t, $^3J_{H2'-H1'/H3'}$ = 5.8 Hz, $H_{2'}$), 4.68 (1H, dd, $^3J_{H3'-H2'}$ = 5.4 Hz, $^3J_{H3'-H4'}$ = 2.1 Hz, $H_{3'}$), 4.43 – 4.28 (3H, m, $H_{4'}$, $H_{5'a}$, $H_{5'b}$), 3.20 (0.56H, q, J = 7.3 Hz, CH_3CH_2OH), 1.28 (0.86H, t, J = 7.3 Hz, CH_3CH_2OH); **^{13}C NMR** (101 MHz, D_2O): δ 161.2 (C_6), 156.5 (C_2), 153.3 (C_4), 135.3 (q, $^2J_{C8-F}$ = 38.6 Hz, C_8), 117.8 (q, $^1J_{CF3-F}$ = 270.6 Hz, CF_3), 116.3 (C_5), 89.0 ($C_{1'}$), 83.8 (d, $^3J_{C4'-P\alpha}$ = 8.7 Hz, $C_{4'}$), 70.9 ($C_{2'}$), 70.0 ($C_{3'}$), 65.4 (d, $^2J_{C5'-P\alpha}$ = 5.6 Hz, $C_{5'}$), 46.7 (CH_3CH_2OH), 8.3 (CH_3CH_2OH); **^{19}F NMR** (377 MHz, D_2O): δ −61.2; **^{31}P NMR** (162 MHz, D_2O): δ −5.9 (d, $J_{P\gamma-P\beta}$ = 15.3 Hz, P_γ), −10.7 (d, $J_{P\alpha-P\beta}$ = 15.2 Hz, P_α), −19.2 (t, $J_{P\beta-P\alpha/P\gamma}$ = 15.4 Hz, P_β); **HRMS** (NSI) *m/z* calculated for $C_{11}H_{14}F_3N_5O_{14}P_3$ $[M-H]^-$ 589.9708, found 589.9706.

<u>2’-deoxy 2’-fluoro Guanosine Triphosphate (8)</u>

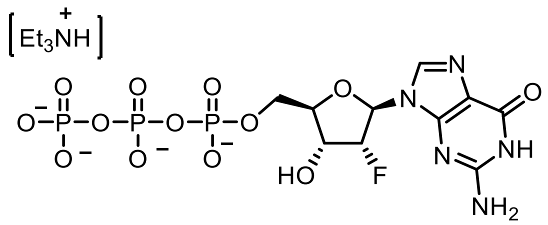

To an oven-dried two-necked round bottom flask equipped with a magnetic stirrer bar, 2’-deoxy-2’-fluoroguanosine **7** (250 mg, 0.88 mmol, 1.0 eq.) was added, and the reaction flask was evacuated and refilled with N_2 three times. The nucleoside was suspended in $PO(OMe)_3$ (1.2 mL), and the mixture was cooled to −20 °C (ice/NaCl bath). $POCl_3$ (99 µL, 1.06 mmol, 1.2 equiv.) was added dropwise, and the reaction mixture was allowed to warm to –10 °C and stirred for 6 h, at which point majority consumption of the starting material was observed *via* analytical SAX HPLC (see general analytical SAX HPLC method). The reaction mixture was cooled to −20 °C, and a precooled to −20 °C solution of bis(tributylammonium)pyrophosphate (966 mg, 1.76 mmol, 2.0 eq.) and Bu_3N (1.26 mL, 5.28 mmol, 6.0 eq.) in MeCN (4.4 mL) was added in one portion. The reaction mixture was allowed to slowly warm to 0 °C over 1 h, at which point complete consumption of the monophosphate intermediate was observed *via* analytical SAX HPLC (see general analytical SAX HPLC method). The reaction was quenched by the addition of H_2O (2 mL), and the mixture was stirred for a further 1 h at 0 °C. The aqueous mixture was extracted with DCM (3 x 10 mL) and the aqueous phase was concentrated *in vacuo* at 30 °C. The material was purified *via* flash column chromatography on silica gel (0-60% $NH_4OH/^iPrOH$). The fractions containing NTP, visualised *via* TLC analysis (3:2 $NH_4OH/^iPrOH$) were pooled and concentrated *in vacuo* at 30 °C. The residue was dissolved in a minimal amount of H_2O and loaded onto a column packed with DEAE-Sepharose™ and eluted with 0-40% 2M TEAB/H_2O. The fractions containing NTP, visualised *via* TLC analysis (3:2 $NH_4OH/^iPrOH$) were pooled and concentrated *in vacuo* at 30 °C. The resultant residue was dissolved in a minimal amount of H_2O and MeOH was added and then concentrated *in vacuo*

at 30 °C (this was repeated until a stable mass was obtained, indicating the successful removal of excess TEAB buffer), and the resultant purified NTP was lyophilised to afford the title compound (70 mg, 12 mmol, 13%) as a white amorphous solid.

^{1}H NMR (400 MHz, D_2O): δ 8.04 (1H, s, H_8), 6.20 (1H, dd, $^{3}J_{H1'-F}$ = 17.9 Hz, $^{3}J_{H1'-H2'}$ = 1.9 Hz, $H_{1'}$), 5.40 (1H, ddd, $^{2}J_{H2'-F}$ = 52.4 Hz, $^{3}J_{H2'-H3'}$ = 4.6 Hz, $^{3}J_{H2'-H1'}$ = 1.9 Hz, $H_{2'}$), 4.87 (1H, ov. ddd, $^{3}J_{H3'-F}$ = 21.0 Hz, $^{3}J_{H3'-H4'}$ = 7.1 Hz, $^{3}J_{H3'-H4'}$ = 4.5 Hz, $H_{3'}$), 4.46 – 4.26 (3H, m, $H_{4'}$, $H_{5'a}$, $H_{5'b}$); **^{13}C NMR** (101 MHz, D_2O) δ 158.8 (C_6), 153.9 (C_2), 93.7 (d, $^{1}J_{C2'-F}$ = 186.2 Hz, $C_{2'}$), 86.1 (d, $^{2}J_{C1'-F}$ = 34.0 Hz, $C_{1'}$), 81.4 (d, $^{3}J_{C4'-P\alpha}$ = 9.5 Hz, $C_{4'}$), 67.9 (d, $^{2}J_{C3'-F}$ = 15.7 Hz, $C_{3'}$), 63.8 (d, $^{2}J_{C5'-P\alpha}$ = 5.9 Hz, $C_{5'}$); **^{19}F NMR** (377 MHz, D_2O): δ −203.3 (ddd, $^{2}J_{F-H2'}$ = 52.2 Hz, $^{3}J_{F-H3'}$ = 21.2 Hz, $^{3}J_{F-H1'}$ = 17.9 Hz); **^{31}P{^{1}H} NMR** (162 MHz, D_2O): δ −8.25 (d, $^{2}J_{P\gamma-P\beta}$ = 19.3 Hz, P_γ), − 11.05 (d, $^{2}J_{P\alpha-P\beta}$ = 19.1 Hz, P_α), −22.10 (t, $^{2}J_{P\beta-P\alpha/P\gamma}$ = 19.3 Hz, P_β); **HRMS** (NSI) *m/z* calculated for $C_{10}H_{14}FN_5O_{13}P_3$ $[M-H]^-$ 523.9790, found 523.9790.

Expression of *Se*GDP-Man-PP (*rfbM)*

A clone encoding for GDP-α-D-mannose-pyrophosphorylase from *S. enterica* was kindly donated by T. L. Lowary.[5]

The plasmid encoding GDP-Man-PP was transformed into BL21(DE3)pLysS and grown in 2 × 1 L of Studier Autoinduction-LB (Studier, 2005) at 37 °C (180 rpm) in baffled flasks until an OD_{600} of 0.5 was reached, then the incubator temperature was reduced to 18 °C. After incubation overnight, the bacterial cell pellet was harvested by centrifugation (10,000 *x g*, 10 mins, 4 °C). The cell pellet was stored at –80 °C until required for purification.

The cell pellet was resuspended in Lysis buffer (100 mM Tris (pH 7.8), 100 mM NaCl) plus 20 mM imidazole, 1U DNase A and 10 µg/mL Lysozyme. Cell suspension was lysed by sonication (40% power, 2 secs on, 5 secs off, 30 cycles, 10 °C max) and cell debris cleared by centrifugation (20,000 *x g*, 60 mins 4 °C). Cell lysate was decanted and further clarified by filtration through 0.22 µm syringe filter. Clarified lysate applied to 5 mL Cytiva HisTrap column preequilibrated with Lysis buffer plus 20 mM imidazole. Elution performed following an imidazole gradient (25 mL each elution, 50, 100, 150, 200, 300, 500, 1000 mM imidazole in Lysis buffer). Fractions containing purified GDP-Man-PP visualised by SDS-PAGE, combined and concentrated using centrifugal concentrator (Amicon Ultra 10K MWCO). Concentrated protein was desalted using a Cytiva PD-10 column equilibrated and eluted with 50 mM Tris (pH 7.8), 200 mM NaCl. Protein eluant was concentrated using centrifugal concentrator (Amicon Ultra 10K MWCO) to ~10 mg/mL (concentration determined by nanodrop, MW ~ 55 KDa, ε_{280nm} = 72,685 M^{-1} cm^{-1}).

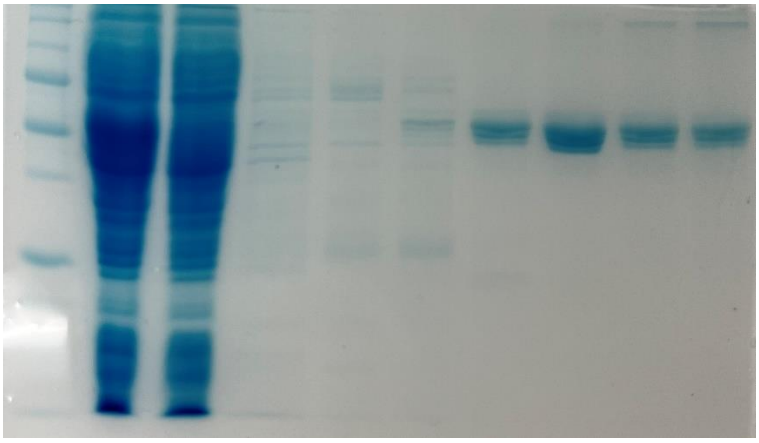

Figure S1: *Lane 1: Ladder, Lane 2: Lysate, Lane 3: Flowthrough from Ni-NTA, Lane 4: 20 mM Imidazole elution, Lane 5: 50 mM Imidazole elution, Lane 6: 100 mM Imidazole elution, Lane 7: 150 mM Imidazole elution, Lane 8: 200 mM Imidazole elution, Lane 9: 300 mM Imidazole elution, Lane 10: 500 mM Imidazole elution.*

Enzymatic Synthesis of GDP-Mannose Analogues

GDP-3′′-deoxy-3′′-fluoro-mannose (10)

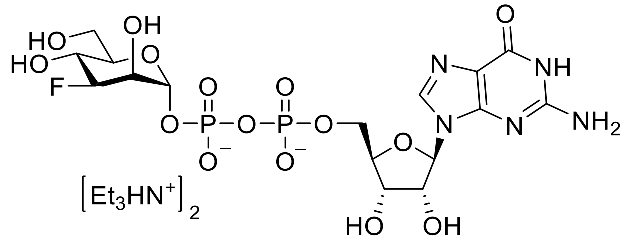

Free Acid
Chemical Formula: $C_{16}H_{24}FN_5O_{15}P_2$
Exact Mass: 607.0728
Molecular Weight: 607.3339

Di-Triethylamine Salt
Chemical Formula: $C_{28}H_{54}FN_7O_{15}P_2$
Exact Mass: 809.3137
Molecular Weight: 809.7199

A 15 mL falcon tube was charged with 3-deoxy-3-fluoro-mannose-1-phosphate diammonium salt (14.8 mg, 50 µmol, 10.0 mM, 1.0 eq.) and GTP disodium salt (35.4 mg, 62.5 µmol, 12.5 mM, 1.25 eq.) then suspended in 4.35 mL of deionised H_2O. The reaction was made up first with 500 µL of a 10× stock of the reaction buffer (stock concs.: 1 M Tris (pH 7.5), 2 M NaCl; final concs.: 100 mM Tris (pH 7.5), 200 mM NaCl), 10 µL of DTT (stock conc. 1 M; final conc. 2 mM), and 50 µL of $MgCl_2$ (stock conc. 1 M; final conc. 10 mM), followed by 50 µL of iPPase (Sigma, stock conc. 100 U/mL; final conc. 1 U/mL) and 49.5 µL of *Se*GDPManPP (stock conc. 10.1 mg/mL; final conc. 0.1 mg/mL; 0.02 mol%). The mixture was gently mixed and incubated at 37 °C and monitored by analytical SAX-HPLC. Once the reaction had reached completion (*ca.* 4 hrs), methanol (5 mL) was added, mixed and left at −20 °C for 4 hours. Following centrifugation to remove the precipitated enzymes, the supernatant was concentrated *in vacuo* and purified by preparative SAX-HPLC according to the following method. Column: Agilent PL-SAX 1000 Å 150×25mm (PL1251-3102); Eluents: A) H_2O, B) 2M TEAB (pH 9.5); Method: Column pre equilibrated with 0%B and held at 0%B for 5 mins, gradient to 50%B over 30 mins, hold at 50%B for 5 mins, gradient to 0%B over 3 mins, hold at 0%B for 5 mins; Flow Rate: 15 mL/min; Injection volume: 200 µL; Sample concentration: 50 mg/mL; UV detection: 260 nm.

Yield: 36.8 mg (45.4 µmol, 91%); **^{1}H NMR** (400 MHz; D_2O) δ 8.10 (1 H, s, **H$_8$**), 5.93 (1 H, d, $^3J_{H1'-H2'}$ = 5.8 Hz, **H$_{1'}$**), 5.56 (1 H, ddd, $^3J_{H1''-P\beta}$ = 7.6 Hz, $^4J_{H1''-F}$ = 5.1 Hz, $^3J_{H1''-H2''}$ = 2.0 Hz, **H$_{1''}$**), 4.77 (1 H, ddd, $^2J_{H3''-F}$ = 48.6 Hz, $^3J_{H3''-H4''}$ = 9.4 Hz, $^3J_{H3''-H2''}$ = 3.5 Hz, **H$_{3''}$**), 4.74 (1 H, t$_{app}$, $^3J_{H2'-H1'/H3'}$ = 5.5 Hz, **H$_{2'}$**), 4.51 (1 H, dd, $^3J_{H3'-H2'}$ = 5.2 Hz, $^3J_{H3'-H4'}$ = 3.6, **H$_{3'}$**), 4.35 (1 H, dt, $^3J_{H4'-H5a'}$ = 5.4 Hz, $^3J_{H4'-H3'/H5b'}$ = 2.6 Hz, **H$_{4'}$**), 4.29 (1 H, ddd, $^3J_{H2''-F}$ = 6.0 Hz, $^3J_{H2''-H3''}$ = 3.5 Hz, $^3J_{H2''-H1''}$ = 2.1 Hz, **H$_{2''}$**), 4.22 (2 H, dt, $^3J_{H5a'-H4'}$ = 4.9 Hz, $^3J_{H5b'-H4'}$ = 2.7 Hz, **H$_{5'}$**), 3.98 (1 H, dt, $^3J_{H4''-F}$ 12.6 Hz, $^3J_{H4''-H5''/H3''}$ = 9.7 Hz, **H$_{4''}$**), 3.89 (1 H, dt, $^2J_{H6a''-H6b''}$ = 11.3 Hz, $^3J_{H6a''-H5''}$ = 2.4 Hz, **H$_{6a''}$**), 3.88 (1 H, dd, $^3J_{H5''-H4''}$ = 9.9 Hz, $^3J_{H5''-H6''}$ = 1.9 Hz, **H$_{5''}$**), 3.78 (1 H, dd, $^2J_{H6b''-H6a''}$ = 12.6 Hz, $^3J_{H6b''-H5''}$ = 5.2, **H$_{6b''}$**), 3.72 (12 H, s, $DN^+($**<u>CH_2</u>**$CH_3)_3$), 1.92 (18 H, s, $DN^+(CH_2$**<u>CH_3</u>**$)_3$); **^{13}C{^{1}H} NMR** (100 MHz; D_2O) δ 158.9 (**C$_6$**), 153.9 (**C$_2$**), 151.7 (**C$_4$**), 137.5 (**C$_8$**), 96.4 (dd, $^2J_{C1''-P\beta}$ = 8.2 Hz, $^3J_{C1''-F}$ = 5.8 Hz, **C$_{1''}$**), 91.7 (d, $^1J_{C3''-F}$ = 182.1 Hz, **C$_{3''}$**), 86.9 (**C$_{1'}$**), 83.6 (d, $^3J_{C4'-P\alpha}$ = 8.9 Hz, **C$_{4'}$**), 73.8 (**C$_{2'}$**), 73.2 (d, $^3J_{C5''-F}$ = 7.2 Hz, **C$_{5''}$**), 70.3 (**C$_{3'}$**), 68.4 (dd, $^2J_{C2''-F}$ = 16.2 Hz, $^3J_{C2''-P\beta}$ = 9.4 Hz, **C$_{2''}$**), 65.3 (d, $^2J_{C5'-P\alpha}$ = 5.6 Hz, **C$_{5'}$**), 64.9 (d, $^2J_{C4''-F}$ = 18.6 Hz, **C$_{4''}$**), 60.3 (**C$_{6''}$**), 59.5 ($DN^+($**<u>CH_2</u>**$CH_3)_3$), 23.3 ($DN^+(CH_2$**<u>CH_3</u>**$)_3$); **^{19}F NMR** (377 MHz; D_2O) δ −204.4 (ddt, $^2J_{F-H3''}$ = 48.8 Hz, $^3J_{F-H4''}$ = 12.0 Hz, $^3J_{F-H2''}$ = 6.0 Hz); **^{31}P NMR** (162 MHz; D_2O) δ −11.4 (d, $^2J_{P\alpha-P\beta}$ = 20.1 Hz, Pα), −13.9 (dd, $^2J_{P\beta-P\alpha}$ = 20.5 Hz, $^3J_{P\beta-H1''}$ = 7.9 Hz, Pβ); **HRMS** [ES-] found $[M-H]^-$ 606.0658, $C_{16}H_{24}FN_5O_{15}P_2$-H requires 606.0655, error 0.49 ppm;

GDP-2''-deoxy-2''-fluoro-mannose (11)

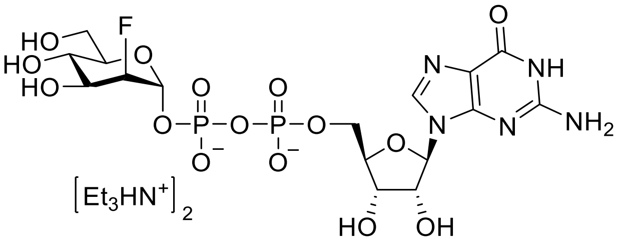

**Free Acid**	**Di-Triethylamine Salt**
Chemical Formula: $C_{16}H_{24}FN_5O_{15}P_2$	Chemical Formula: $C_{28}H_{54}FN_7O_{15}P_2$
Exact Mass: 607.0728	Exact Mass: 809.3137
Molecular Weight: 607.3339	Molecular Weight: 809.7199

A 15 mL falcon tube was charged with 2-deoxy-2-fluoro-mannose-1-phosphate diammonium salt (36.3 mg, 123 µmol, 10.0 mM, 1.0 eq.) and GTP disodium salt (87 mg, 153 µmol, 12.5 mM, 1.25 eq.) then suspended in 9.5 mL of deionised H_2O. The reaction was made up first with 2.46 mL of a 10× stock of the reaction buffer (stock concs.: 1 M Tris (pH 7.5), 2 M NaCl; final concs.: 100 mM Tris (pH 7.5), 200 mM NaCl), 24.6 µL of DTT (stock conc. 1 M; final conc. 2 mM), and 123 µL of $MgCl_2$ (stock conc. 1 M; final conc. 10 mM), followed by 123 µL of iPPase (Sigma, stock conc. 100 U/mL; final conc. 1 U/mL) and 122 µL of *Se*GDPManPP (stock conc. 10.1 mg/mL; final conc. 0.1 mg/mL; 0.02 mol%). The mixture was gently mixed and incubated at 37 °C and monitored by analytical SAX-HPLC. Once the reaction had reached completion (*ca.* 4 hrs), methanol (12.3 mL) was added, mixed and left at −20 °C for 4 hours. Following centrifugation to remove the precipitated enzymes, the supernatant was concentrated *in vacuo* and purified by preparative SAX-HPLC according to the following method. Column: Agilent PL-SAX 1000 Å 150×25mm (PL1251-3102); Eluents: A) H_2O, B) 2M TEAB (pH 9.5); Method: Column pre equilibrated with 0%B and held at 0%B for 5 mins, gradient to 50%B over 30 mins, hold at 50%B for 5 mins, gradient to 0%B over 3 mins, hold at 0%B for 5 mins; Flow Rate: 15 mL/min; Injection volume: 200 µL; Sample concentration: 50 mg/mL; UV detection: 260 nm.

Yield: 65.2 mg (80.5 µmol, 65%); **^{1}H NMR** (400 MHz; D_2O) δ 8.10 (1 H, s, **H_8**), 5.93 (1 H, d, $^3J_{H1'-H2'}$ = 6.2 Hz, **$H_{1'}$**), 5.69 (1 H, ddd, $^3J_{H1''-P\beta}$ = 8.2 Hz, $^3J_{H1''-F}$ = 6.0 Hz, $^3J_{H1''-H2''}$ = 2.1 Hz, **$H_{1''}$**), 4.89 (1 H, dt, $^2J_{H2''-F}$ = 50.0 Hz, $^3J_{H2''H1''}$ = 2.3 Hz, **$H_{2''}$**), 4.51 (1 H, dd, $^3J_{H3'-H2'}$ = 5.2 Hz, $^3J_{H3'-H4'}$ = 3.4 Hz, **$H_{3'}$**), 4.35 (1 H, td, $^3J_{H4'H3'}$ = 3.5 Hz, $^3J_{H4'-H5'}$ = 2.0 Hz, **$H_{4'}$**), 4.24–4.19 (2 H, m, **$H_{5'}$**), 3.99 (1 H, ddd, $^3J_{H3''-F}$ = 30.9 Hz, $^3J_{H3''-H4''}$ = 9.8 Hz, $^3J_{H3''-H2''}$ = 2.6 Hz, **$H_{3''}$**), 3.89–3.84 (2 H, m, **$H_{5''}$**, **$H_{6a''}$**), 3.82–3.76 (1 H, dd, $^3J_{H6b''H6a''}$ = 12.4 Hz, $^3J_{H6b''-H5''}$ = 4.8 Hz, **$H_{6b''}$**), 3.74 (1 H, td, $^3J_{H4''-H5''}$ = 9.0 Hz, $^3J_{H4''-H3''}$ = 1.0, **$H_{4''}$**); **^{13}C{^{1}H} NMR** (100 MHz; D_2O) δ 160.3 (**C_6**), 159.5 (**C_2**), 154.3 (**C_4**), 137.5 (**C_8**), 116.4 (**C_5**), 93.5 (d, $^2J_{C1''-F}$ = 28.4 Hz, **$C_{1''}$**), 89.5 (d, $^1J_{C2''-F}$ = 173.8 Hz, **$C_{2''}$**), 86.7 (**$C_{1'}$**), 83.7 (d, $^3J_{C4'-P\alpha}$ = 11.1 Hz, **$C_{4'}$**), 73.6 (**$C_{5''}$**), 73.5 (**$C_{2'}$**), 70.4 (**$C_{3'}$**), 69.1 (d, $^2J_{C3''-F}$ = 17.2 Hz, **$C_{3''}$**), 66.3 (**$C_{4''}$**), 65.3 (d, $^2J_{C5'-P\alpha}$ = 5.7 Hz, **$C_{5'}$**), 60.2 (**$C_{6''}$**), 59.0 (DN^+(**<u>CH_2</u>**$CH_3)_3$), 46.6 (DN^+(**<u>CH_2</u>**$CH_3)_3$), 8.2 ($DN^+(CH_2$**<u>CH_3</u>**$)_3$), 7.4 ($DN^+(CH_2$**<u>CH_3</u>**$)_3$); **^{19}F NMR** (377 MHz; D_2O) δ −204.7 (ddd, $^2J_{F-H2''}$ = 49.0 Hz, $^3J_{F-H3''}$ = 30.8 Hz, $^3J_{F-H1''}$ = 6.0 Hz); **^{31}P NMR** (162 MHz; D_2O) δ −11.5 (dt, $^2J_{P\alpha-P\beta}$ = 21.2 Hz, $^3J_{P\alpha-H5'}$ = 4.8 Hz, Pα), −14.0 (dd, $^2J_{P\alpha P\beta}$ = 20.8 Hz, $^3J_{P\beta-H1''}$ = 8.2 Hz, Pβ); **HRMS** [ES-] found $[M-H]^-$ 606.0643, $C_{16}H_{24}FN_5O_{15}P_2$-H requires 606.0655, error −2.00 ppm;

2′-deoxy-2′-fluoro-GDP-mannose (13)

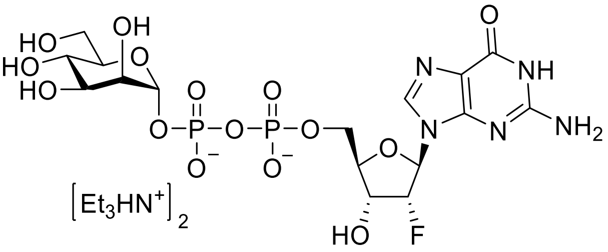

**Free Acid**	**Di-Triethylamine Salt**
Chemical Formula: $C_{16}H_{24}FN_5O_{15}P_2$	Chemical Formula: $C_{28}H_{54}FN_7O_{15}P_2$
Exact Mass: 607.0728	Exact Mass: 809.3137
Molecular Weight: 607.3339	Molecular Weight: 809.7199

A 15 mL falcon tube was charged with mannose-1-phosphate dilithium salt (9.45 mg, 34.7 µmol, 12.5 mM, 1.6 eq.) and 2′-deoxy-2′-fluoro-GTP disodium salt (15.8 mg, 21.7 µmol, 7.8 mM, 1.0 eq.) then suspended in 2.41 mL of deionised H_2O. The reaction was made up first with 278 µL of a 10× stock of the reaction buffer (stock concs.: 1 M Tris (pH 7.5), 2 M NaCl; final concs.: 100 mM Tris (pH 7.5), 200 mM NaCl), 5.5 µL of DTT (stock conc. 1 M; final conc. 2 mM), and 28 µL of $MgCl_2$ (stock conc. 1 M; final conc. 10 mM), followed by 28 µL of iPPase (Sigma, stock conc. 100 U/mL; final conc. 1 U/mL) and 28 µL of *Se*GDPManPP (stock conc. 10.1 mg/mL; final conc. 0.1 mg/mL; 0.02 mol%). The mixture was gently mixed and incubated at 37 °C and monitored by analytical SAX-HPLC. Once the reaction had reached completion (*ca.* 4 hrs), methanol (2.78 mL) was added, mixed and left at −20 °C for 4 hours. Following centrifugation to remove the precipitated enzymes, the supernatant was concentrated *in vacuo* and purified by preparative SAX-HPLC according to the following method. Column: Agilent PL-SAX 1000 Å 150×25mm (PL1251-3102); Eluents: A) H_2O, B) 2M TEAB (pH 9.5); Method: Column pre equilibrated with 0%B and held at 0%B for 5 mins, gradient to 50%B over 30 mins, hold at 50%B for 5 mins, gradient to 0%B over 3 mins, hold at 0%B for 5 mins; Flow Rate: 15 mL/min; Injection volume: 200 µL; Sample concentration: 50 mg/mL; UV detection: 260 nm.

Yield: 13.3 mg (16.4 µmol, 76%); **^{1}H NMR** (400 MHz; D_2O) δ 8.04 (1 H, s, **H$_8$**), 6.23 (1 H, dd, $^{3}J_{H1'\text{-}F}$ 17.7, $^{3}J_{H1'\text{-}H2'}$ = 2.3 Hz, **H$_{1'}$**), 5.52 (1 H, dd, $^{3}J_{H1''\text{-}P\beta}$ = 8.0 Hz, $^{3}J_{H1''\text{-}H2''}$ = 2.1 Hz, **H$_{1''}$**), 5.44 (1 H, ddd, $^{2}J_{H2'\text{-}F}$ = 52.2 Hz, $^{3}J_{H2'\text{-}H3'}$ = 4.6 Hz, $^{3}J_{H2'\text{-}H1'}$ = 2.2 Hz, **H$_{2'}$**), 4.79 (1 H, ddd, $^{3}J_{H3'\text{-}F}$ = 19.0 Hz, $^{3}J_{H3'\text{-}H4'}$ = 7.4 Hz, $^{3}J_{H3'\text{-}H2'}$ = 4.8 Hz, **H$_{3'}$**), 4.40 – 4.31 (2 H, m, **H$_{4'}$**, **H$_{5a'}$**), 4.25 (1 H, ddd, $^{2}J_{H5b'\text{-}H5a'}$ = 12.1 Hz, $^{3}J_{H5b'\text{-}P\alpha}$ = 6.1 Hz, $^{3}J_{H5b'\text{-}H4'}$ = 4.1 Hz, **H$_{5b'}$**), 4.05 (1 H, dd, $^{3}J_{H2''\text{-}H3''}$ = 3.5 Hz, $^{3}J_{H2''\text{-}H1''}$ = 2.0 Hz, **H$_{2''}$**), 3.92 (1 H, dd, $^{3}J_{H3''\text{-}H4''}$ = 9.8, $^{3}J_{H3''\text{-}H2''}$ = 3.4 Hz, **H$_{3''}$**), 3.89 – 3.83 (2 H, m, **H$_{5''}$**, **H$_{6a''}$**), 3.75 (1 H, dd, $^{2}J_{H6b''\text{-}H6a''}$ = 12.6 Hz, $^{3}J_{H6b'\text{-}H5'}$ = 5.6 Hz, **H$_{6b''}$**), 3.73 (2 H, s, $DN^{+}(\underline{\mathbf{CH_2}}CH_3)_3$), 3.68 (1 H, t$_{app}$, $^{3}J_{H4''\text{-}H3''/H5''}$ = 9.8 Hz, **H$_{4''}$**), 1.92 (3 H, s, $DN^{+}(CH_2\underline{\mathbf{CH_3}})_3$); **^{13}C{^{1}H} NMR** (100 MHz; D_2O) δ 158.9 (**C$_6$**), 153.9 (**C$_2$**), 151.1 (**C$_4$**), 137.5 (**C$_8$**), 96.5 (d, $^{2}J_{C1''\text{-}P\beta}$ = 5.8 Hz, **C$_{1''}$**), 93.4 (d, $^{1}J_{C2'\text{-}F}$ = 186.8 Hz, **C$_{2'}$**), 86.2 (d, $^{3}J_{C1'\text{-}F}$ = 34.1 Hz, **C$_{1'}$**), 81.6 (d, $^{3}J_{C4'\text{-}F}$ = 9.4 Hz, **C$_{4'}$**), 73.7 (**C$_{5''}$**), 70.2 (d, $^{3}J_{C2''\text{-}P\beta}$ = 9.2 Hz, **C$_{2''}$**), 69.8 (**C$_{3''}$**), 68.3 (d, $^{2}J_{C3'\text{-}F}$ = 15.9 Hz, **C$_{3'}$**), 66.4 (**C$_{4''}$**), 64.2 (d, $^{2}J_{C5'\text{-}P\alpha}$ = 5.4 Hz, **C$_{5'}$**), 60.7 (**C$_{6''}$**), 59.4 ($DN^{+}(\underline{\mathbf{CH_2}}CH_3)_3$), 23.2 ($DN^{+}(CH_2\underline{\mathbf{CH_3}})_3$); **^{19}F NMR** (377 MHz; D_2O) δ −203.93 (dt, $^{2}J_{F\text{-}H2'}$ = 52.3 Hz, $^{3}J_{F\text{-}H1'/H3'}$ = 18.8 Hz); **^{31}P NMR** (162 MHz; D_2O) δ −11.45 (d, $^{2}J_{P\alpha\text{-}P\beta}$ = 20.8 Hz, Pα), −13.76 (dd, $^{2}J_{P\beta\text{-}P\alpha}$ = 20.7 Hz, $^{3}J_{P\beta\text{-}H1''}$ = 6.1 Hz, Pβ); **HRMS** [ES-] found $[M\text{-}H]^{-}$ 606.0656, $C_{16}H_{24}FN_5O_{15}P_2$-H requires 606.0655, error 0.16 ppm;

2′-deoxy-2′-fluoro-GDP-2″-deoxy-2″-fluoro-mannose (14)

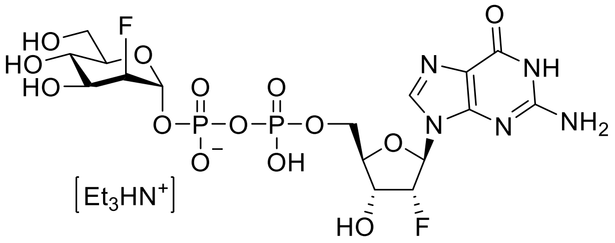

Free Acid
Chemical Formula: $C_{16}H_{23}F_2N_5O_{14}P_2$
Exact Mass: 609.0685
Molecular Weight: 609.3253

Mono-Triethylamine Salt
Chemical Formula: $C_{22}H_{38}F_2N_6O_{14}P_2$
Exact Mass: 710.1889
Molecular Weight: 710.5183

A 15 mL falcon tube was charged with 2-deoxy-2-fluoro-mannose-1-phosphate diammonium salt (5.3 mg, 17.9 µmol, 12.5 mM, 1.25 eq.) and 2′-deoxy-2′-fluoro-GTP disodium salt (10.5 mg, 14.4 µmol, 10.0 mM, 1.0 eq.) then suspended in 1.25 mL of deionised H_2O. The reaction was made up first with 144 µL of a 10× stock of the reaction buffer (stock concs.: 1 M Tris (pH 7.5), 2 M NaCl; final concs.: 100 mM Tris (pH 7.5), 200 mM NaCl), 2.9 µL of DTT (stock conc. 1 M; final conc. 2 mM), and 14.4 µL of $MgCl_2$ (stock conc. 1 M; final conc. 10 mM), followed by 14.4 µL of iPPase (Sigma, stock conc. 100 U/mL; final conc. 1 U/mL) and 14.3 µL of *Se*GDPManPP (stock conc. 10.1 mg/mL; final conc. 0.1 mg/mL; 0.02 mol%). The mixture was gently mixed and incubated at 37 °C and monitored by analytical SAX-HPLC. After 16hrs, a further 143 µL of *Se*GDP Man PP (stock conc. 10.1 mg/mL; final conc. 1.1 mg/mL; 0.2 mol%) was added. Once the reaction had reached completion (*ca.* 40 hrs), ethanol (1.5 mL) was added, mixed and left at −20 °C for 4 hours. Following centrifugation to remove the precipitated enzymes, the supernatant was concentrated *in vacuo* and purified by preparative SAX-HPLC according to the following method. Column: Agilent PL-SAX 1000 Å 150×25mm (PL1251-3102); Eluents: A) H_2O, B) 2M TEAB (pH 9.5); Method: Column pre equilibrated with 0%B and held at 0%B for 5 mins, gradient to 50%B over 30 mins, hold at 50%B for 5 mins, gradient to 0%B over 3 mins, hold at 0%B for 5 mins; Flow Rate: 15 mL/min; Injection volume: 200 µL; Sample concentration: 50 mg/mL; UV detection: 260 nm.

Yield: 2.3 mg (3.24 µmol, 22%); **^{1}H NMR** (400 MHz; D_2O) δ 8.03 (1 H, s, **H$_{8}$**), 6.24 (1 H, dd, $^{3}J_{H1'-F}$ = 17.9 Hz, $^{3}J_{H1'-H2'}$ = 2.3 Hz, **H$_{1'}$**), 5.69 (1 H, ddd, $^{3}J_{H1''-P\beta}$ = 8.1 Hz, $^{3}J_{H1''-F}$ = 5.9 Hz, $^{3}J_{H1''-H2''}$ = 2.1 Hz, **H$_{1''}$**), 5.47 (1 H, ddd, $^{2}J_{H2'-F}$ = 52.3 Hz, $^{3}J_{H2'-H3'}$ = 4.7 Hz, $^{3}J_{H2'-H1'}$ = 2.3 Hz, **H$_{2'}$**), 4.83 (1 H, ddd, $^{2}J_{H2''-F}$ = 49.4 Hz, $^{3}J_{H2''-H3''}$ = 2.4 Hz, $^{3}J_{H2''-H1''}$ = 1.8 Hz, **H$_{2''}$**), 4.82 (1 H, ddd, $^{3}J_{H3'-F}$ = 19.4, Hz, $^{3}J_{H3'-H4'}$ = 7.2 Hz, $^{3}J_{H3'-H2'}$ = 4.0 Hz, **H$_{3'}$**), 4.38 – 4.31 (2 H, m, **H$_{4'}$**, **H$_{5a'}$**), 4.24 (1 H, ddd, $^{2}J_{H5b'-H5a'}$ = 12.3 Hz, $^{3}J_{H5b'-P\alpha}$ = 6.2 Hz, $^{3}J_{H5b'-H4'}$ = 4.4 Hz, **H$_{5b'}$**), 3.98 (1 H, ddd, $^{3}J_{H3''-F}$ = 30.9 Hz, $^{3}J_{H3''-H4''}$ = 9.8 Hz, $^{3}J_{H3''-H2''}$ = 2.6 Hz, **H$_{3''}$**), 3.91 – 3.83 (2 H, m, **H$_{5''}$**, **H$_{6a''}$**), 3.81 – 3.69 (2 H, m, **H$_{4''}$**, **H$_{6b''}$**), 3.21 (6 H, q, ^{3}J = 7.3Hz, DN^{+}(**<u>CH$_2$</u>**CH$_3$)$_3$), 1.29 (9 H, t, ^{3}J = 7.3 Hz, DN^{+}(CH$_2$**<u>CH$_3$</u>**)$_3$); **^{13}C{^{1}H} NMR** (100 MHz; D_2O; from ^{1}H-^{13}C HSQC) δ 93.3 (**C$_{1''}$**), 93.2 (d, $^{1}J_{C2'-F}$ = 207.4 Hz, **C$_{2'}$**), 89.6 (d, $^{1}J_{C2''-F}$ = 173.3 Hz, **C$_{2''}$**), 86.5 (d, $^{2}J_{C1'-F}$ = 32.6 Hz, **C$_{1'}$**), 81.7 (**C$_{4'}$**), 73.4 (**C$_{5''}$**), 69.0 (d, $^{2}J_{C3''-F}$ = 17.6 Hz, **C$_{3''}$**), 68.3 (d, $^{2}J_{C3'-F}$ = 19.3 Hz, **C$_{3'}$**), 66.2 (**C$_{4''}$**), 64.1 (**C$_{5'}$**), 60.4 (**C$_{6''}$**), 46.5 (DN^{+}(**<u>CH$_2$</u>**CH$_3$)$_3$), 8.2 (DN^{+}(CH$_2$**<u>CH$_3$</u>**)$_3$); **^{19}F NMR** (377 MHz; D_2O) δ −203.87 (dt, $^{2}J_{F-H2'}$ = 51.9 Hz, $^{3}J_{F-H1'/H3'}$ = 18.1 Hz, 2′-F), −204.76 (ddd, $^{2}J_{F-H2''}$ = 49.3 Hz, $^{3}J_{F-H3''}$ = 31.4 Hz, $^{3}J_{F-H1''}$ = 6.2 Hz, 2″-F); **^{31}P NMR** (162 MHz; D_2O) δ −11.47 (d, $^{2}J_{P\alpha-P\beta}$ = 20.9 Hz, Pα), −14.01 (d, $^{2}J_{P\beta-P\alpha}$ = 20.7 Hz, Pβ); **HRMS** [ES-] found [M-H]$^{-}$ 608.0602, $C_{16}H_{23}F_2N_5O_{14}P_2$-H requires 608.0612, error −1.6 ppm;

Expression of *Pa*GMD

The recombinant plasmid (pET-3a) containing the *algD* gene encoding for GDP-mannose dehydrogenase (GMD) from *P. aeruginosa* was kindly donated by P. Tipton.[6]

The plasmid was transformed into *E. coli* soluBL21(DE3) chemically competent cells and the transformant grown according to the literature.[6,7] Briefly, 1 L of the transformant in LB medium containing the appropriate antibiotic (carbenicillin, 100 µg/mL) was incubated at 37 °C with gentle shaking in baffled flasks until an OD_{600} of 0.6–0.8 was reached. Heterologous protein expression was induced by adding isopropyl β-D-1-thiogalactopyranoside (IPTG) to a final concentration of 0.4 mM, followed by incubation at 37 °C for 4 hours at 180 rpm. Afterwards the cells were harvested by centrifugation (4000 *x g*, 4 °C, 20 mins) and stored at –80 °C until further purification.

Frozen cells were thawed in 20 mM HEPES (pH 7.5), 150 mM NaCl supplemented with DNase A (10 µg/mL, Sigma) then lysed by sonication (40% power, 2 secs on, 5 secs off, 30 cycles, 10 °C max). The supernatant was recovered by centrifugation (20,000 *x g*, 4 °C, 20 min) and nucleic acid precipitated through the addition of protamine sulfate (5 mg per gram wet cell pellet) and incubated on ice for 30 mins. Precipitated nucleic acid removed by centrifugation (20,000 *x g*, 4 °C, 20 min), the crude protein solution was fractionated with ammonium sulfate, with GMD precipitating between 40 and 60% saturation. Protein pellets were redissolved in 50 mM Tris (pH 7.8), 100 mM NaCl, 1 mM DTT and 0.5 mM NAD^+ and purified using an ÄKTA pure FPLC system (GE Healthcare) by gel filtration chromatography using a Cytiva Sephracyl S-200 16/600 column. Proteins were eluted with 50 mM Tris (pH 7.8), 100 mM NaCl, 1 mM DTT and 0.5 mM NAD^+ at the flow rate of 1 mL/min. GMD containing fractions were combined and concentrated to ~700 µM (concentration determined by nanodrop, ε_{280nm} = 27,390 M^{-1} cm^{-1}). Concentrated GMD was then divided into aliquots and stored at –80°C until required in 20% glycerol.

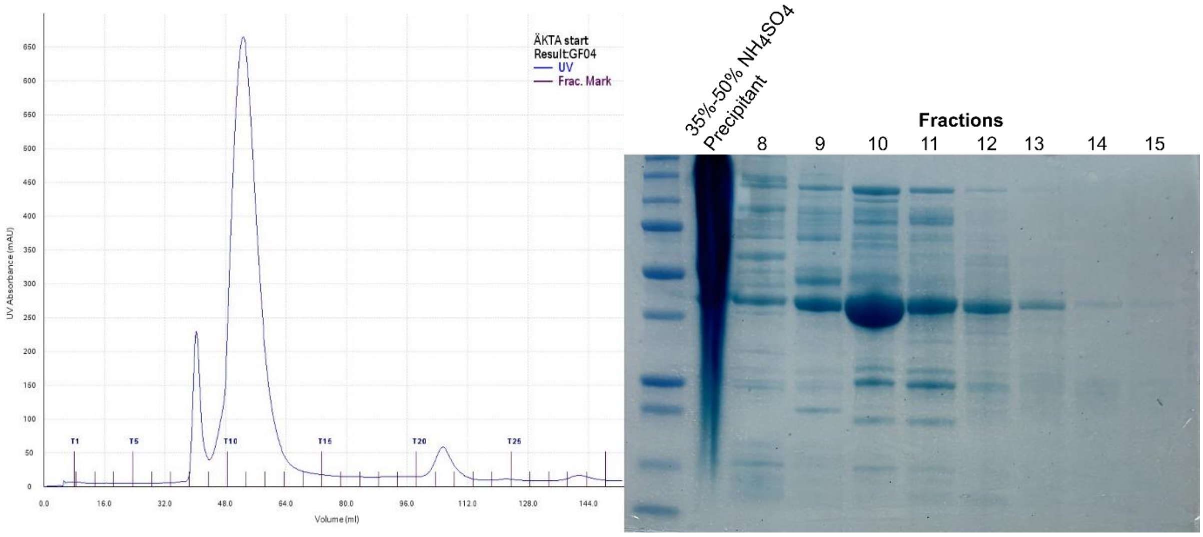

Figure S2: *Left) A_{280} trace for Sephracyl S-200 purification of 35%-50% sat. ammonium sulfate fractionation of GMD lysate. Right) SDS-PAGE gel of SEC fractions between Fractions 8 and 15, significantly purified GMD in Fraction 10.*

Kinetics Assay for GMD Activity

A 1:2 ratio was assumed in the oxidation of the substrates to the production of NADH. The rate of NADH production was detected by its absorbance at 340 nm (ε = 6300 M^{-1} cm^{-1}).[8] Sugar nucleotide substrates were dissolved in ultra-pure water to 100 mM then 2× stocks of each solution were prepared in 50 mM Na-Phosphate (pH 7.4), 0.5 mM $MgCl_2$, 1 mM DTT and 1 mM NAD^+. Typically, seven different substrate concentrations were prepared by 2-fold serial dilution from 500 µM down to 7.8 µM (2× stocks). GMD was diluted to 2 µM in 50 mM Na-Phosphate (pH 7.4), 0.5 mM $MgCl_2$, 1 mM DTT (2× stock).

The assay was performed in 96-well flat bottomed, non-binding, polystyrene microtiter plates (Grenier 655096). 100 µL of each substrate were added to a 96-well plate and the assay started by adding 100 µL of purified GMD (1 µM final). The absorbance at 340 nm was recorded for 1 hour on a TECAN Infinite M200 spectrophotometer at 25 °C and used to calculate initial rates.

The initial rate of fluorescence increase was calculated over the first 10 minutes. The absorbance was converted to [NADH] and rate of NADH production (slope) calculated. This data was then plotted against Sugar nucleotide concentration (M) for each sample using OriginPro 9.6 and the curve fitted using non-linear curve fit analysis using the Hill model (equation 1), where *x* is the Sugar nucleotide concentration (M), V_{max} is the maximum velocity, *k* is the Michaelis constant, and *n* is the number of cooperative sites. The errors on the V_{max} and K_M values represent the 95 % confidence interval based on the standard error of the regression.

$$y = V_{max}\frac{x^n}{k^n + x^n}$$

Equation S1: *Equation for non-linear curve fitting (Hill Equation).*

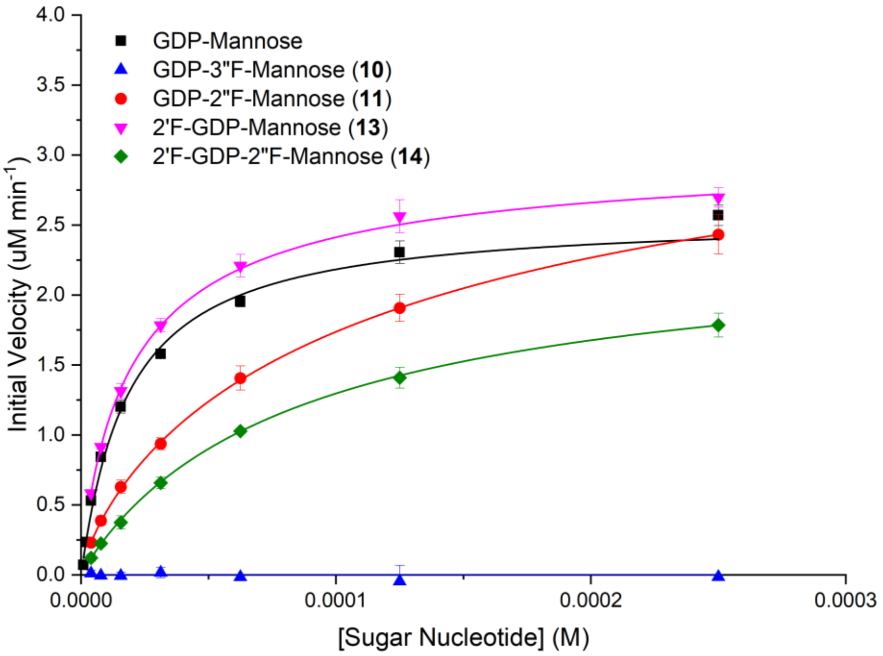

Figure S3: *Plot of average velocity verses substrate concentration for the oxidation of sugar nucleotides GDP-mannose,* ***10, 11, 13*** *&* ***14****. Data fitted using Hill model (OriginPro 9.6).*

Table S1: Kinetic parameters for GDP-mannose dehydrogenase with sugar nucleotides **GDP-mannose**, **10**, **11**, **13** & **14**.[a]

Entry	Substrate	V_{max} (μM min^{-1})	K_M (μM)	*Hill coefficient*	K_{cat} (min^{-1})	K_{cat}/K_M (min^{-1} μM^{-1})
**1**	GDP-Mannose	2.56 ± 0.20	18.18 ± 3.82	1.02 ± 0.07	2.56 ± 0.20	0.14 ± 0.05
**2**	GDP-3"F-Mannose (**10**)	_[b]	_[b]	_[b]	_[b]	_[b]
**3**	GDP-2"F-Mannose (**11**)	3.93 ± 0.16	134.29 ± 12.78	0.78 ± 0.01	3.93 ± 0.16	0.03 ± 0.01
**4**	2'F-GDP-Mannose (**13**)	3.04 ± 0.04	20.95 ± 0.83	0.86 ± 0.01	3.04 ± 0.04	0.15 ± 0.05
**5**	2'F-GDP-2"F-Mannose (**14**)	2.44 ± 0.07	87.72 ± 5.82	0.95 ± 0.02	2.44 ± 0.07	0.03 ± 0.01

[a]Assay Conditions: 25°C, 1 μM *Pa*GMD, 50 mM Na-Phosphate (pH 7.4), 0.5 mM $MgCl_2$, 1 mM DTT and 1 mM NAD^+. The increase in absorbance at 340 nm was followed. [b]No activity observed.

Molecular Docking

Ligands were modelled and optimized using LigPrep and MacroModel (2021-4, Schrödinger LLC, New York, NY) according to the OPLS (2021-4, Schrödinger LLC, New York, NY) forcefield parameters.[9] Crystallographic structure of *Pseudomonas aeruginosa* GMD in complex with GDP-mannuronic acid (PDB ID: 1MV8)[10] was downloaded from the RCSB Protein Data Bank (www.rcsb.org). Initial five SeGDP-Man-PP models were generated using AlphaFold3 (AF3) server;[11] protein sequence was obtained from NCBI server (https://www.ncbi.nlm.nih.gov) (GenBank: AFW04862.1), Mg^{2+} and guanosine 5′-triphosphate (GTP) were included. Protein-protein BLAST searches were also performed by NCBI server. The generated models were analysed using AF3 itself and SAVES webserver (v6.1) (https://saves.mbi.ucla.edu) and model0 was selected, accordingly. Protein structures were prepared for docking using the Protein Preparation Wizard of Maestro (2021-4, Schrödinger LLC, New York, NY). Centroid of the co-crystallized GDP-mannuronic acid (-42.74, 6.67, -21.96) for GMD and of GTP for SeGDP-Man-PP (-8.69, 1.10, -0.76) were defined as the centre for docking site set at a volume of 27.000 Å^3 and the grid maps were generated accordingly. Ligands were docked to the receptors using Glide (2021-4, Schrödinger LLC, New York, NY) at extra precision mode with 100 runs per ligand.[12] Free binding energy (ΔG) values were calculated for the obtained ligand-receptor complexes according to the MM-GBSA method using Maestro's Prime MM-GBSA panel (2021-4, Schrödinger LLC, New York, NY).[13] The poses were visually evaluated using Maestro.

Results and discussion

Predicted binding of 10 with GMD

Molecular docking predicted a pose for **10** that aligns well with both **14**'s predicted pose and the co-crystallized conformation of GDP-mannuronic acid in GMD active site with similar affinity as **14** (docking score: -15.8 and ΔG: -92.0 kcal/mol) (Figure S4). In this pose, fluorine substitution at C3" of mannose leads to cancellation of a water-mediated H bond with N214 backbone and a direct H bond with F158 backbone, both available with the C3"-OH of **14**.

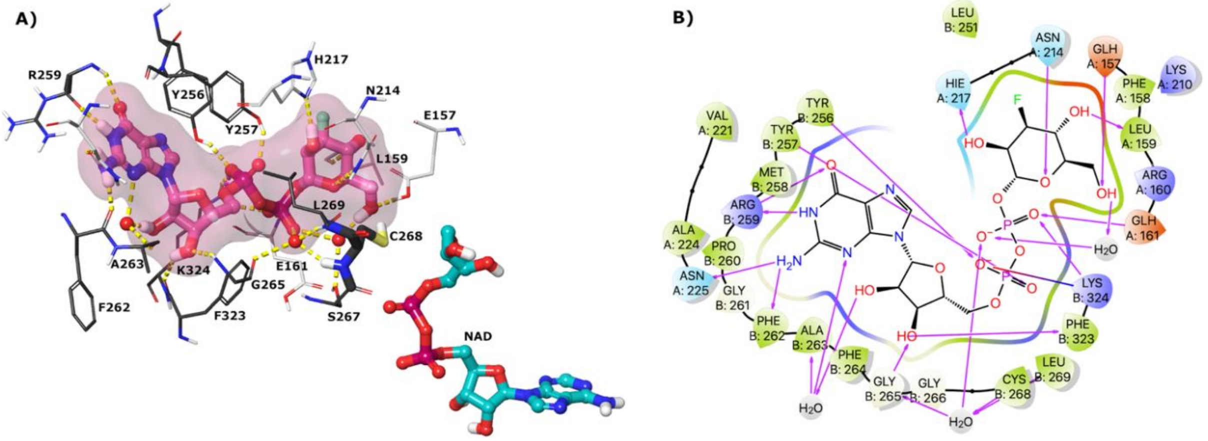

Figure S4: *Binding mode of **10** (salmon) in GMD active site predicted by molecular docking (Ligands are displayed in colour stick-and-ball representation, amino acid residues as thin tubes, except C268 in thick tubes, water molecules as red spheres, electrostatic interactions as colour dashed-lines. Molecular surface of **10** is rendered; chain A residues are highlighted in light grey, chain B residues in dark grey, and NAD(H) in teal). **B)** 2D interactions diagram for the predicted **10**-GMD complex.*

Modelling and docking with SeGDP-Man-PP

SeGDP-Man-PP was modelled to include substrate (GTP) and a Mg^{2+} ion in the active site since evidence indicates GDP-Man-PP are Mg^{2+}-dependent.[14–16] AF3 uses scores such as ipTM, pTM and plDDT to evaluate models. ipTM and pTM are derived from a metric called template modelling, which indicate the accuracy of the entire model. ipTM scores higher than 0.8 represent confident, high-quality predictions. Values over 0.5 for pTM mean the overall predicted fold for the complex might be close to the true structure. plDDT is a metric derived from the Local Distance Difference Test (lDDT), which evaluates local distance difference for each atom in a model independent from superposition.

AF3 metrics indicate high overall confidence (ipTM = 0.94 and pTM = 0.82) for the seGDP-Man-PP model, although low-confident regions, which are away from the active site, are present (Figure S5A). Most of the active site, which is located in the *N*-terminal domain, falls into very high-confident zones (plDDT > 90), while regions accommodating phosphate groups and Mg^{2+} are scored lower (90>plDDT > 70).[17] Stereochemical evolution was performed using Ramachandran plot,[18] a graph classifies each residue according to its compliance with the phi and psi angles, which labelled 89.8% of the residues in the most favoured area (cut-off is 90% for high-quality structures) (Figure S5B). As a result of these analyses, the AF3-generated SeGDP-Man-PP model is considered of reasonable quality for further modelling.

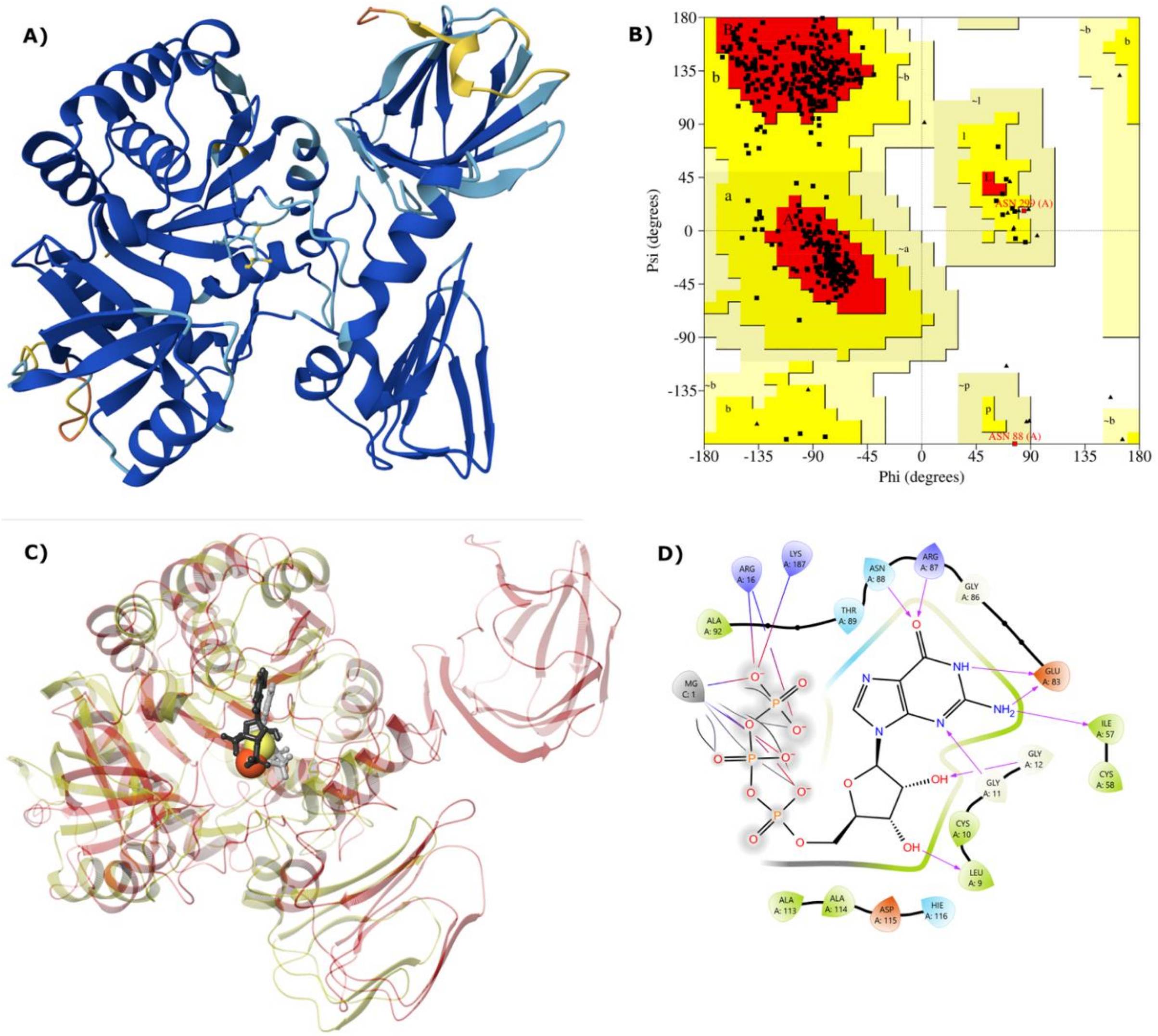

Figure S5: A) *AF-generated model of SeGDP-Man-PP in cartoon representation coloured according to pIDTT scores indicating confidence (Blue (very high): pIDTT>90, cyan (confident): 90>pIDTT>70, yellow (low): 70>pIDTT>50, orange (very low): pIDTT<50).* ***B)*** *Ramachandran plot of SeGDP-Man-PP model (red, yellow, light yellow and white indicates most favoured, additional allowed, generously allowed, and disallowed regions, respectively).* ***C)*** *Superposition of SeGDP-Man-PP (red cartoons) and TmGDP-Man-PP (yellow cartoons) according to C α atoms (GTPs are shown in black and grey stick-and-ball representation, Mg^{2+} ions in red and yellow CPK, respectively).* ***D)*** *Ligand-receptor interactions between GTP and SeGDP-Man-PP inferred from the AF3 model.*

Thermotoga maritima GDP-Man-PP (TmGDP-Man-PP) is the closest ortholog of SeGDP-Man-PP with a resolved crystallographic structure including a substrate. Amino acid sequence homology between the two is not high (identity: 32%), nevertheless superposition shows that TmGDP-Man-PP crystallographic structure and the AF3-generated of SeGDP-Man-PP have similar folding and active site architecture, and that GTP and Mg^{2+} from both orthologs align closely (Figure S5C). GTP in SeGDP-Man-PP was predicted with a wide array of H bonds and the phosphate backbone engaging strongly with Mg^{2+} (Figure S5D), similar as the co-crystallized GTP in TmGDP-Man-PP.[14] Likewise, molecular docking predicted a similar conformation for **13** (docking score: -15.9, ΔG: -27.4 kcal/mol) that aligns well with GTP. The

H bond array of the guanosine moiety includes contributions from L9, G11, I57, E83, R87 and N88 (Figure 3), which are observed with the guanosine of GTP. These residues correspond to I7, G9, V56, E80, K84 and N85 of TmGDP-Man-PP and make the same interactions with GTP.[14] The F substitution at C-2' of ribose of **13** cancels H bond with G12, which is normally seen with GTP-SeGDP-Man-PP complex, as well as between GTP and TeGDP-Man-PP (G12 of SeGDP-Man-PP corresponds to G10 of TeGDP-Man-PP). The phosphate backbone of **13** was predicted to engage with Mg^{2+} intensely like GTP and make an additional H bond with H116, not observed with GTP in SeGDP-Man-PP or TmGDP-Man-PP. Docking also predicted that the mannose of **13** was attached to the active site through H bonds with G155, E186, N205, and D261 (Figure S6), which are not present in the case of GTP as it does not have a mannose. Thus, it is currently not possible to vouch for the validity of these mannose interactions in the absence of experimental reference.

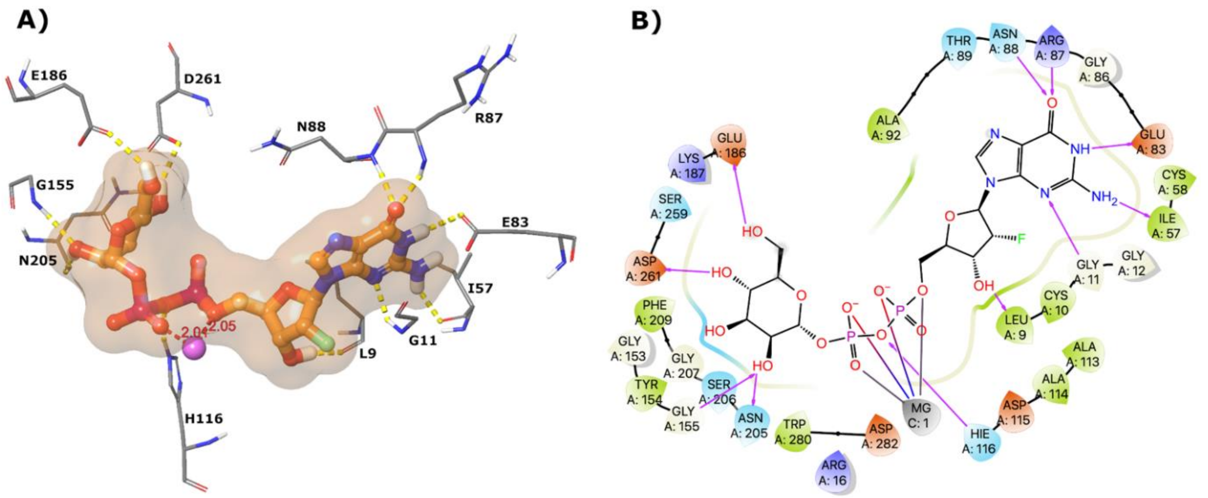

***Figure S6: A)** Binding mode of **13** (orange) in SeGDP-Man-PP active site predicted by molecular docking (Ligands are displayed in colour stick-and-ball representation, amino acid residues as thin tubes, water molecules as red spheres, and electrostatic interactions as colour dashed-lines. Molecular surface of **13** is rendered). **B)** 2D interactions diagram for the predicted **13**-SeGDP-Man-PP complex.*

References

1 L. Beswick, S. Ahmadipour, J. P. Dolan, M. Rejzek, R. A. Field and G. J. Miller, *Carbohydr Res*, 2019, **485**, 107819.

2 D. Crich and L. Li, *J Org Chem*, 2007, **72**, 1681–1690.

3 S. T. Evans, G. J. Tizzard, R. A. Field and G. J. Miller, *Carbohydr Res*, 2024, **545**, 109275.

4 J.-S. Zhu, N. E. McCormick, S. C. Timmons and D. L. Jakeman, *J Org Chem*, 2016, **81**, 8816–8825.

5 L. Zou, R. B. Zheng and T. L. Lowary, *Beilstein Journal of Organic Chemistry*, 2012, **8**, 1219–26.

6 L. E. Naught, S. Gilbert, R. Imhoff, C. Snook, L. Beamer and P. Tipton, *Biochemistry*, 2002, **41**, 9637–9645.

7 S. Ahmadipour, G. Pergolizzi, M. Rejzek, R. A. Field and G. J. Miller, *Org Lett*, 2019, **21**, 4415–4419.

8 R. B. McComb, L. W. Bond, R. W. Burnett, R. C. Keech and G. N. Bowers, *Clin Chem*, 1976, **22**, 141–50.

9 E. Harder, W. Damm, J. Maple, C. Wu, M. Reboul, J. Y. Xiang, L. Wang, D. Lupyan, M. K. Dahlgren, J. L. Knight, J. W. Kaus, D. S. Cerutti, G. Krilov, W. L. Jorgensen, R. Abel and R. A. Friesner, *J Chem Theory Comput*, 2016, **12**, 281–296.
10 C. F. Snook, P. A. Tipton and L. J. Beamer, *Biochemistry*, 2003, **42**, 4658–4668.
11 J. Jumper, R. Evans, A. Pritzel, T. Green, M. Figurnov, O. Ronneberger, K. Tunyasuvunakool, R. Bates, A. Žídek, A. Potapenko, A. Bridgland, C. Meyer, S. A. A. Kohl, A. J. Ballard, A. Cowie, B. Romera-Paredes, S. Nikolov, R. Jain, J. Adler, T. Back, S. Petersen, D. Reiman, E. Clancy, M. Zielinski, M. Steinegger, M. Pacholska, T. Berghammer, S. Bodenstein, D. Silver, O. Vinyals, A. W. Senior, K. Kavukcuoglu, P. Kohli and D. Hassabis, *Nature*, 2021, **596**, 583–589.
12 R. A. Friesner, R. B. Murphy, M. P. Repasky, L. L. Frye, J. R. Greenwood, T. A. Halgren, P. C. Sanschagrin and D. T. Mainz, *J Med Chem*, 2006, **49**, 6177–6196.
13 M. P. Jacobson, D. L. Pincus, C. S. Rapp, T. J. F. Day, B. Honig, D. E. Shaw and R. A. Friesner, *Proteins: Structure, Function, and Bioinformatics*, 2004, **55**, 351–367.
14 M.-C. Pelissier, S. A. Lesley, P. Kuhn and Y. Bourne, *Journal of Biological Chemistry*, 2010, **285**, 27468–27476.
15 M. D. Asención Diez, A. Demonte, J. Giacomelli, S. Garay, D. Rodrígues, B. Hofmann, H.-J. Hecht, S. A. Guerrero and A. A. Iglesias, *Arch Microbiol*, 2010, **192**, 103–114.
16 H. Hirayama and T. Suzuki, in *Handbook of Glycosyltransferases and Related Genes*, Springer Japan, Tokyo, 2014, pp. 1599–1606.
17 J. Abramson, J. Adler, J. Dunger, R. Evans, T. Green, A. Pritzel, O. Ronneberger, L. Willmore, A. J. Ballard, J. Bambrick, S. W. Bodenstein, D. A. Evans, C.-C. Hung, M. O'Neill, D. Reiman, K. Tunyasuvunakool, Z. Wu, A. Žemgulytė, E. Arvaniti, C. Beattie, O. Bertolli, A. Bridgland, A. Cherepanov, M. Congreve, A. I. Cowen-Rivers, A. Cowie, M. Figurnov, F. B. Fuchs, H. Gladman, R. Jain, Y. A. Khan, C. M. R. Low, K. Perlin, A. Potapenko, P. Savy, S. Singh, A. Stecula, A. Thillaisundaram, C. Tong, S. Yakneen, E. D. Zhong, M. Zielinski, A. Žídek, V. Bapst, P. Kohli, M. Jaderberg, D. Hassabis and J. M. Jumper, *Nature*, 2024, **630**, 493–500.
18 G. N. Ramachandran, C. Ramakrishnan and V. Sasisekharan, *J Mol Biol*, 1963, **7**, 95–99.

NMR Spectra

1,2,3,4,6-Penta-O-Acetyl-α-D-mannose (S2)

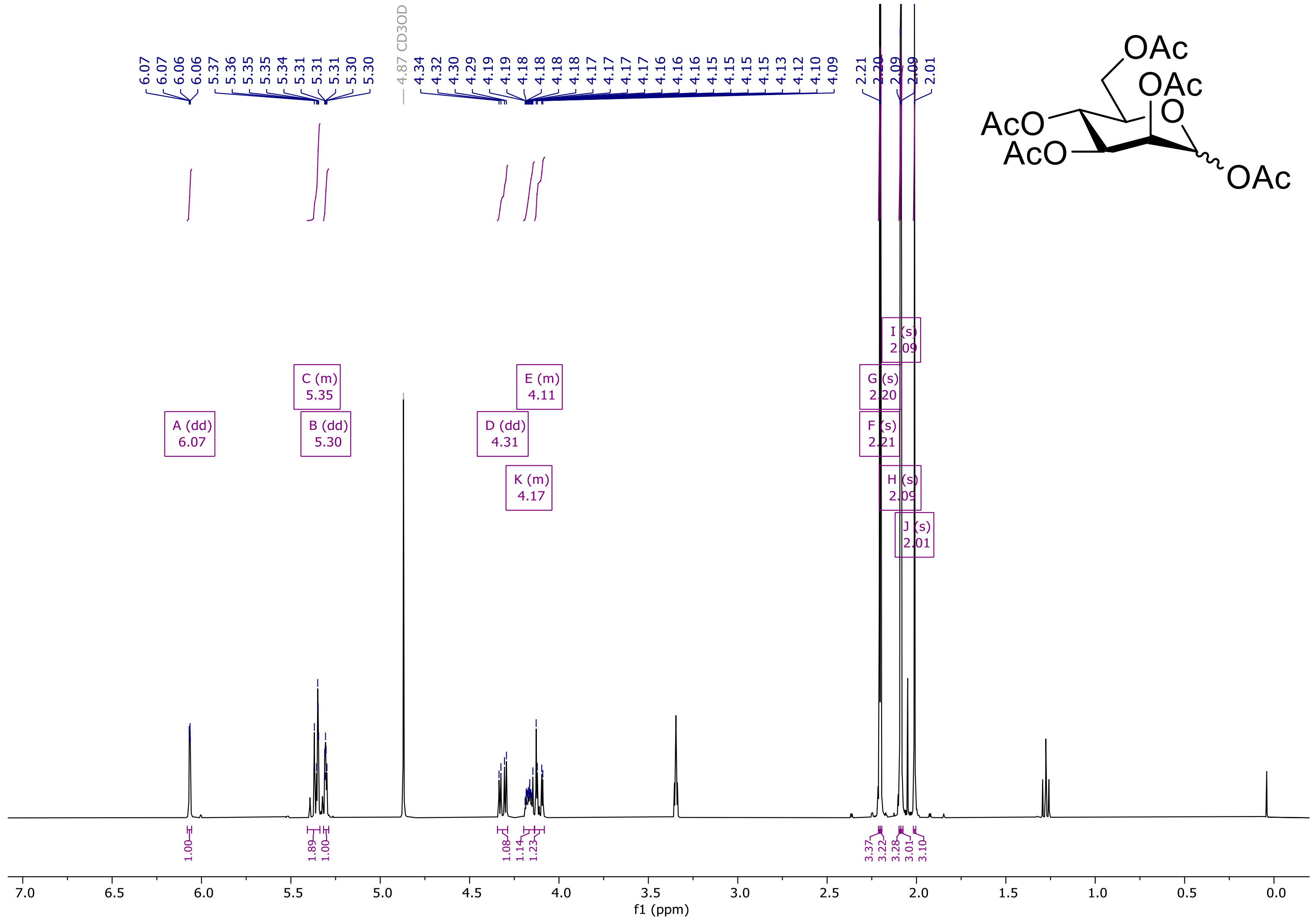

^1H NMR

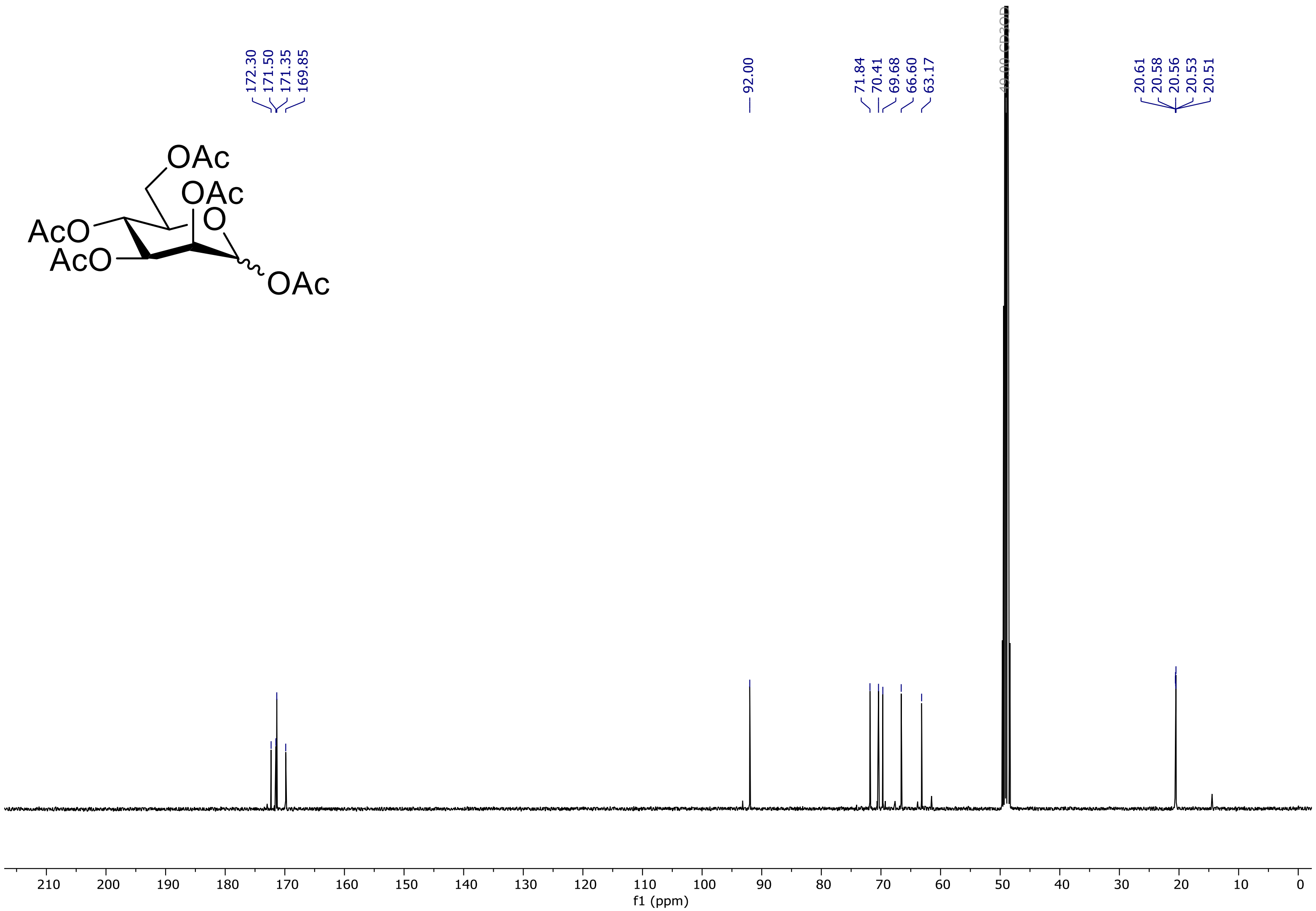
172.30
171.50
171.35
169.85
92.00
71.84
70.41
69.68
66.60
63.17
20.61
20.58
20.56
20.53
20.51
f1 (ppm)

13C NMR

α-D-Mannose-1-phosphate dilithium salt (S3)

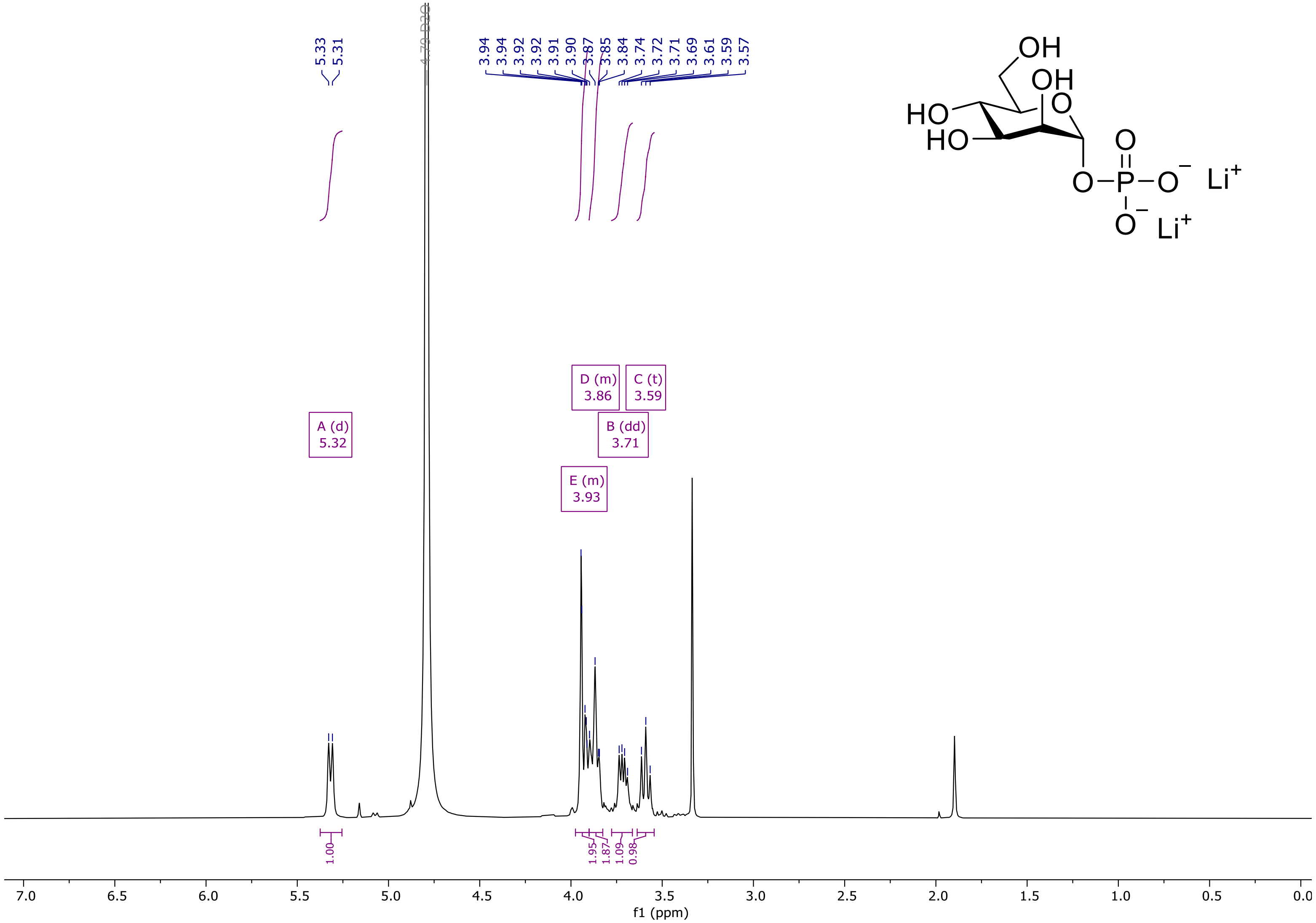

¹H NMR

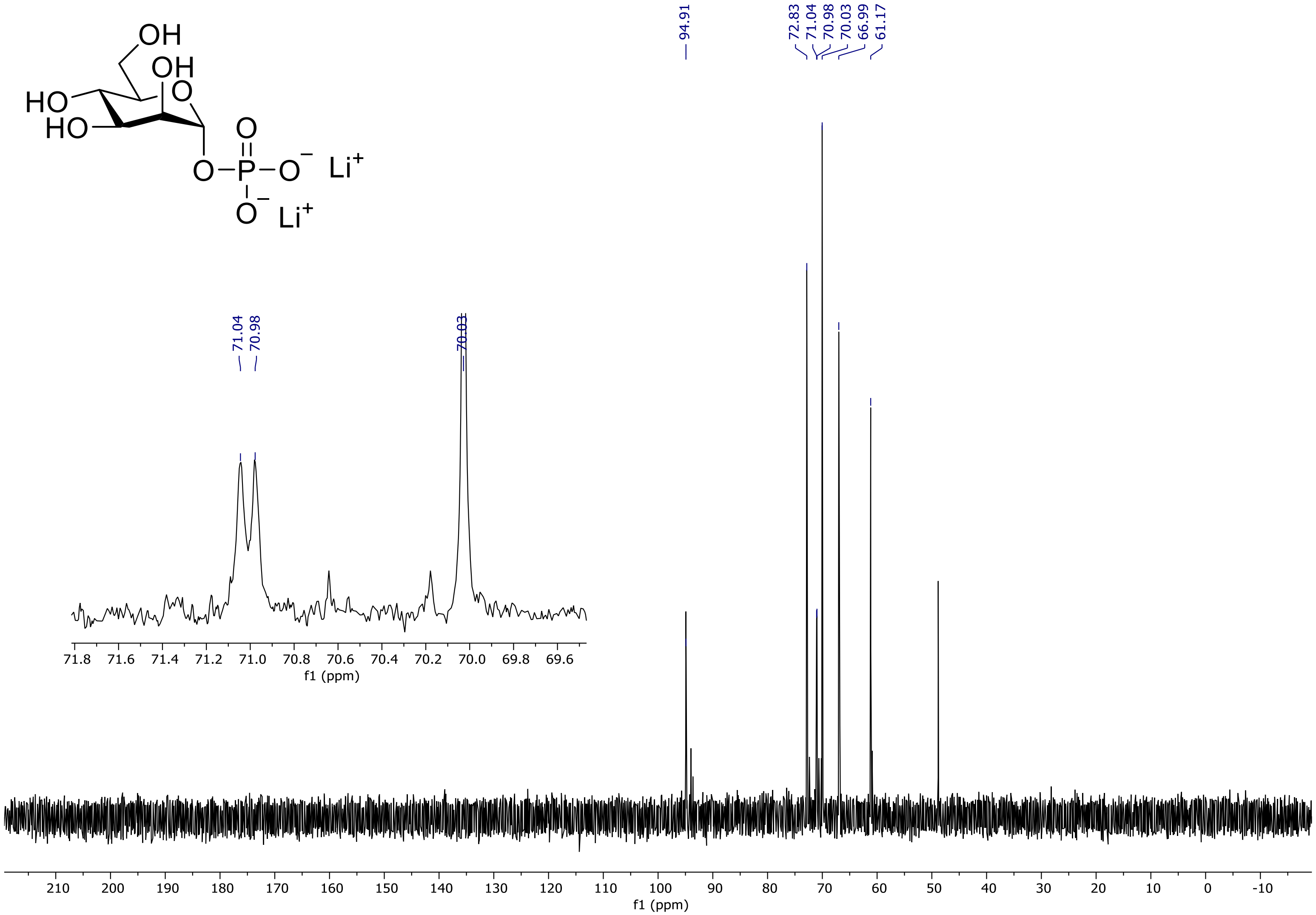

^{13}C NMR

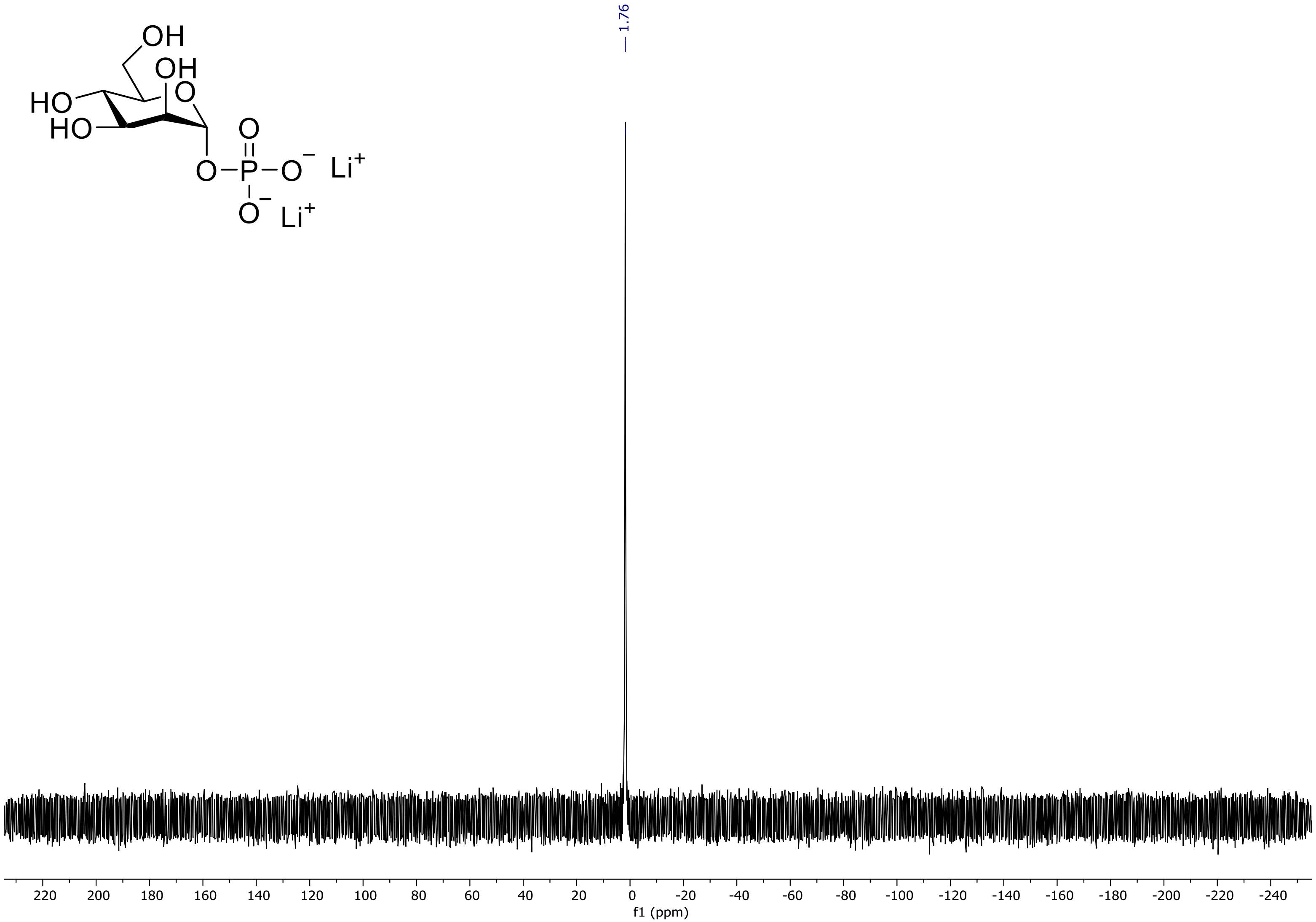
OH
OH
O
HO
HO
O
O
P
O⁻
O⁻
Li⁺
Li⁺
1.76
220 200 180 160 140 120 100 80 60 40 20 0 -20 -40 -60 -80 -100 -120 -140 -160 -180 -200 -220 -240
f1 (ppm)

^{31}P NMR

Phenyl 2-O-benzyl-3-deoxy-3-fluoro-1-thio-β-D-mannopyranoside (S4)

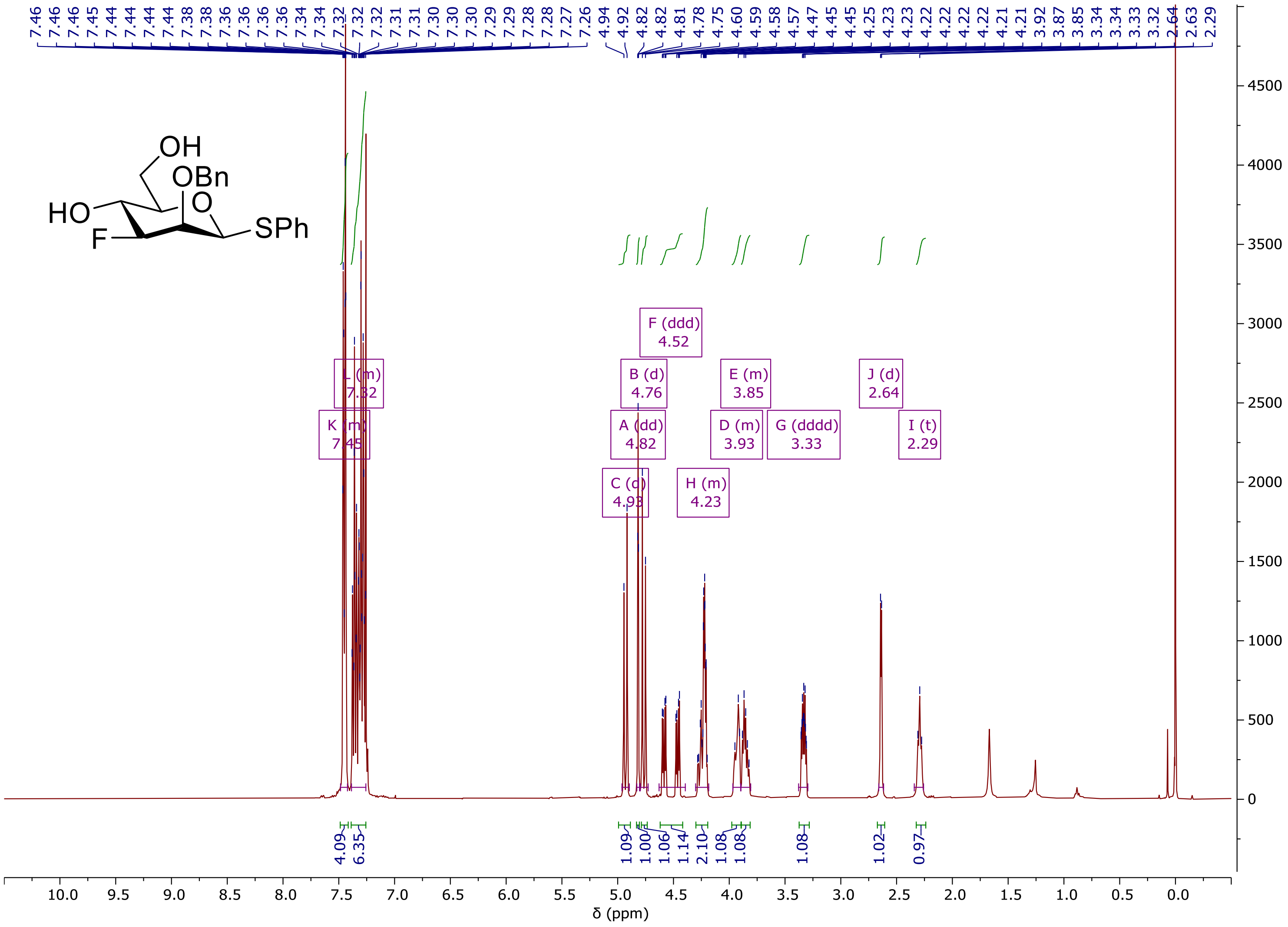

^{1}H NMR

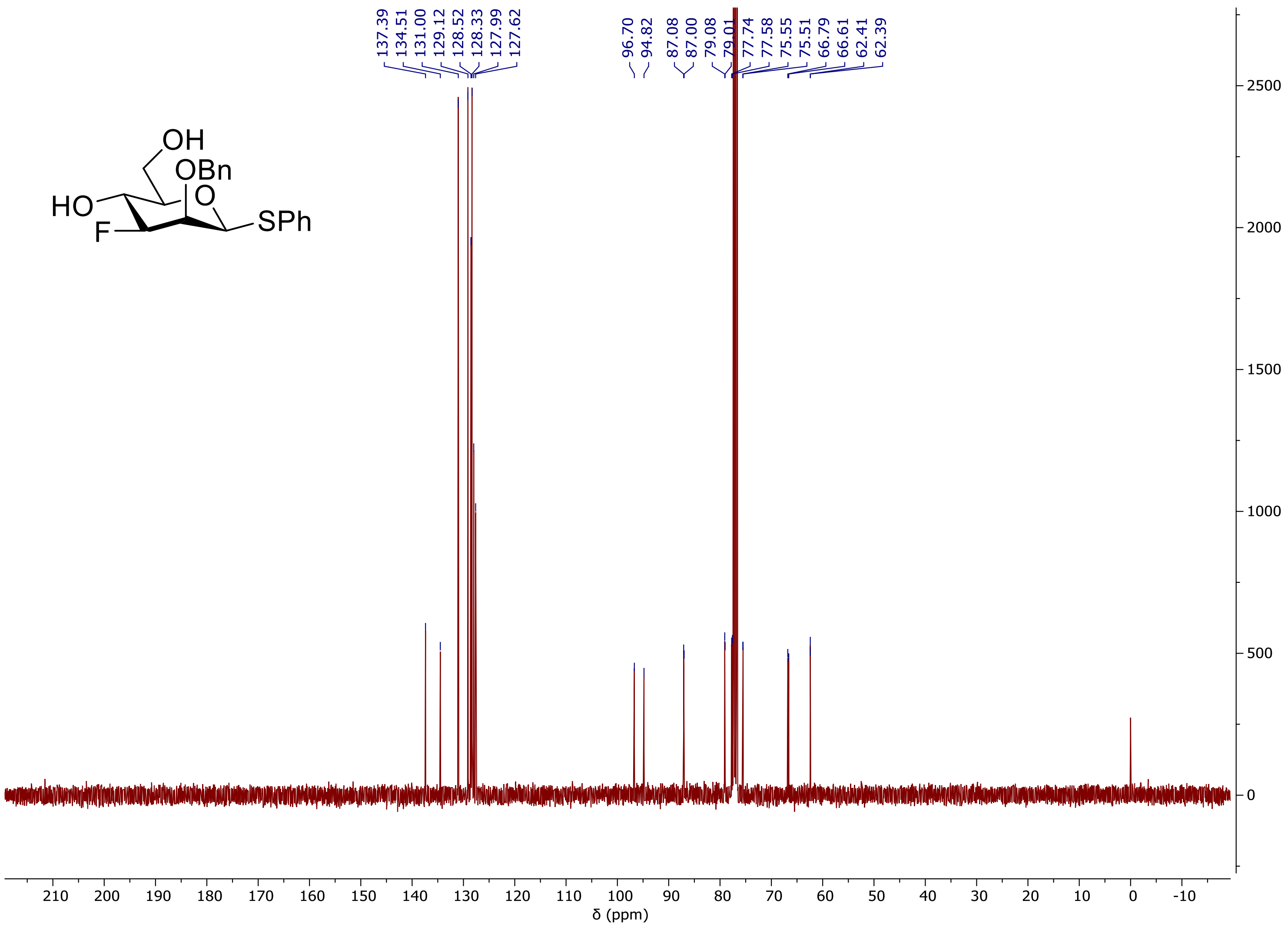
137.39
134.51
131.00
129.12
128.52
128.33
127.99
127.62
96.70
94.82
87.08
87.00
79.08
79.01
77.74
77.58
75.55
75.51
66.79
66.61
62.41
62.39
OH
OBn
HO
O
F
SPh
δ (ppm)

^{13}C NMR

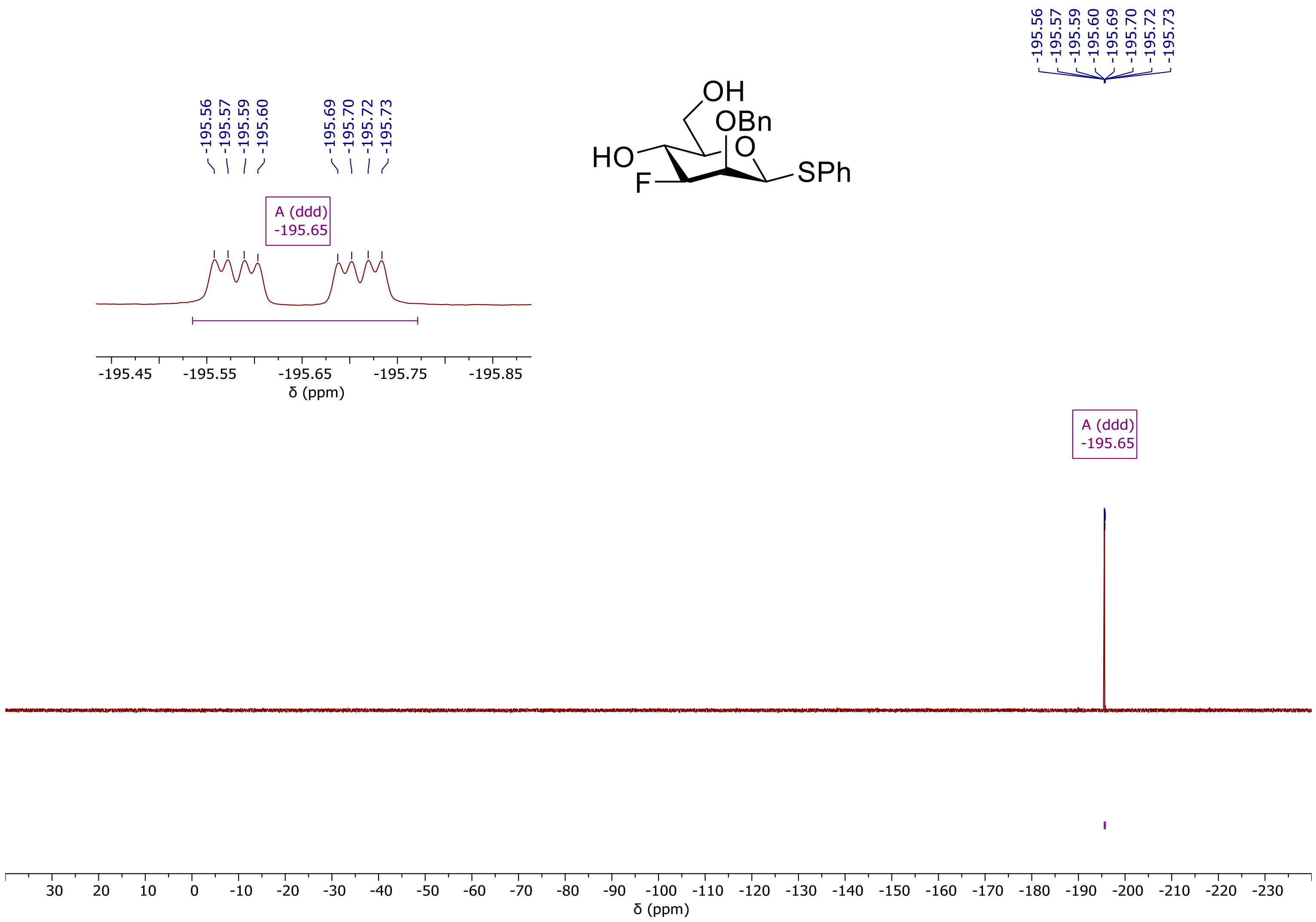
-195.56
-195.57
-195.59
-195.60
-195.69
-195.70
-195.72
-195.73
A (ddd)
-195.65
-195.45
-195.55
-195.65
-195.75
-195.85
δ (ppm)
OH
OBn
HO
O
SPh
F
-195.56
-195.57
-195.59
-195.60
-195.69
-195.70
-195.72
-195.73
A (ddd)
-195.65
30
20
10
0
-10
-20
-30
-40
-50
-60
-70
-80
-90
-100
-110
-120
-130
-140
-150
-160
-170
-180
-190
-200
-210
-220
-230
δ (ppm)

^{19}F NMR

Phenyl 2,4,6-tri-*O*-benzyl-3-deoxy-3-fluoro-1-thio-β-D-mannopyranoside (2)

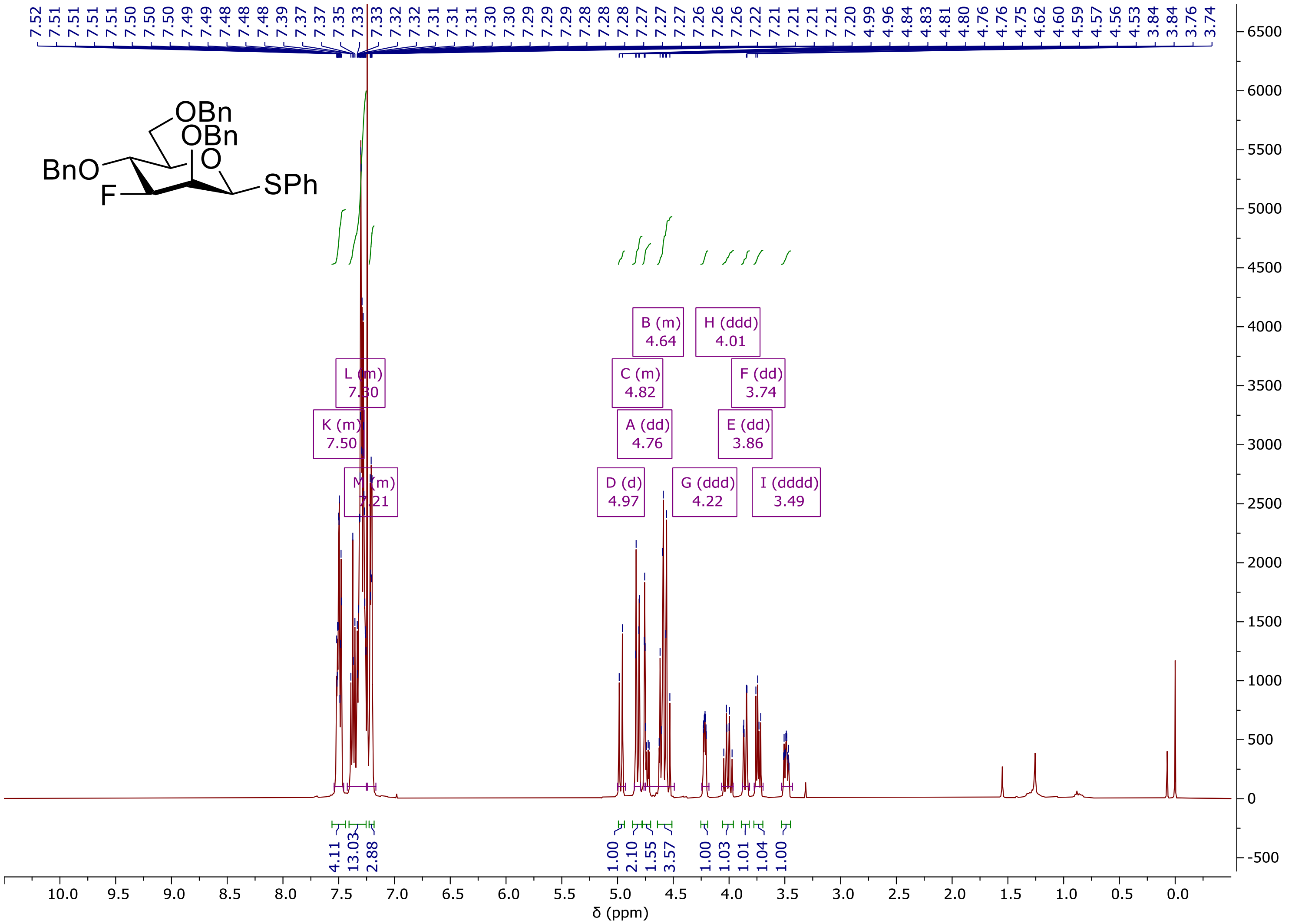

^{1}H NMR

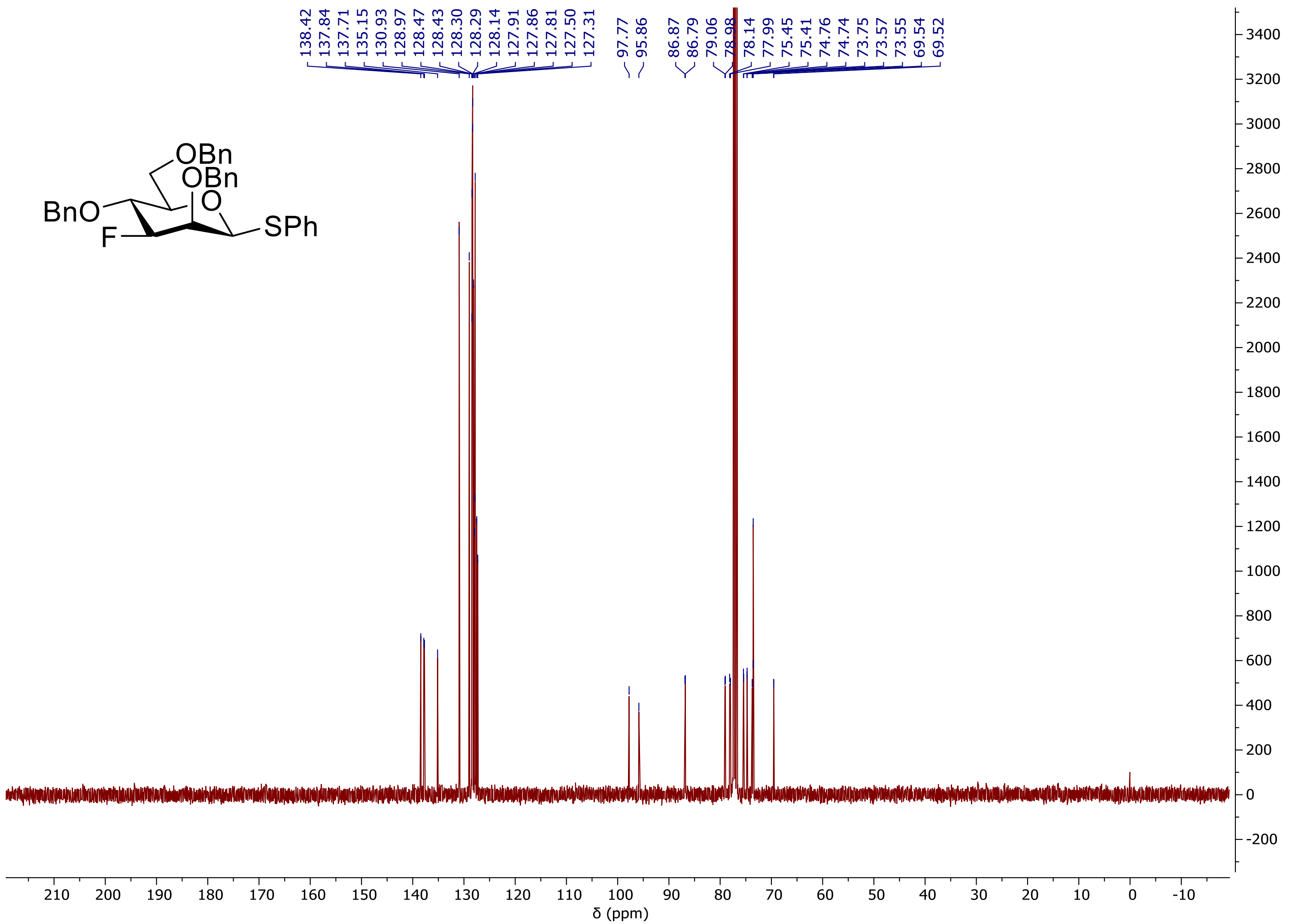
138.42
137.84
137.71
135.15
130.93
128.97
128.47
128.43
128.30
128.29
128.14
127.91
127.86
127.81
127.50
127.31
97.77
95.86
86.87
86.79
79.06
78.98
78.14
77.99
75.45
75.41
74.76
74.74
73.75
73.57
73.55
69.54
69.52
OBn
OBn
BnO
O
SPh
F
δ (ppm)

^{13}C NMR

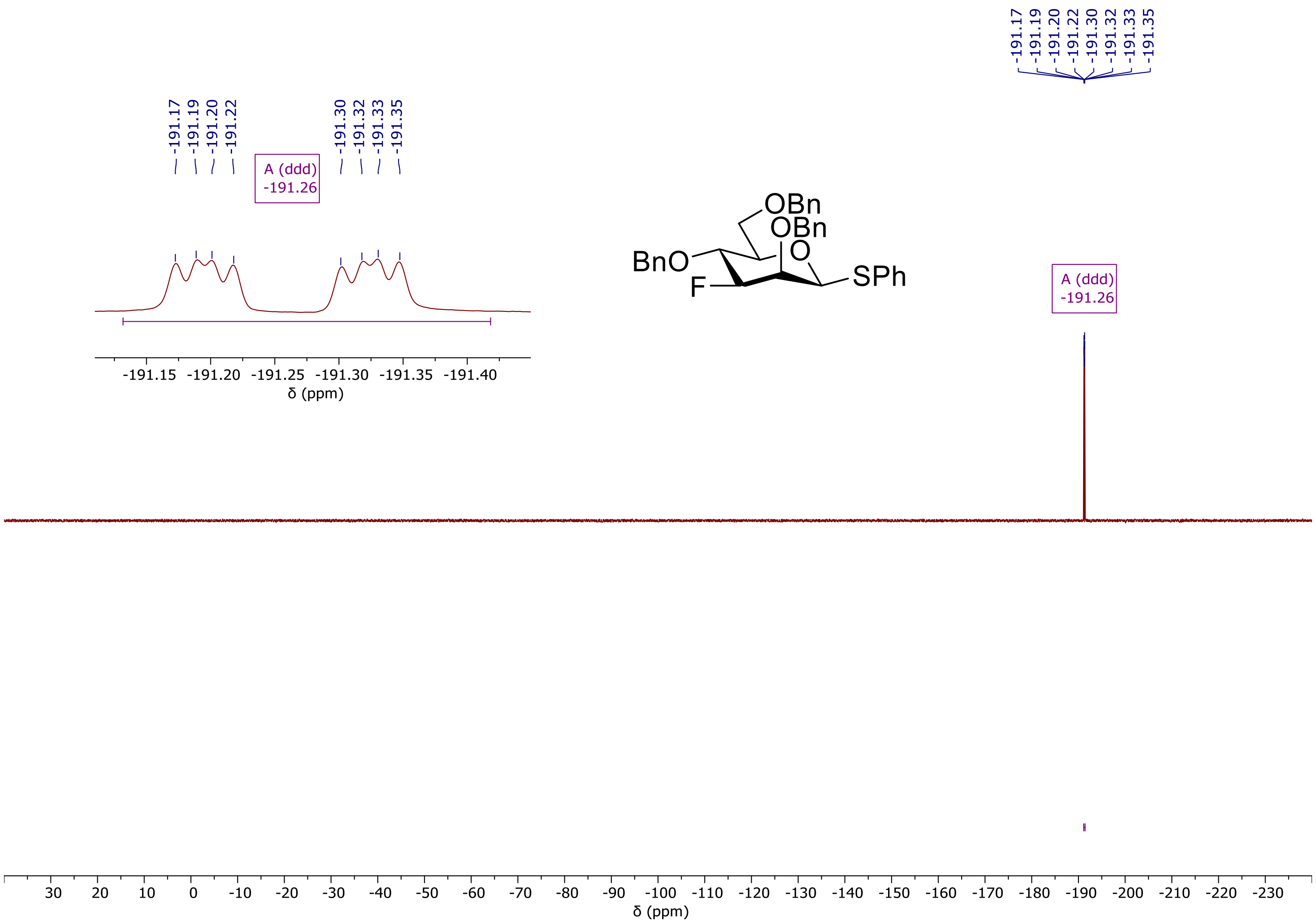

-191.17
-191.19
-191.20
-191.22
-191.30
-191.32
-191.33
-191.35
A (ddd)
-191.26
-191.15 -191.20 -191.25 -191.30 -191.35 -191.40
δ (ppm)
OBn
OBn
BnO
O
F
SPh
30 20 10 0 -10 -20 -30 -40 -50 -60 -70 -80 -90 -100 -110 -120 -130 -140 -150 -160 -170 -180 -190 -200 -210 -220 -230
δ (ppm)

^{19}F NMR

Di-*O*-benzyl (2,4,6-tri-O-benzyl-3-deoxy-3-fluoro)-1-phosphate-α-D-mannopyranoside (S5)

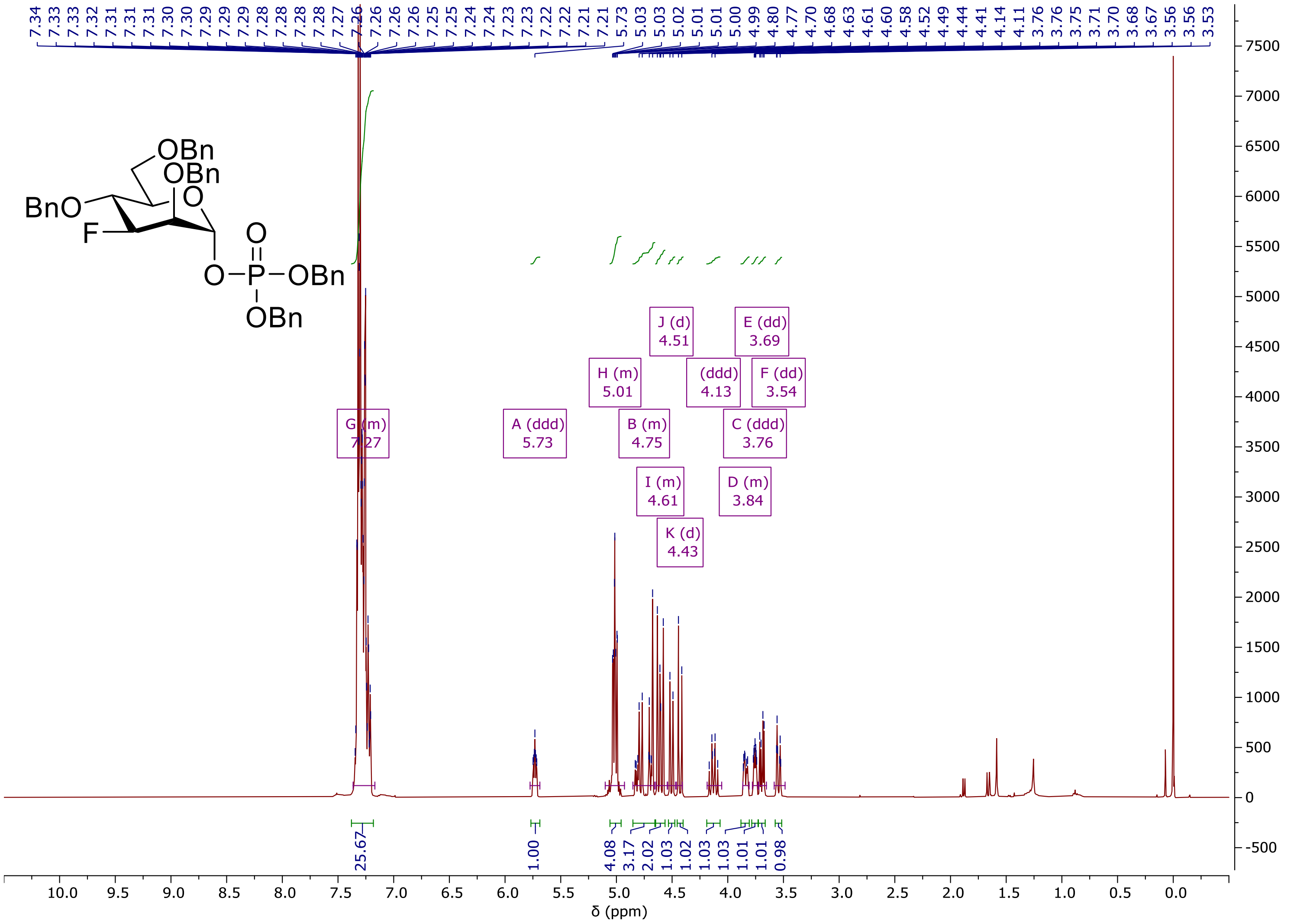

^{1}H NMR

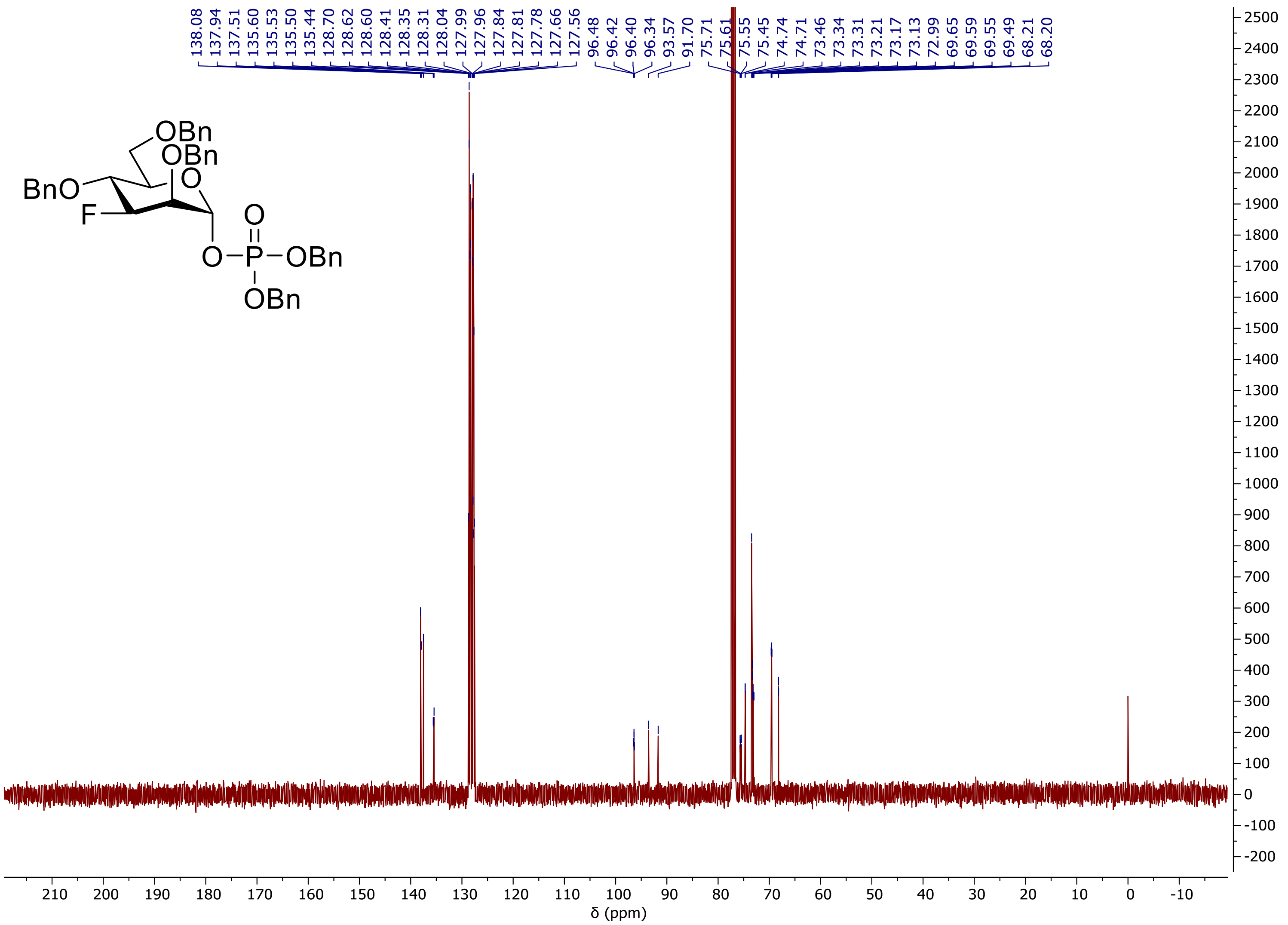
OBn
OBn
O
BnO
F
O
O–P–OBn
OBn
138.08 137.94 137.51 135.60 135.53 135.50 135.44 128.70 128.62 128.60 128.41 128.35 128.31 128.04 127.99 127.96 127.84 127.81 127.78 127.66 127.56 96.48 96.42 96.40 96.34 93.57 91.70 75.71 75.61 75.55 75.45 74.74 74.71 73.46 73.34 73.31 73.21 73.17 73.13 72.99 69.65 69.59 69.55 69.49 68.21 68.20
δ (ppm)

^{13}C NMR

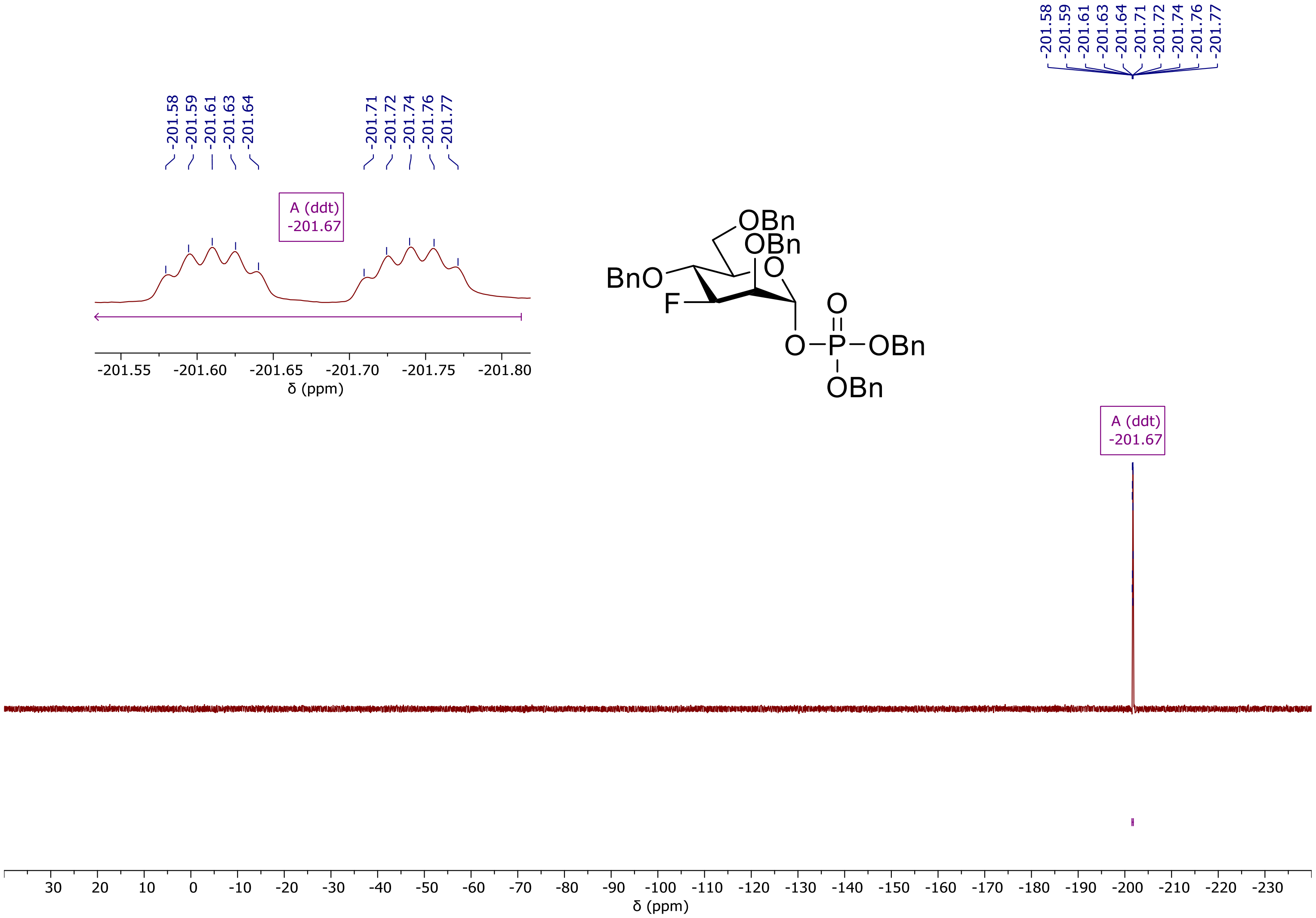
-201.58
-201.59
-201.61
-201.63
-201.64
-201.71
-201.72
-201.74
-201.76
-201.77
A (ddt)
-201.67
-201.55
-201.60
-201.65
-201.70
-201.75
-201.80
δ (ppm)
OBn
OBn
BnO
O
F
O
O
P
OBn
OBn
30 20 10 0 -10 -20 -30 -40 -50 -60 -70 -80 -90 -100 -110 -120 -130 -140 -150 -160 -170 -180 -190 -200 -210 -220 -230
δ (ppm)

^{19}F NMR

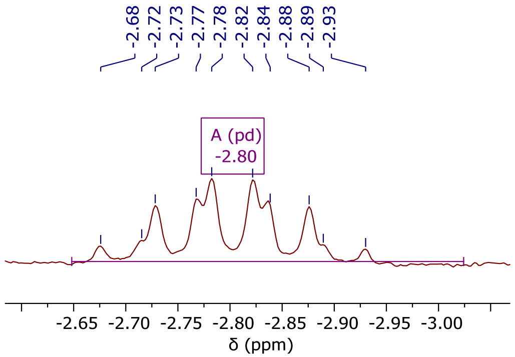
-2.68
-2.72
-2.73
-2.77
-2.78
-2.82
-2.84
-2.88
-2.89
-2.93
A (pd)
-2.80
-2.65 -2.70 -2.75 -2.80 -2.85 -2.90 -2.95 -3.00
δ (ppm)

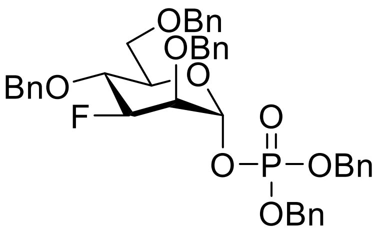
OBn
OBn
BnO
O
F
O
O
P
OBn
OBn

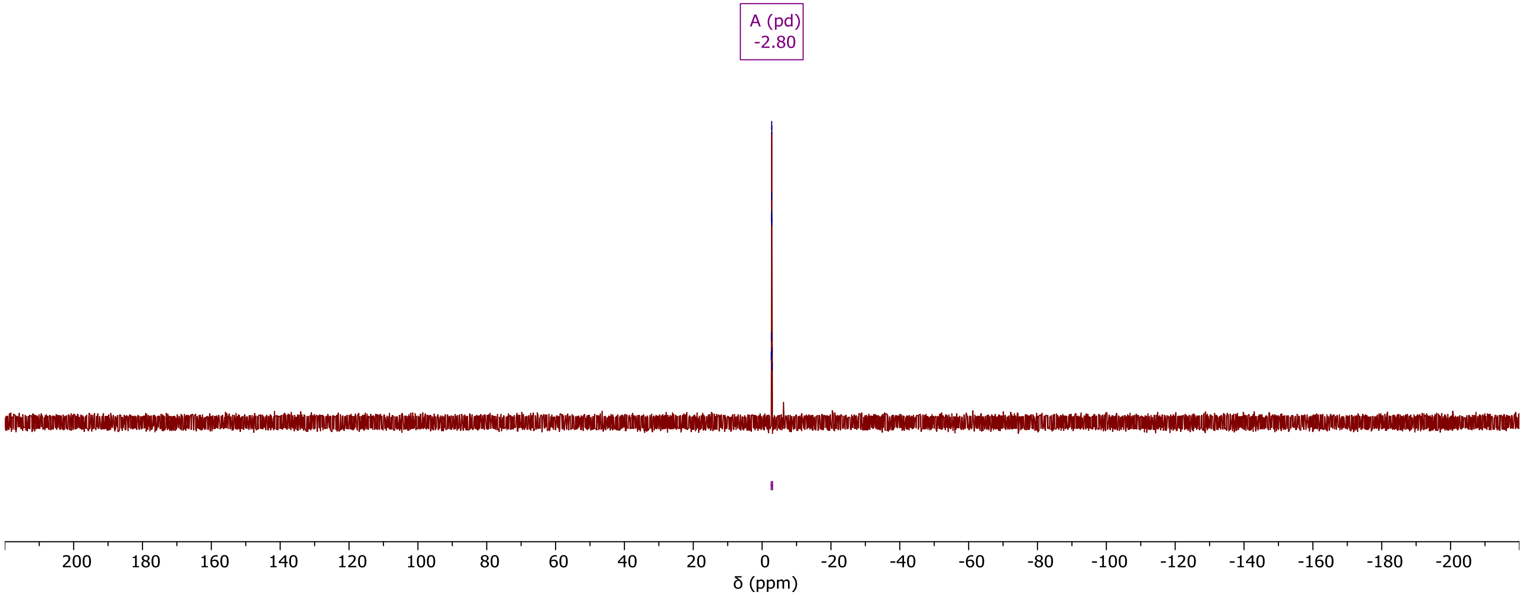
-2.68
-2.72
-2.73
-2.77
-2.78
-2.82
-2.84
-2.88
-2.89
-2.93
A (pd)
-2.80
200 180 160 140 120 100 80 60 40 20 0 -20 -40 -60 -80 -100 -120 -140 -160 -180 -200
δ (ppm)

^{31}P NMR

3-Deoxy-3-fluoro-α-D-mannose-1-phosphate (bis ammonium salt) (3)

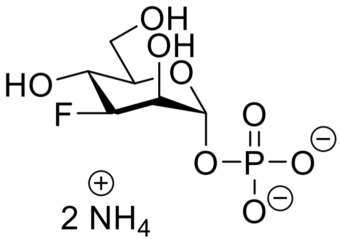

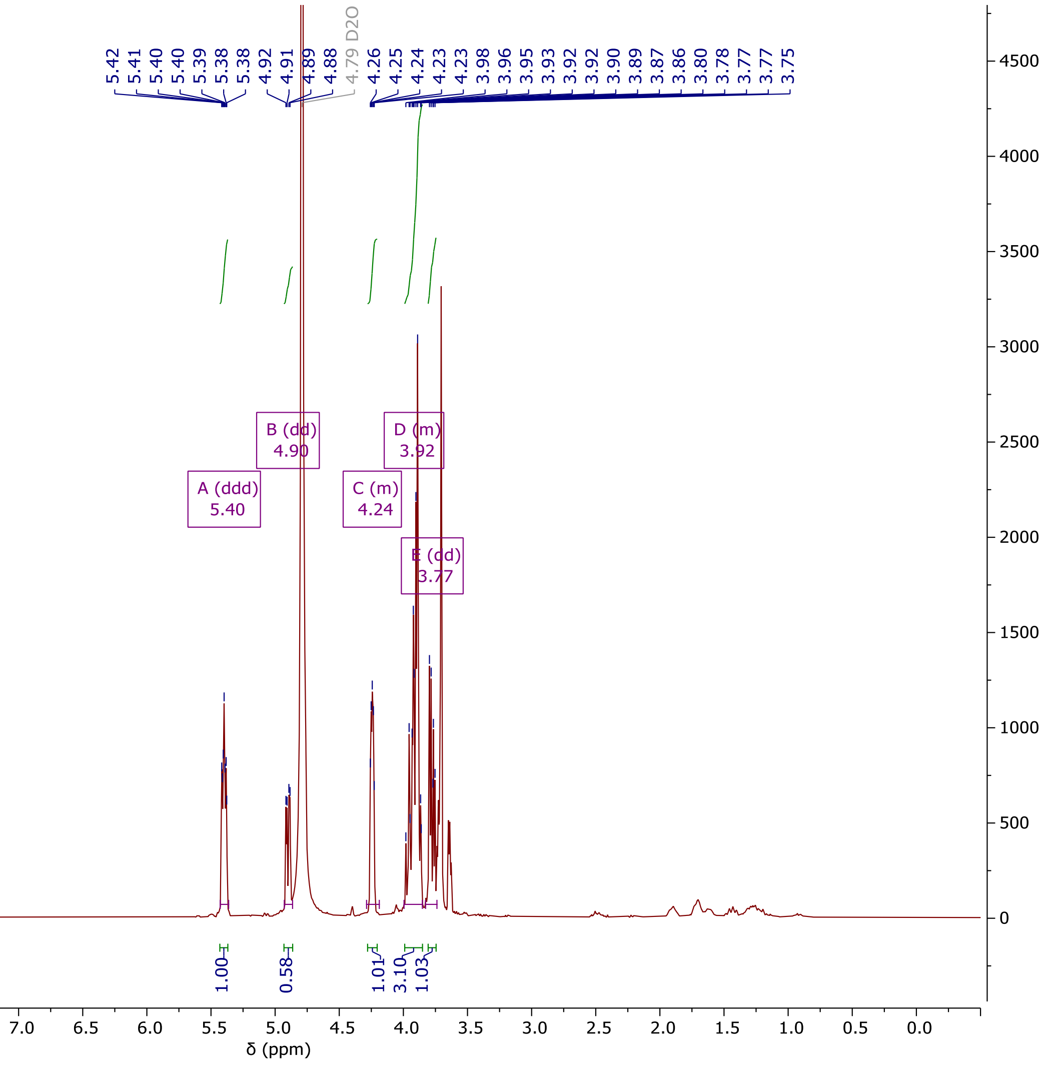

^{1}H NMR

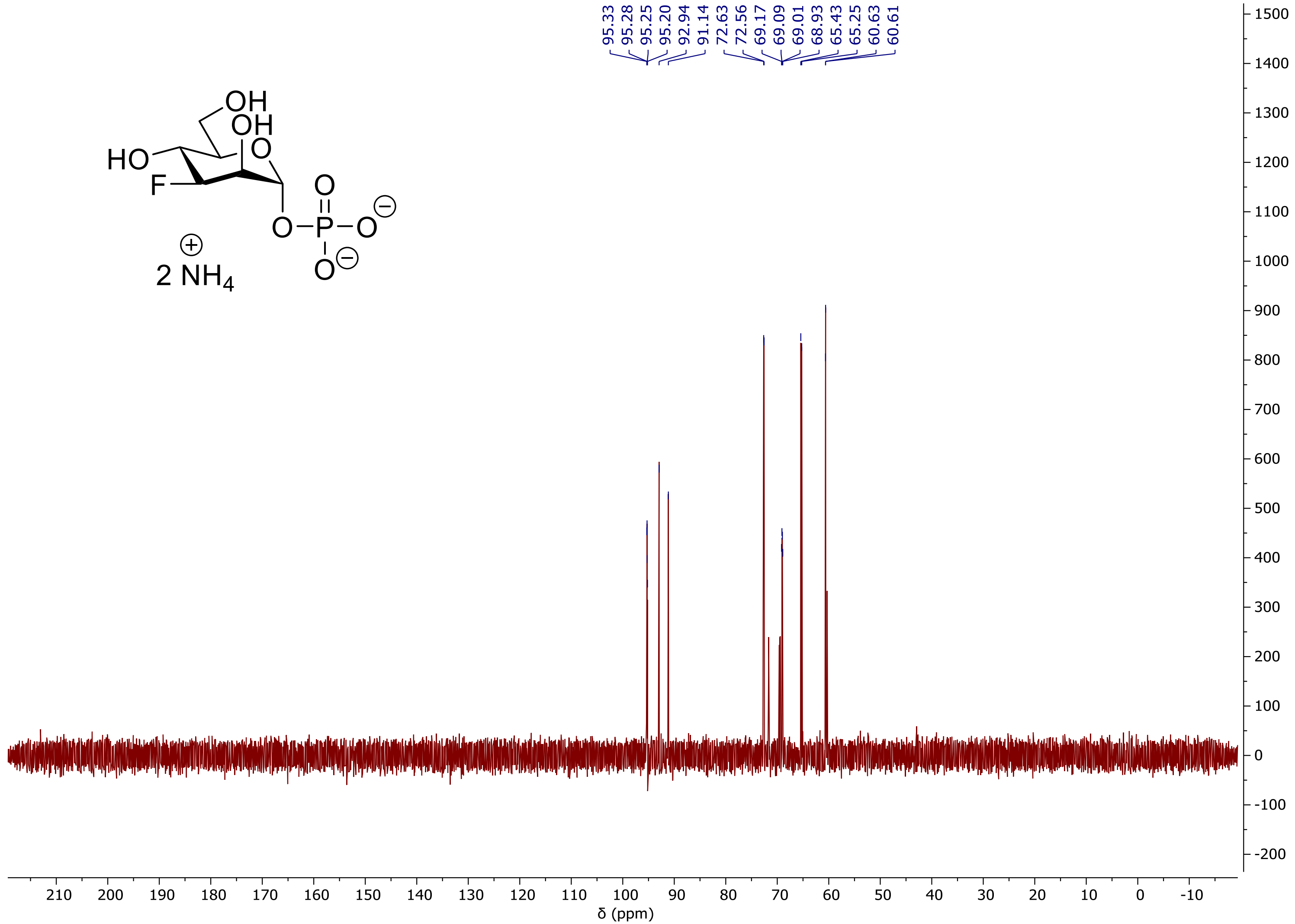

95.33
95.28
95.25
95.20
92.94
91.14
72.63
72.56
69.17
69.09
69.01
68.93
65.43
65.25
60.63
60.61
OH
OH
HO
O
F
O
O
P
O
O
2 NH4
δ (ppm)

^{13}C NMR

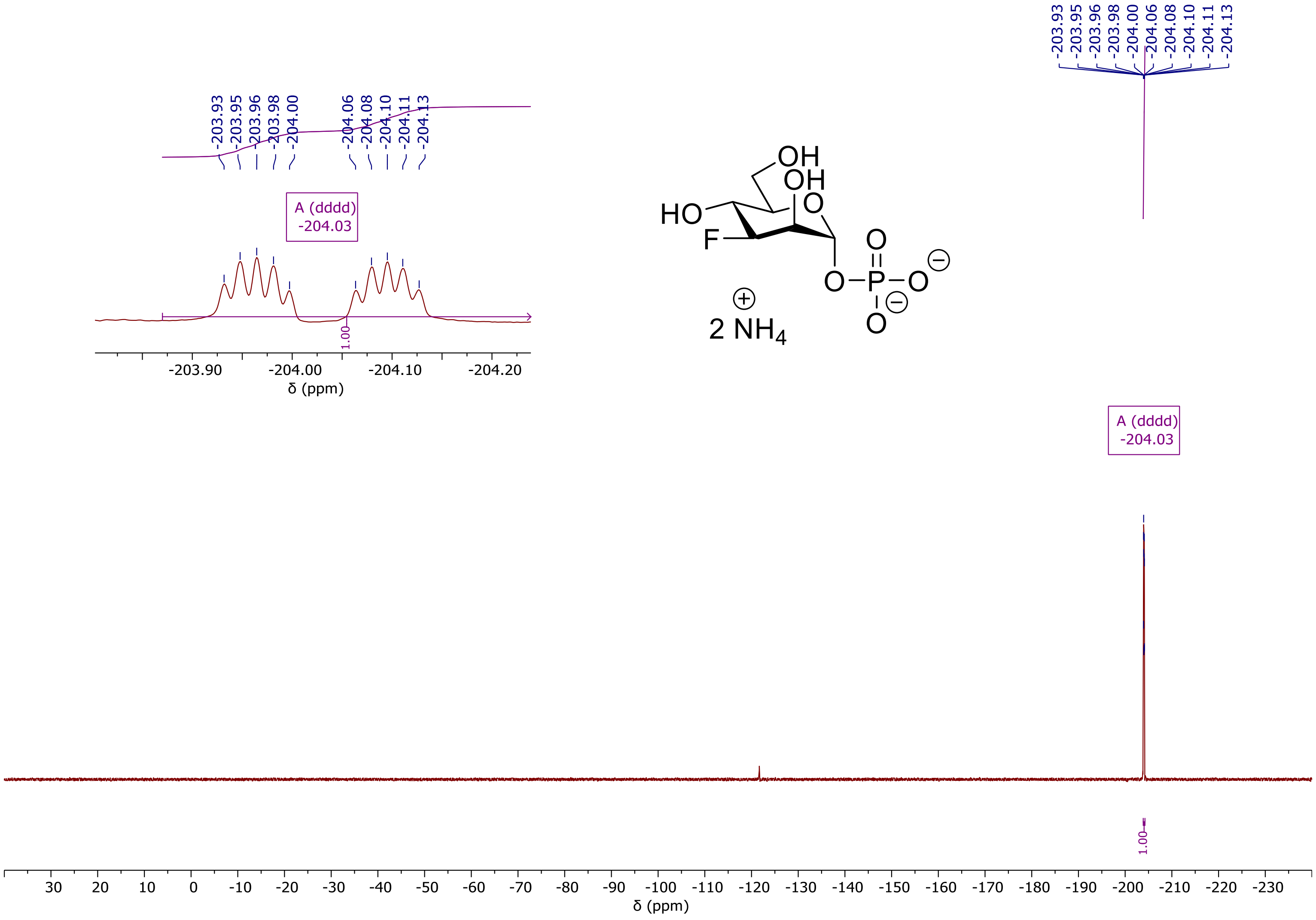
-203.93
-203.95
-203.96
-203.98
-204.00
-204.06
-204.08
-204.10
-204.11
-204.13
A (dddd)
-204.03
1.00
-203.90
-204.00
-204.10
-204.20
δ (ppm)
OH
OH
HO
O
F
O
O
P
O
O
2 NH4
30
20
10
0
-10
-20
-30
-40
-50
-60
-70
-80
-90
-100
-110
-120
-130
-140
-150
-160
-170
-180
-190
-200
-210
-220
-230

^{19}F NMR

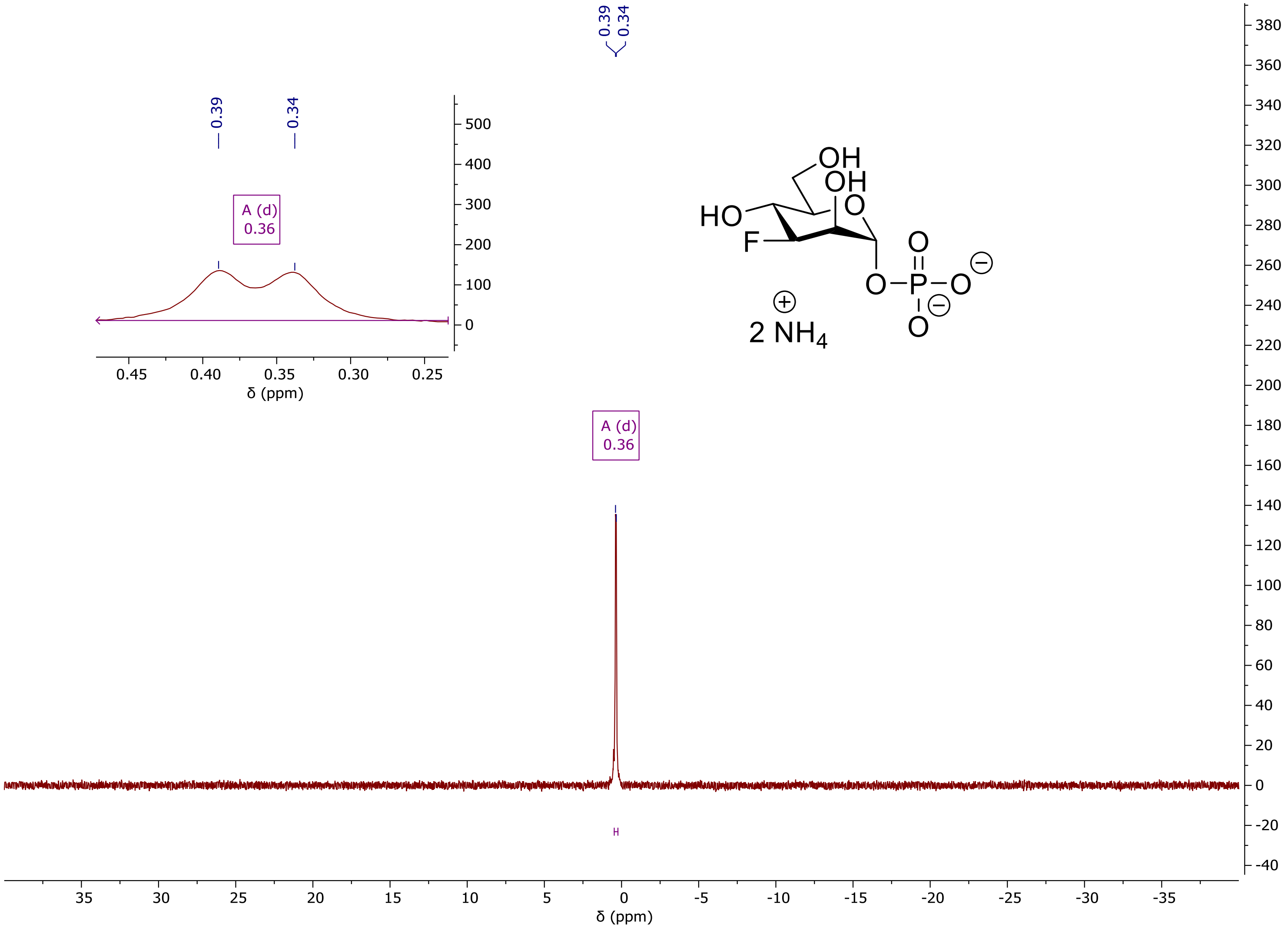

31P NMR

p-Methoxyphenyl 3-*O*-benzyl-2-deoxy-2-fluoro-β-D-mannopyranoside (S6)

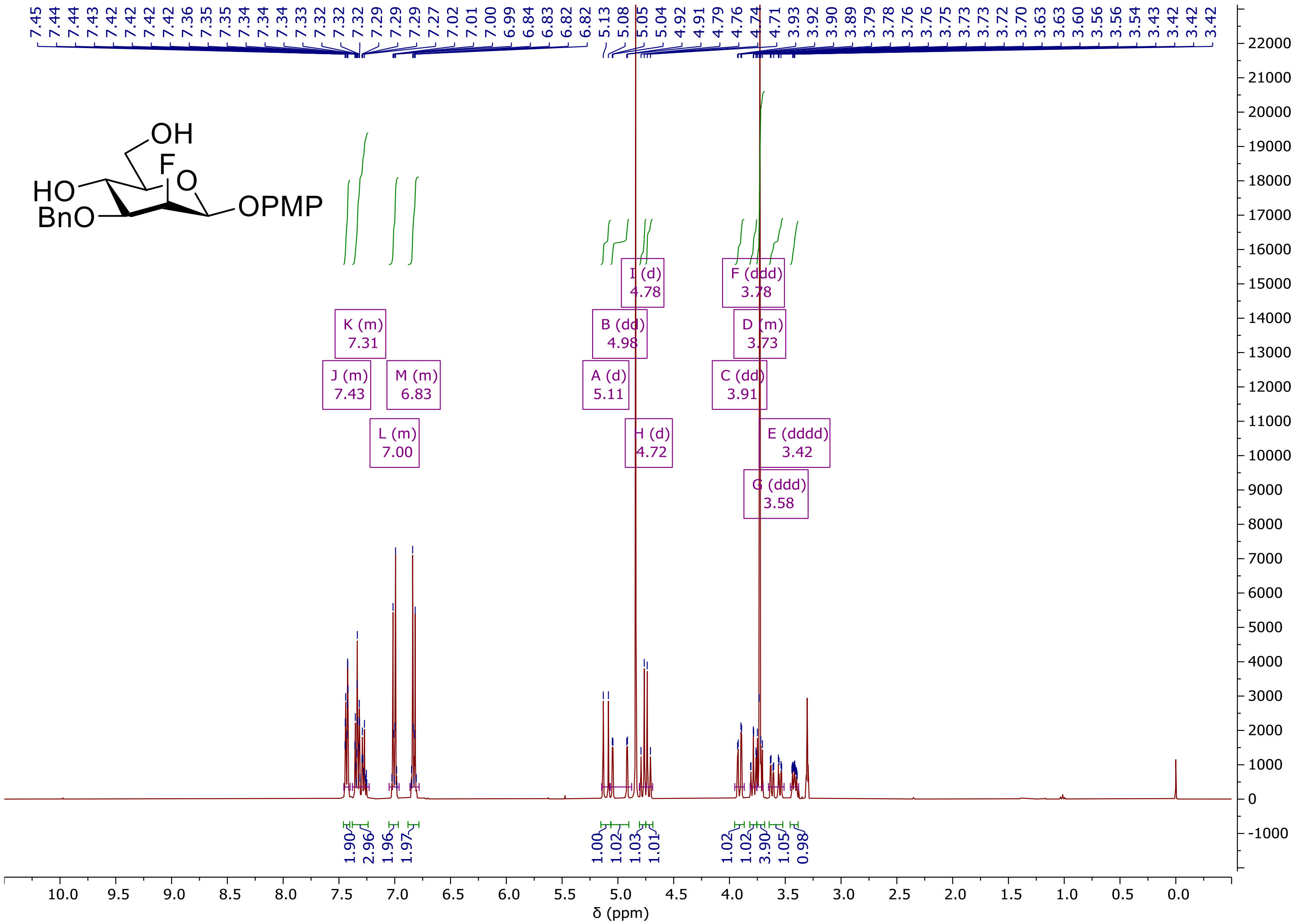

^{1}H NMR

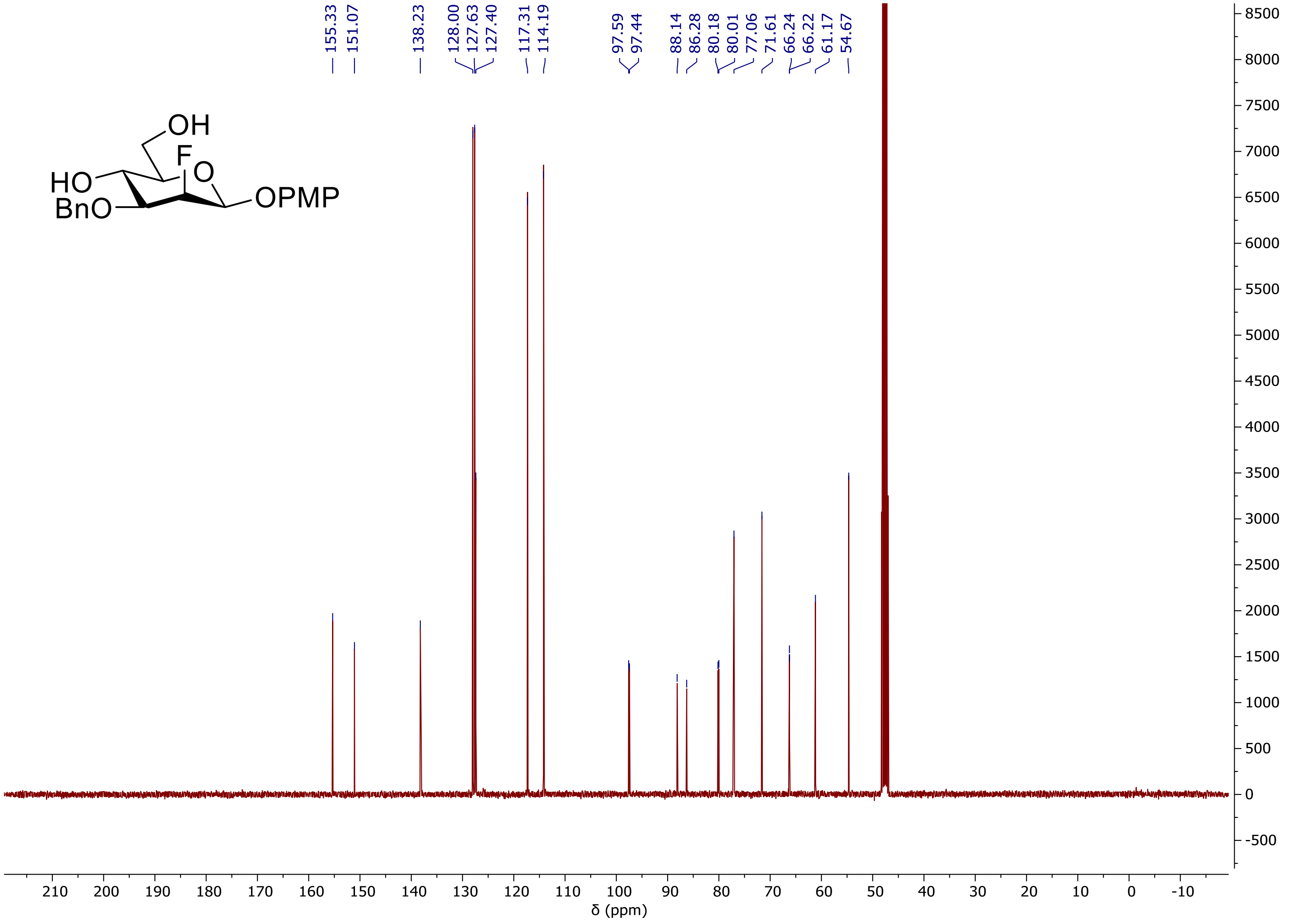

155.33
151.07
138.23
128.00
127.63
127.40
117.31
114.19
97.59
97.44
88.14
86.28
80.18
80.01
77.06
71.61
66.24
66.22
61.17
54.67
OH
F
O
HO
BnO
OPMP
δ (ppm)

^{13}C NMR

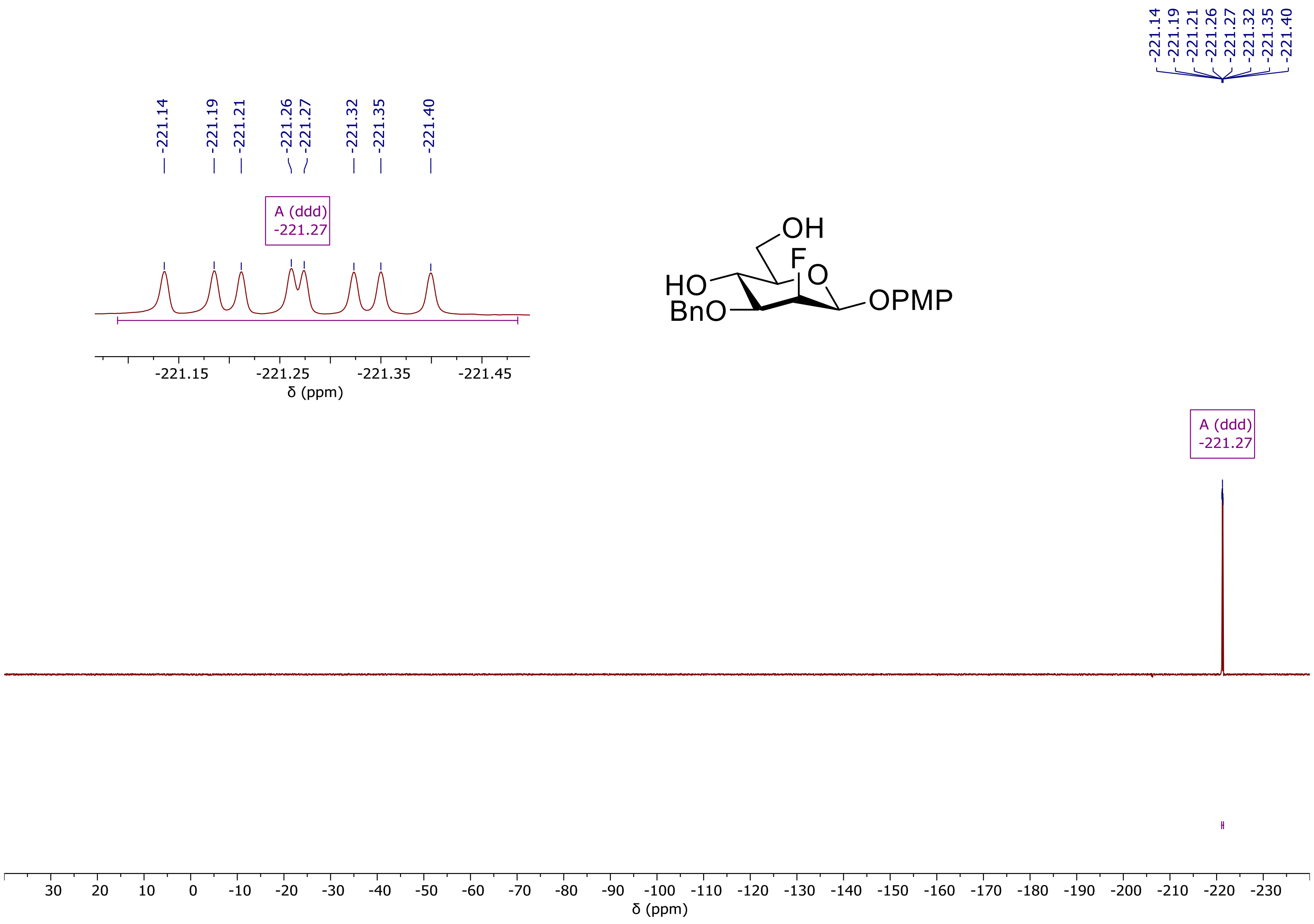
-221.14
-221.19
-221.21
-221.26
-221.27
-221.32
-221.35
-221.40
A (ddd)
-221.27
-221.15
-221.25
-221.35
-221.45
δ (ppm)
OH
F
O
HO
BnO
OPMP
30
20
10
0
-10
-20
-30
-40
-50
-60
-70
-80
-90
-100
-110
-120
-130
-140
-150
-160
-170
-180
-190
-200
-210
-220
-230

¹⁹F NMR

p-Methoxyphenyl 3,4,6-tri-*O*-benzyl-2-deoxy-2-fluoro-β-D-mannopyranoside (S7)

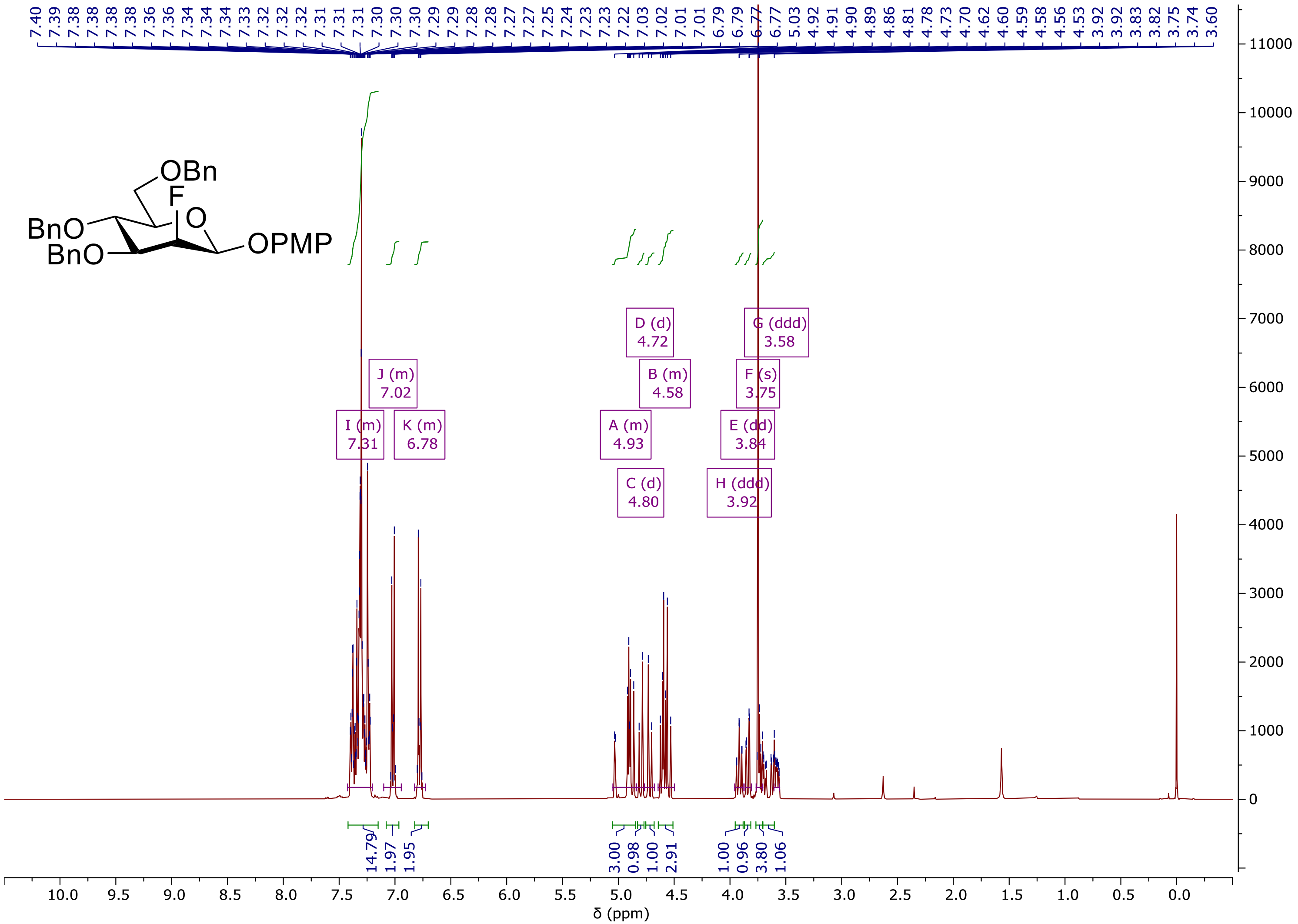

¹H NMR

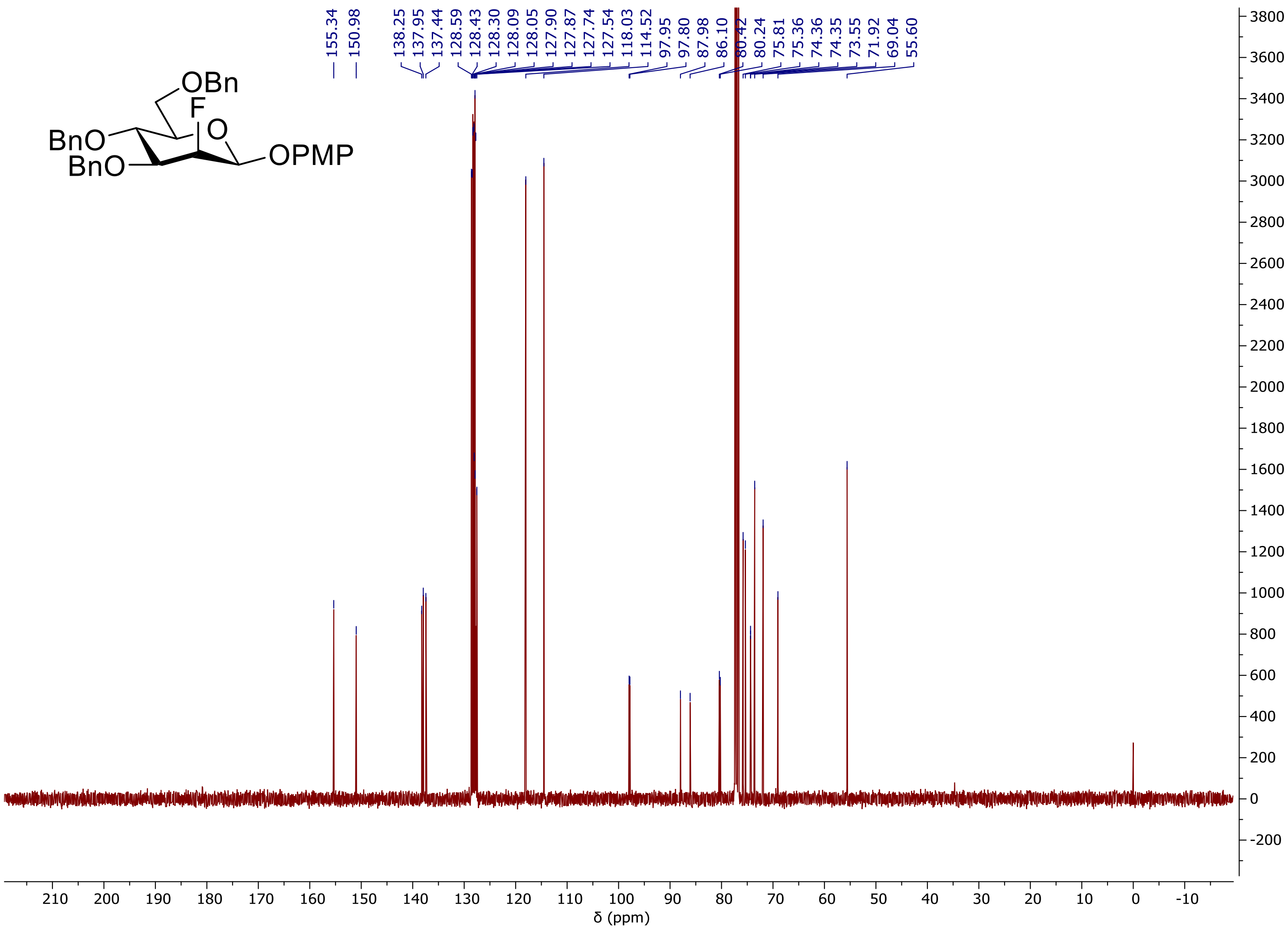
155.34
150.98
138.25
137.95
137.44
128.59
128.43
128.30
128.09
128.05
127.90
127.87
127.74
127.54
118.03
114.52
97.95
97.80
87.98
86.10
80.42
80.24
75.81
75.36
74.36
74.35
73.55
71.92
69.04
55.60
OBn
F
O
BnO
BnO
OPMP
δ (ppm)

^{13}C NMR

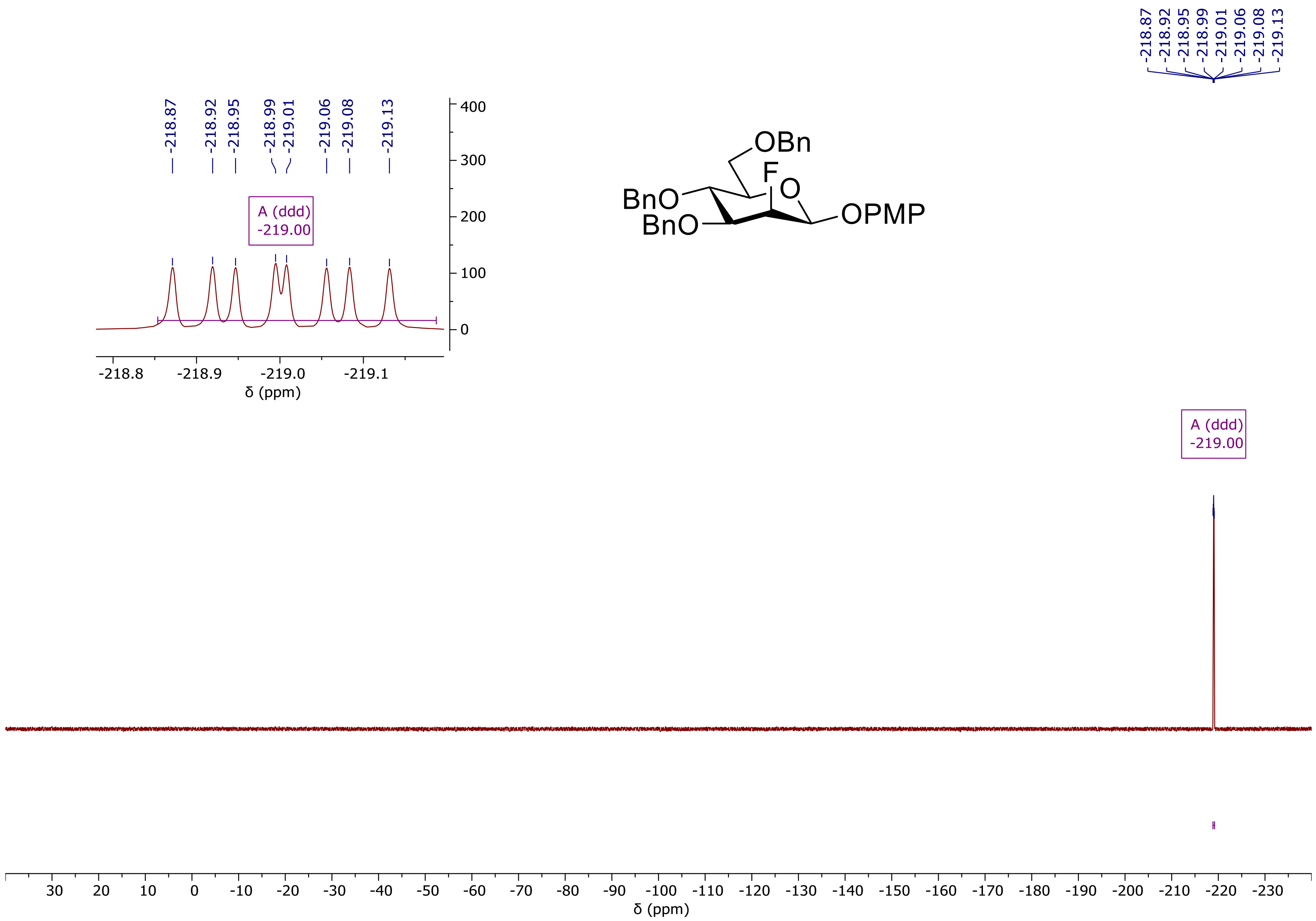
-218.87
-218.92
-218.95
-218.99
-219.01
-219.06
-219.08
-219.13
A (ddd)
-219.00
-218.8
-218.9
-219.0
-219.1
δ (ppm)
OBn
F
O
BnO
BnO
OPMP
30 20 10 0 -10 -20 -30 -40 -50 -60 -70 -80 -90 -100 -110 -120 -130 -140 -150 -160 -170 -180 -190 -200 -210 -220 -230
δ (ppm)

^{19}F NMR

3,4,6-tri-*O*-benzyl-2-deoxy-2-fluoro-α-D-mannopyranoside (5)

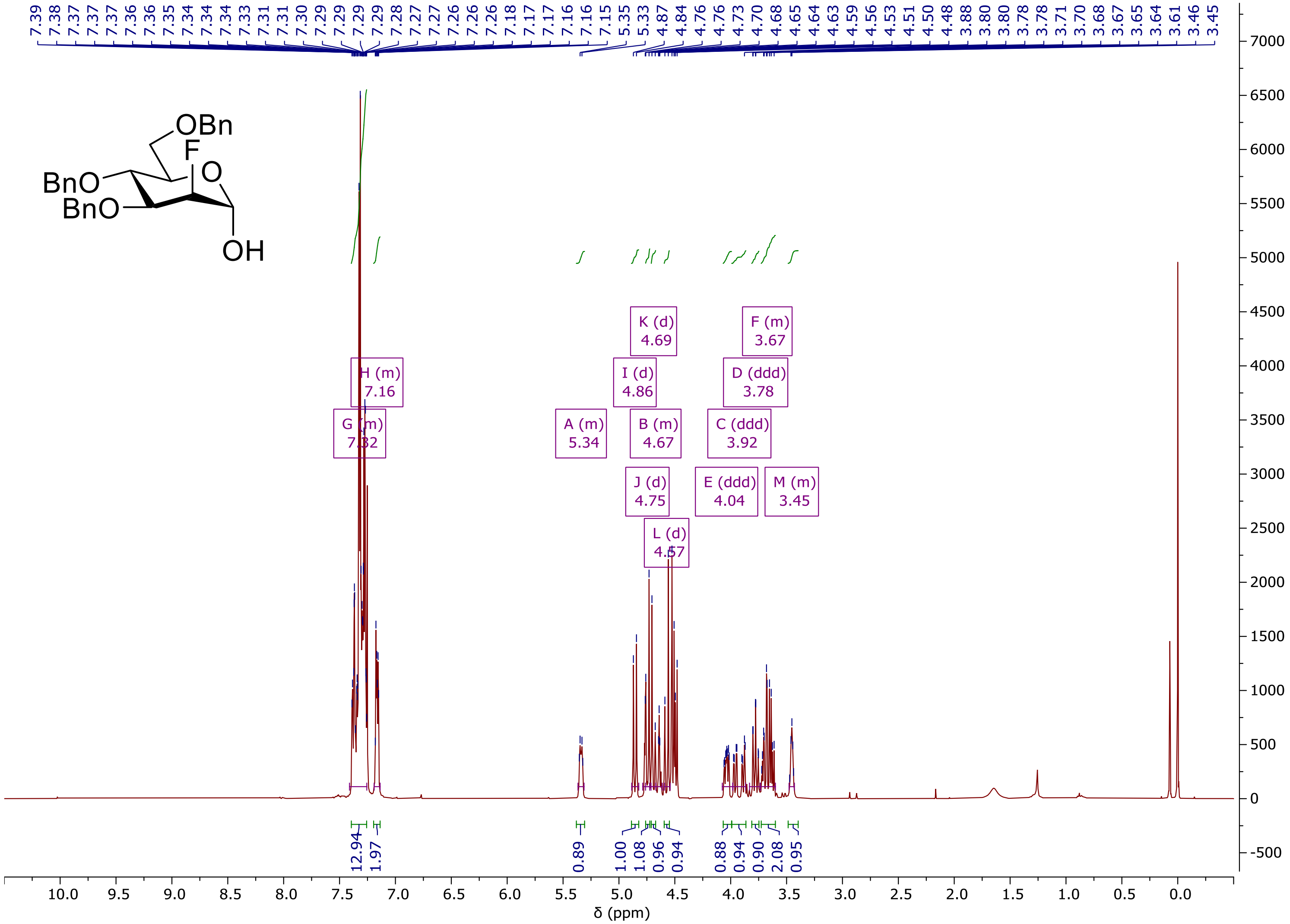

¹H NMR

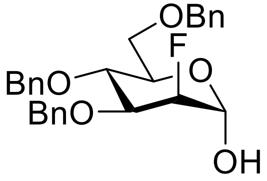

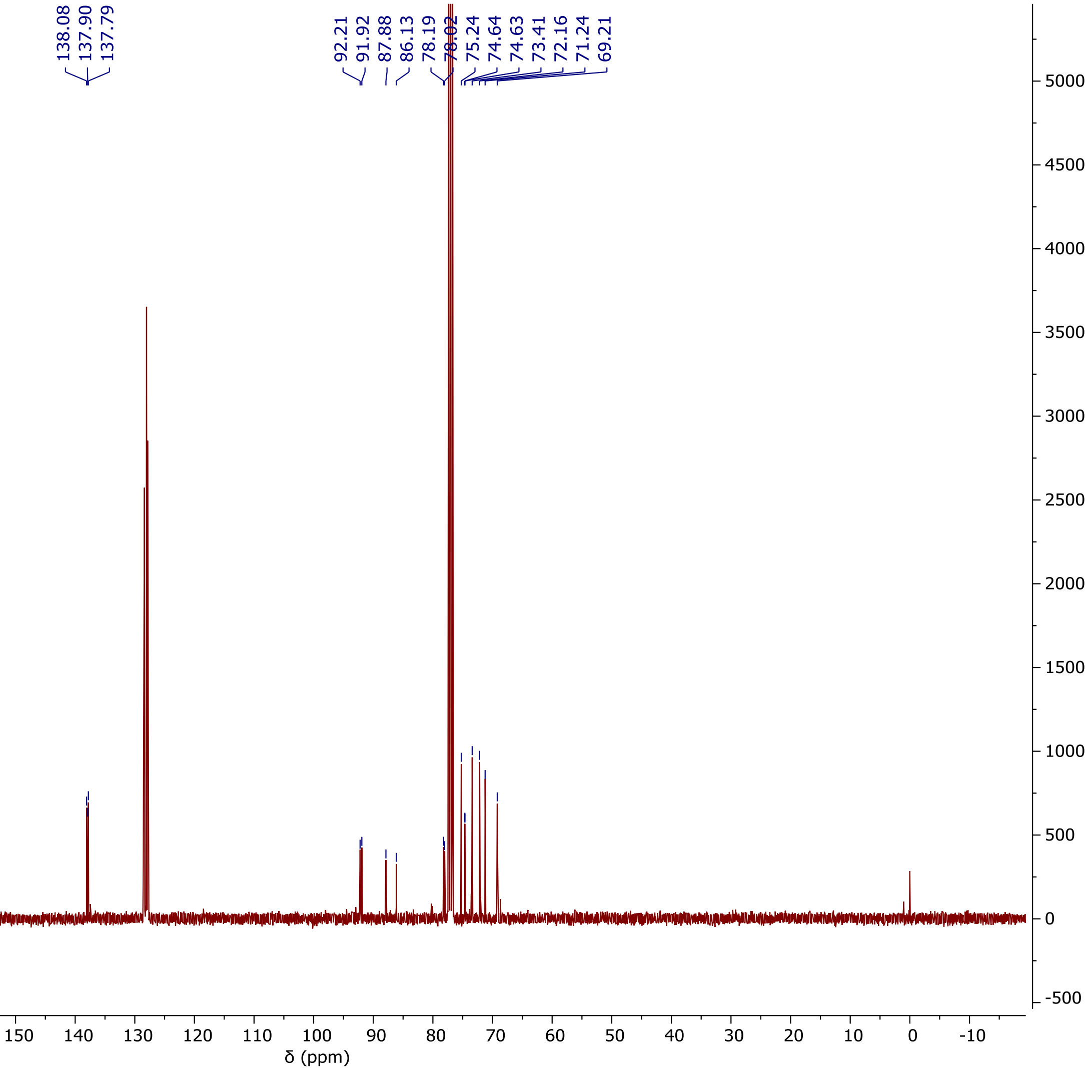
138.08
137.90
137.79
92.21
91.92
87.88
86.13
78.19
78.02
75.24
74.64
74.63
73.41
72.16
71.24
69.21
δ (ppm)

^{13}C NMR

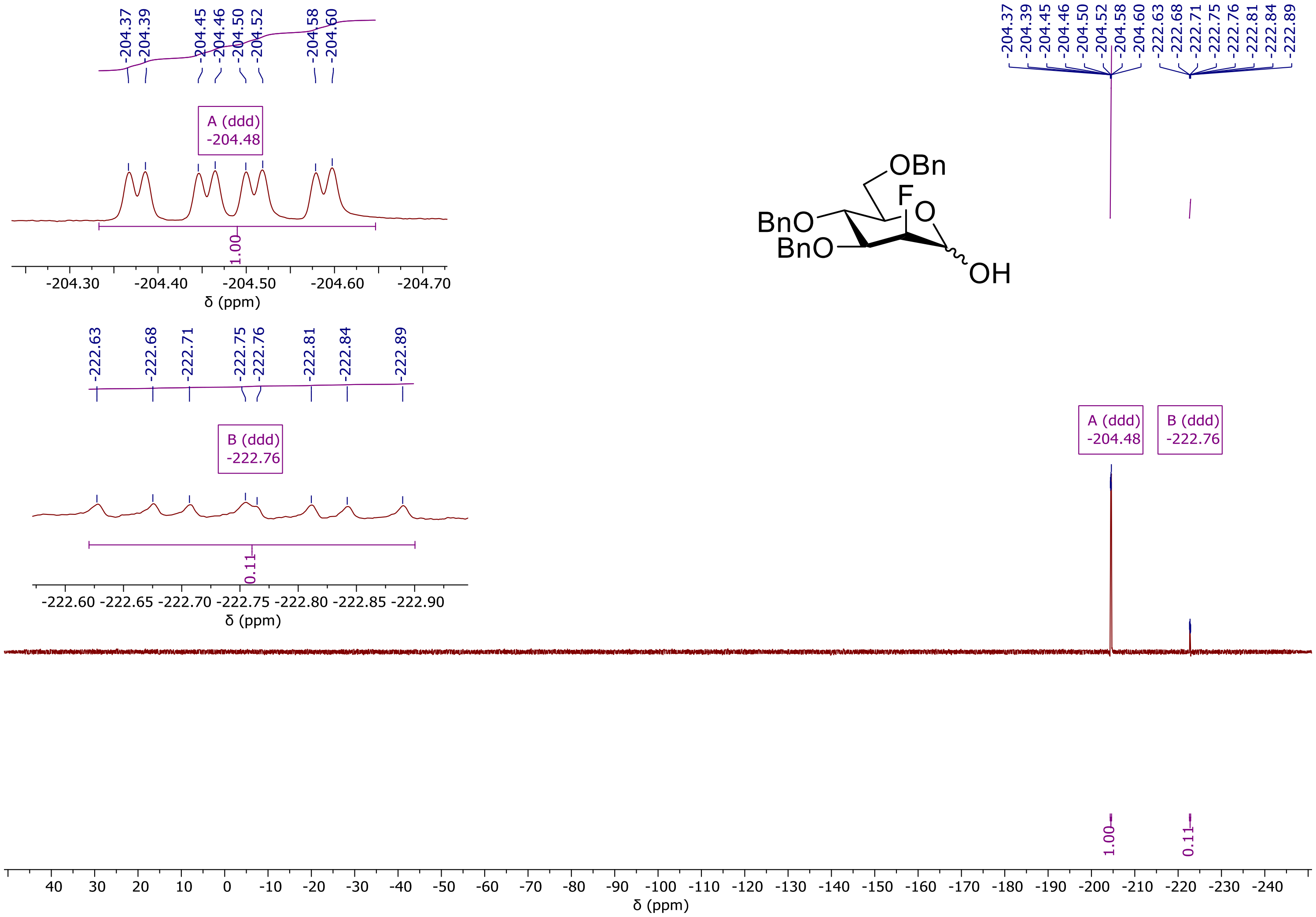

^{19}F NMR

Di-*O*-phenyl (2-deoxy-2-fluoro-3,4,6-tri-*O*-benzyl)-1-phosphate-α-D-mannopyranoside (S8)

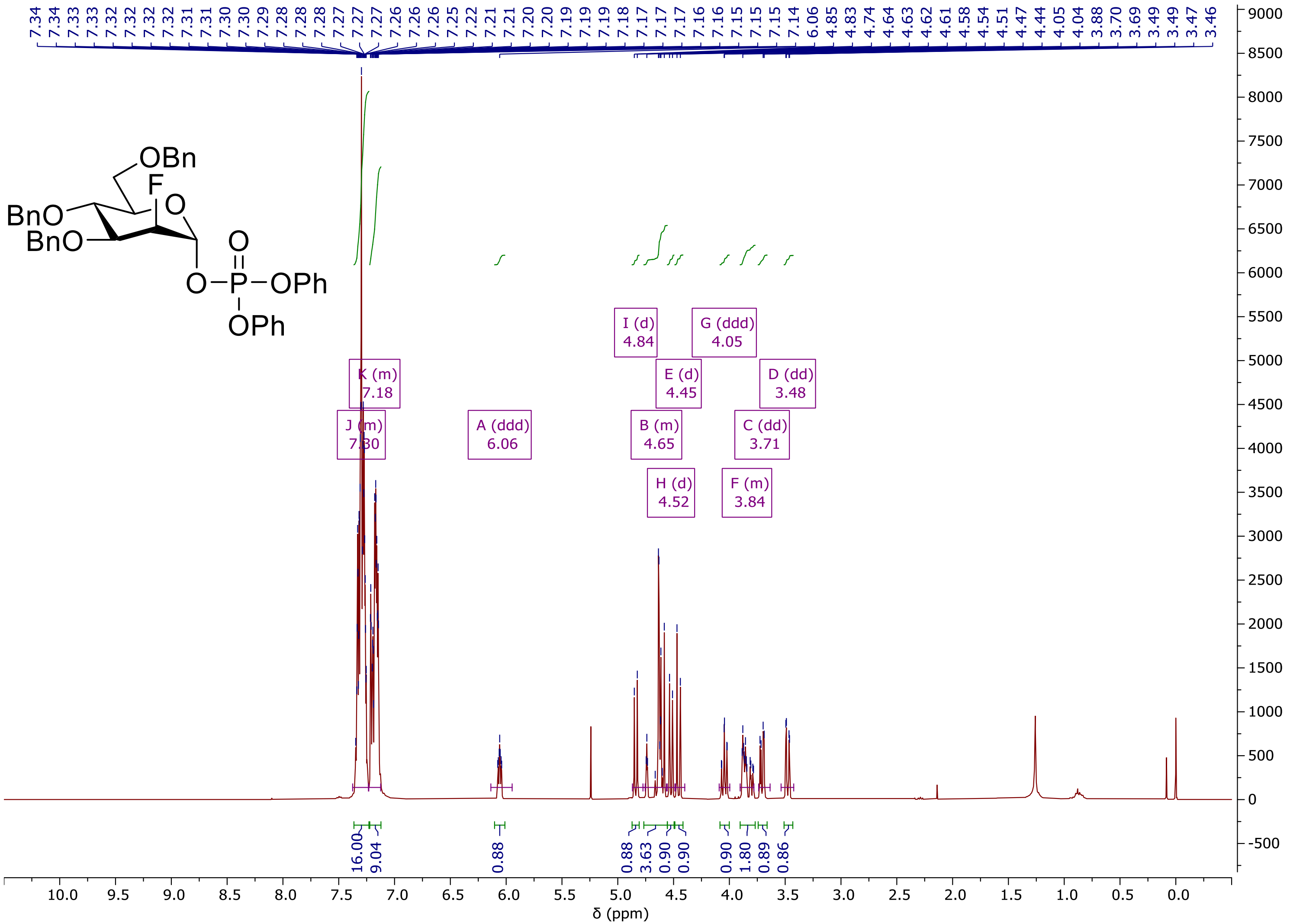

¹H NMR

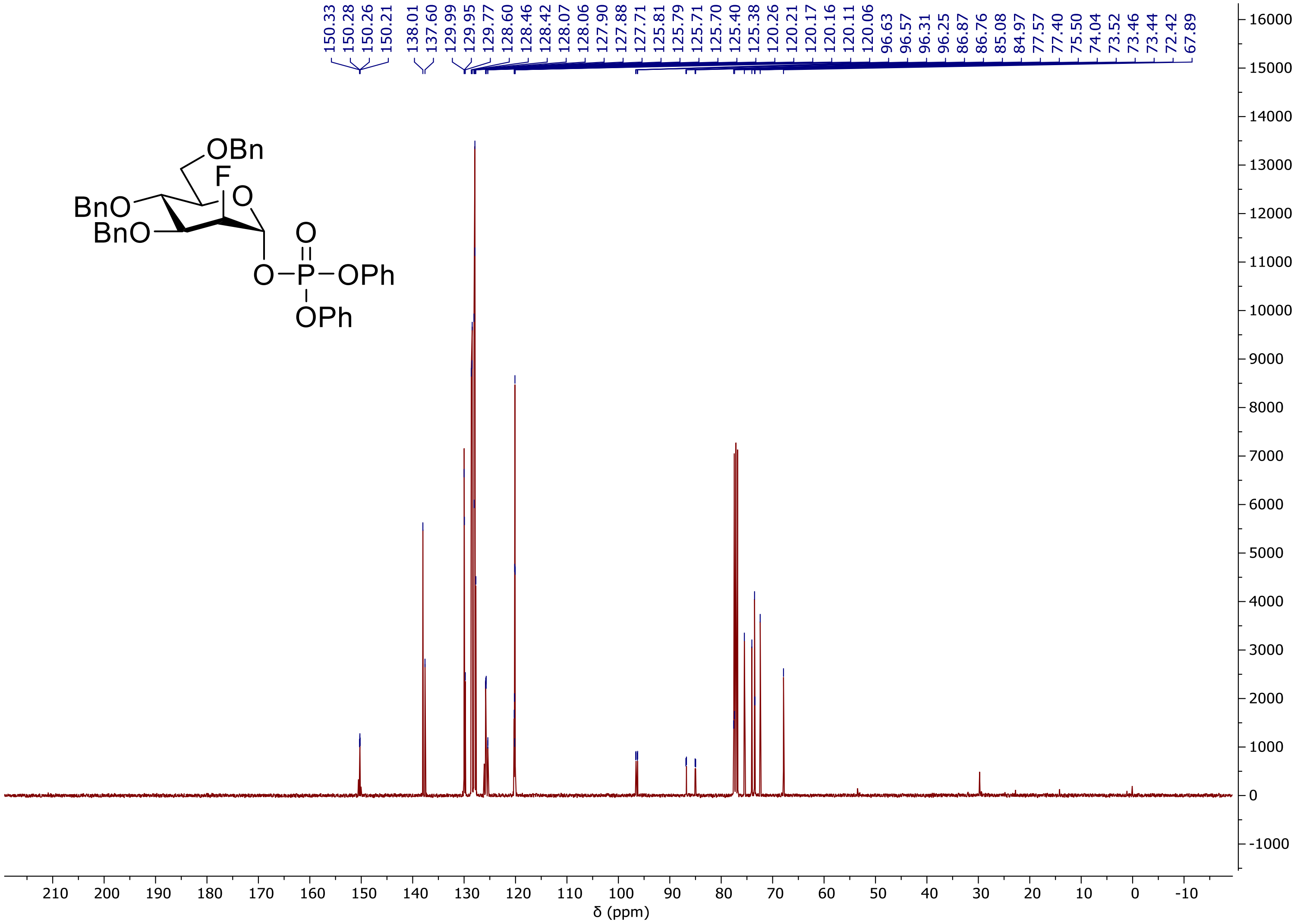
150.33
150.28
150.26
150.21
138.01
137.60
129.99
129.95
129.77
128.60
128.46
128.42
128.07
128.06
127.90
127.88
127.71
125.81
125.79
125.71
125.70
125.40
125.38
120.26
120.21
120.17
120.16
120.11
120.06
96.63
96.57
96.31
96.25
86.87
86.76
85.08
84.97
77.57
77.40
75.50
74.04
73.52
73.46
73.44
72.42
67.89
OBn
F
O
BnO
BnO
O
O–P–OPh
OPh
δ (ppm)

^{13}C NMR

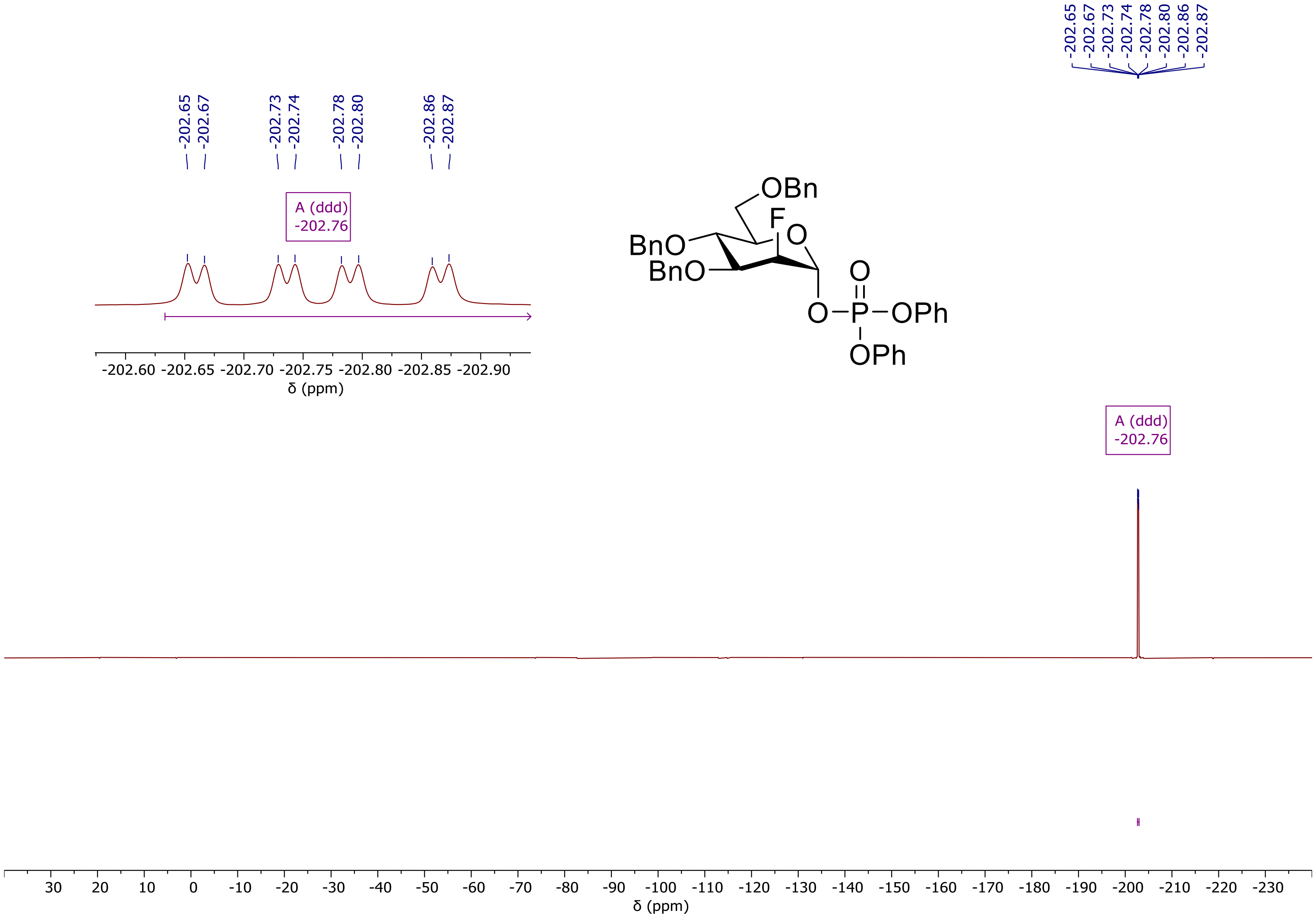
-202.65
-202.67
-202.73
-202.74
-202.78
-202.80
-202.86
-202.87
A (ddd)
-202.76
-202.60 -202.65 -202.70 -202.75 -202.80 -202.85 -202.90
δ (ppm)
OBn
F
O
BnO
BnO
O
O
P
OPh
OPh
30 20 10 0 -10 -20 -30 -40 -50 -60 -70 -80 -90 -100 -110 -120 -130 -140 -150 -160 -170 -180 -190 -200 -210 -220 -230
δ (ppm)

^{19}F NMR

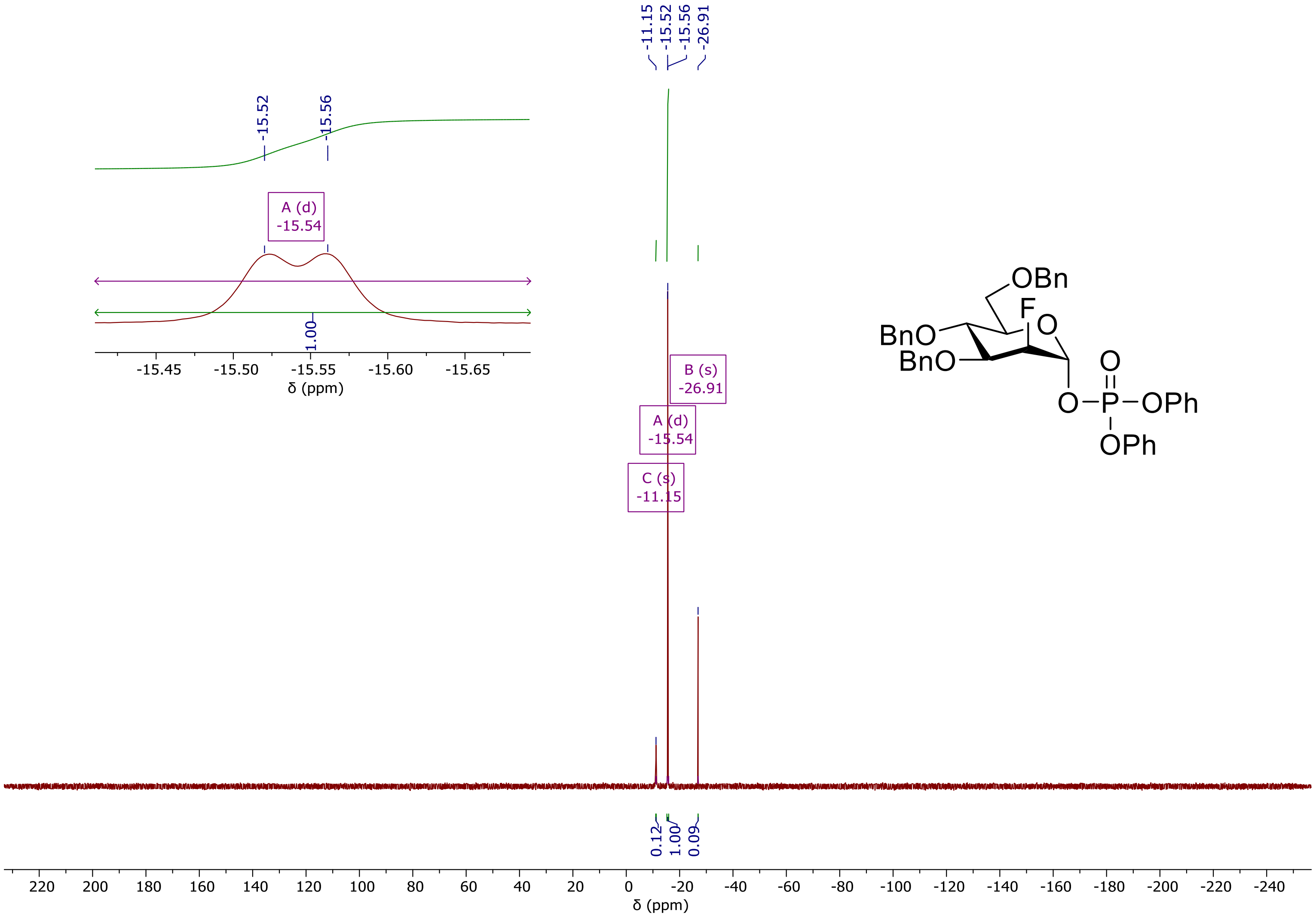
-11.15
-15.52
-15.56
-26.91
A (d)
-15.54
B (s)
-26.91
C (s)
-11.15
0.12
1.00
0.09
-15.45
-15.50
-15.55
-15.60
-15.65
δ (ppm)
220
200
180
160
140
120
100
80
60
40
20
0
-20
-40
-60
-80
-100
-120
-140
-160
-180
-200
-220
-240
δ (ppm)
OBn
F
O
BnO
BnO
O
O
P
OPh
OPh

^{31}P NMR

2-Deoxy-2-fluoro-α-D-mannose-1-phosphate (bis ammonium salt) (6)

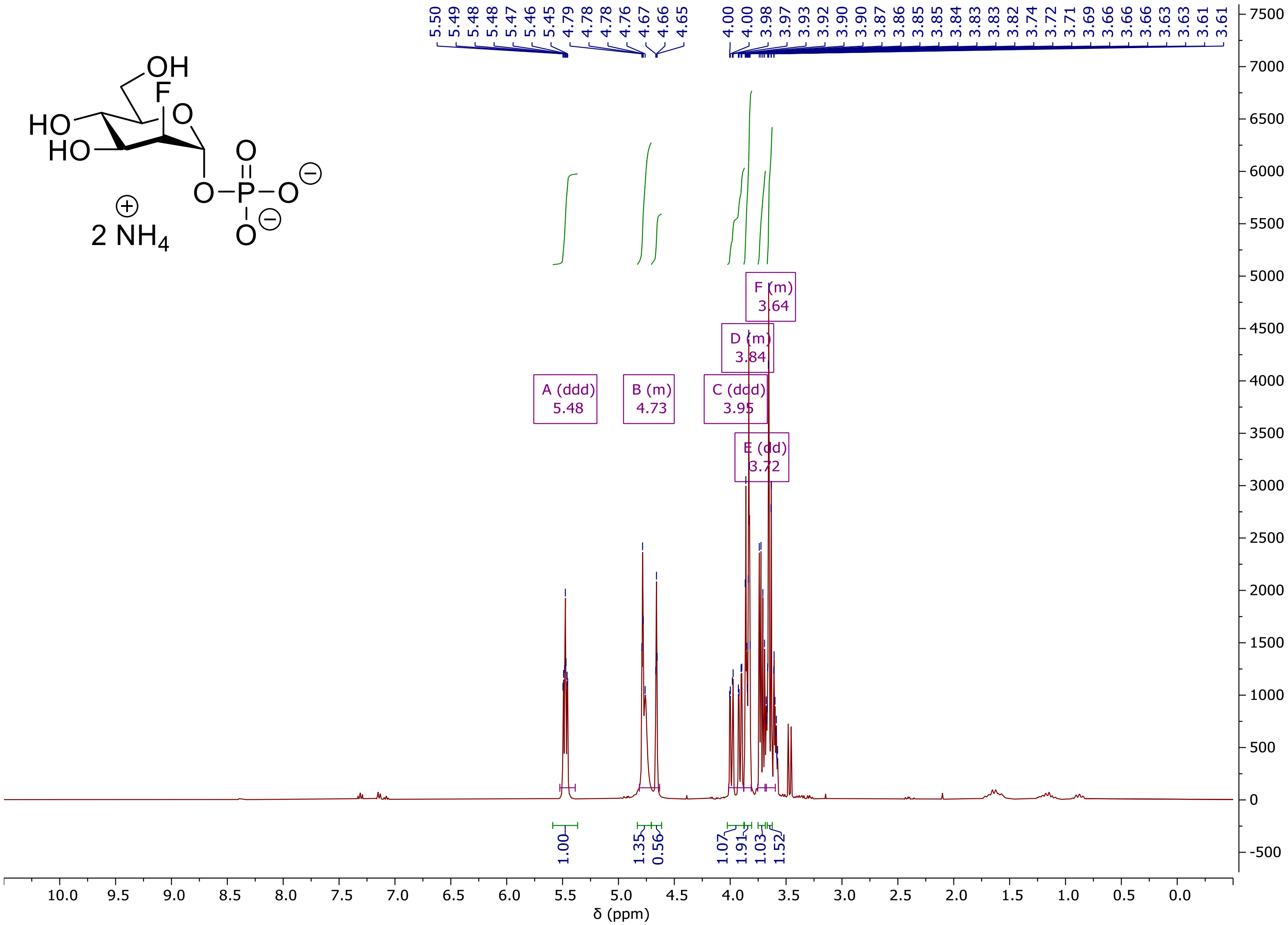

^{1}H NMR

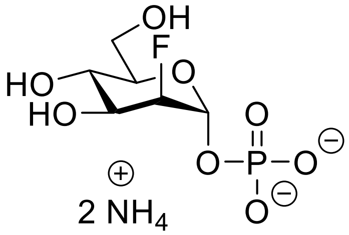

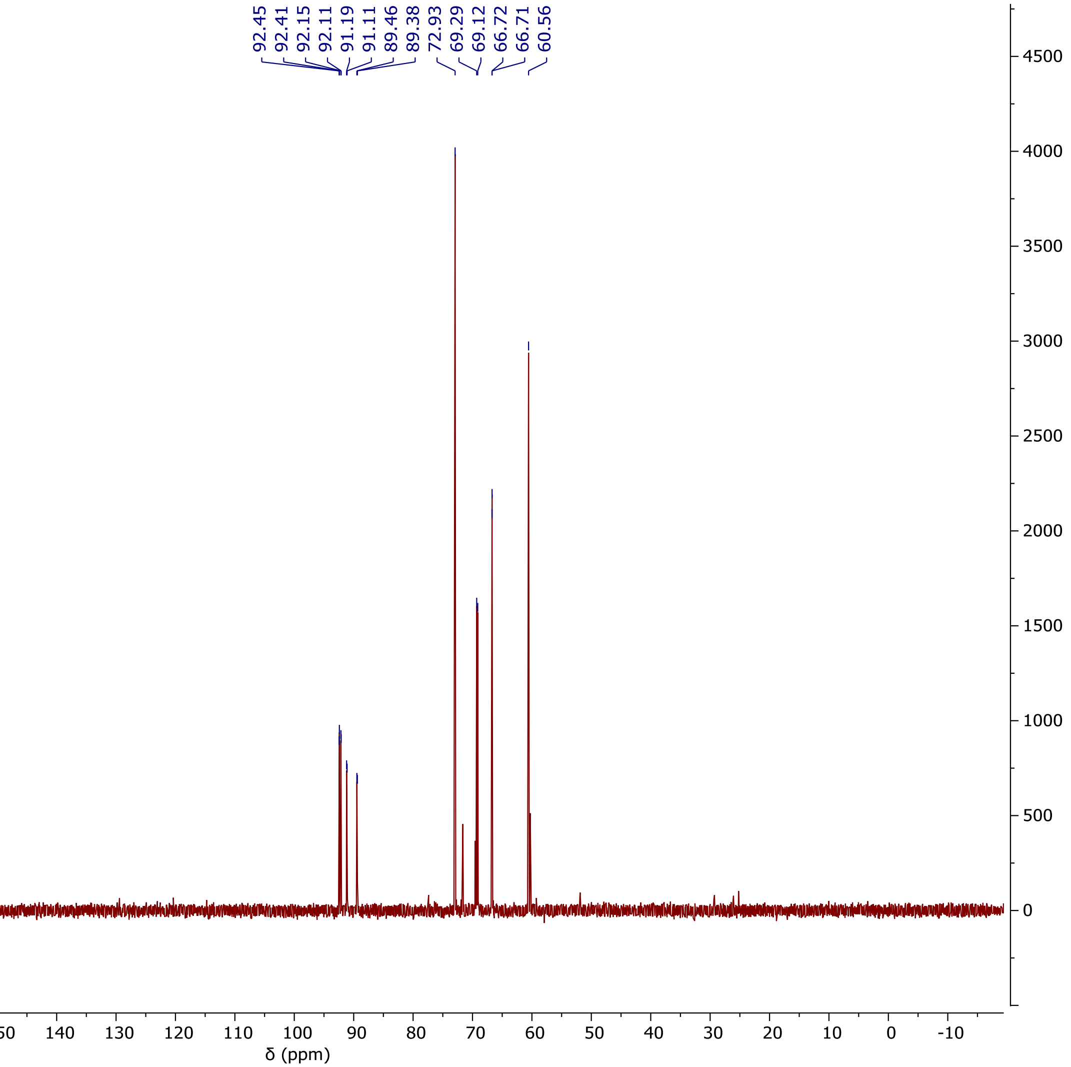

92.45
92.41
92.15
92.11
91.19
91.11
89.46
89.38
72.93
69.29
69.12
66.72
66.71
60.56
4500
4000
3500
3000
2500
2000
1500
1000
500
0
210 200 190 180 170 160 150 140 130 120 110 100 90 80 70 60 50 40 30 20 10 0 -10
δ (ppm)

^{13}C NMR

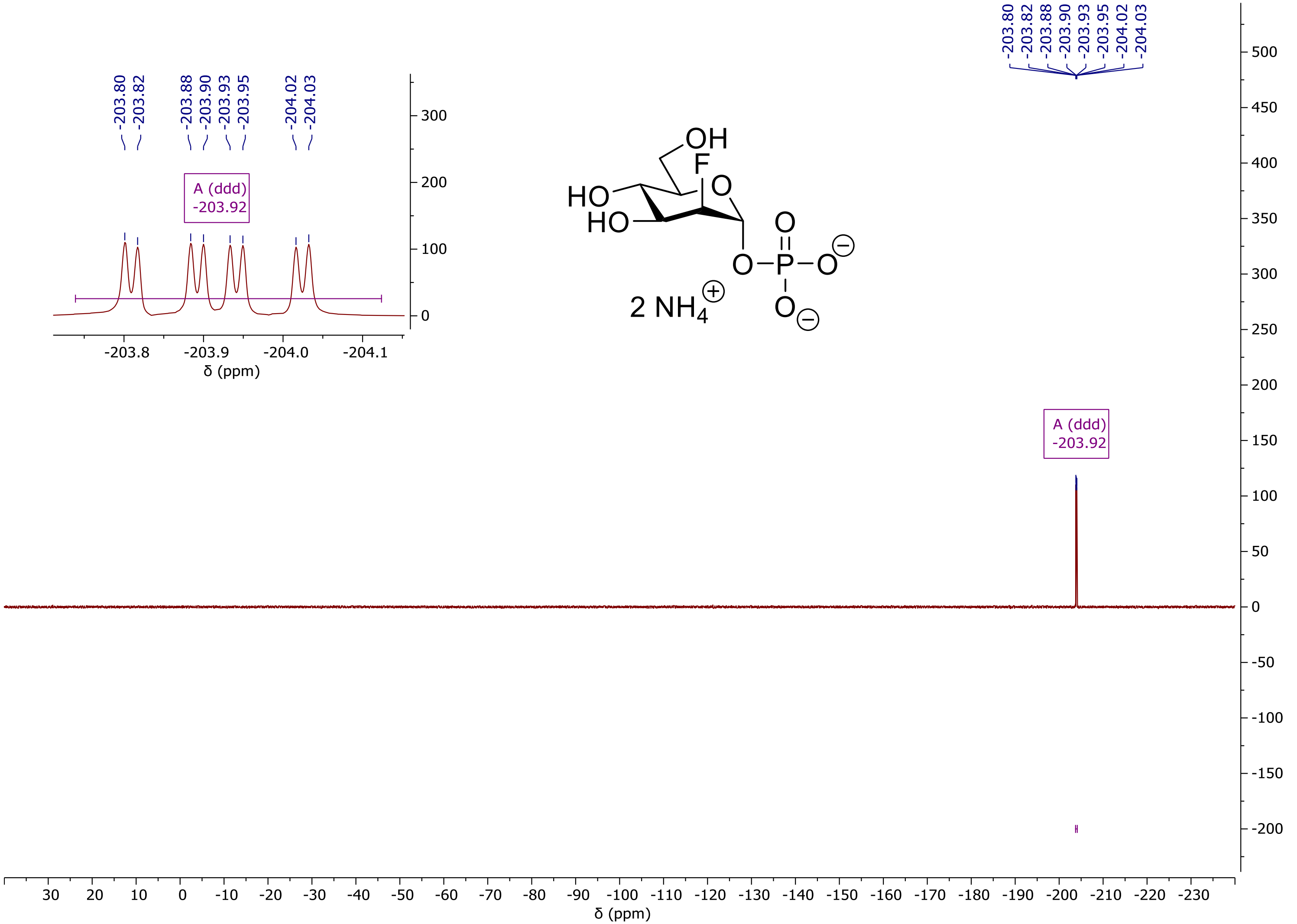
-203.80
-203.82
-203.88
-203.90
-203.93
-203.95
-204.02
-204.03
A (ddd)
-203.92
δ (ppm)
OH
F
O
HO
HO
O
O
P
O
2 NH4

^{19}F NMR

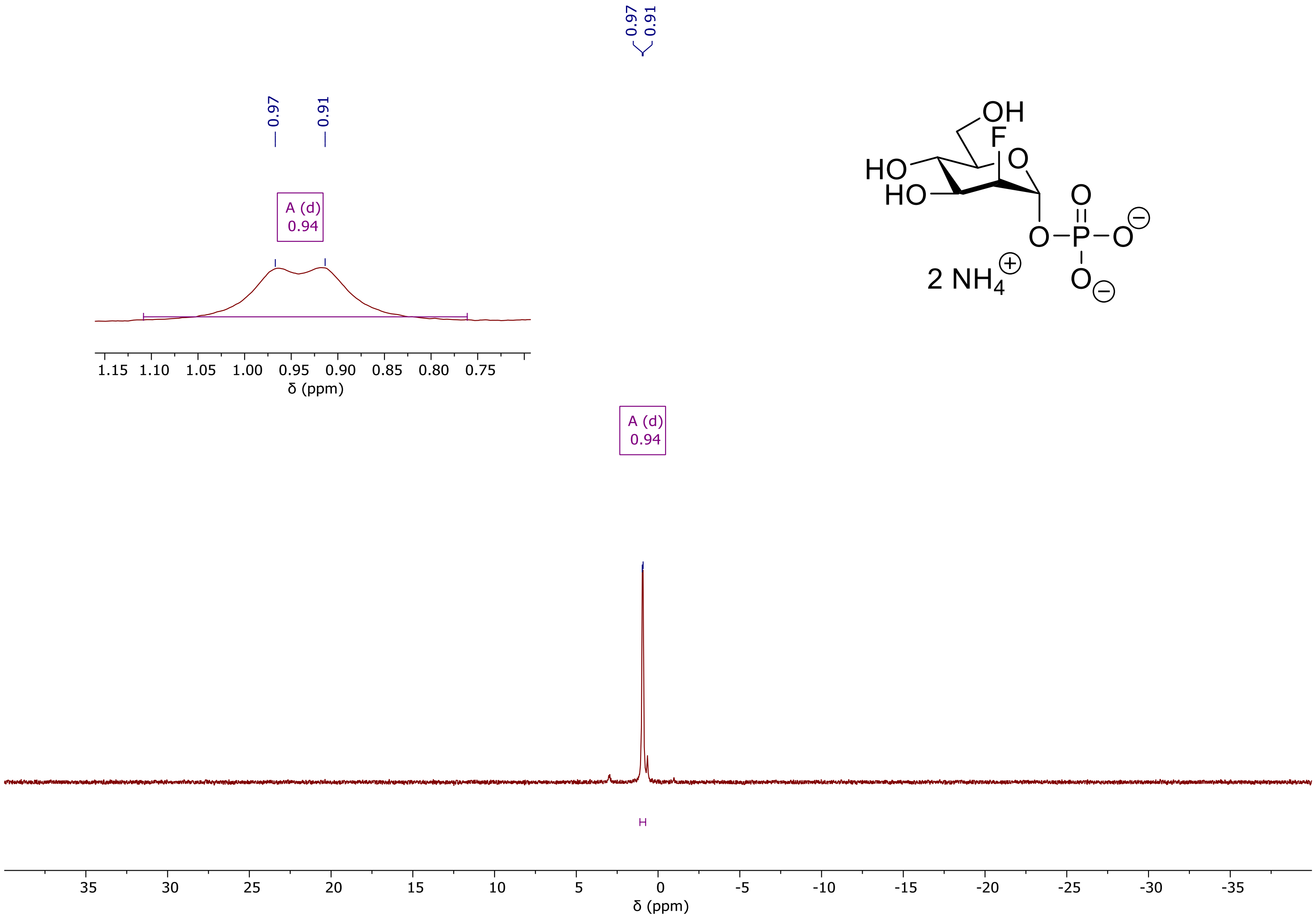
0.97
0.91
A (d)
0.94
1.15 1.10 1.05 1.00 0.95 0.90 0.85 0.80 0.75
δ (ppm)
35 30 25 20 15 10 5 0 -5 -10 -15 -20 -25 -30 -35
δ (ppm)
OH
F
O
HO
HO
O
O
P
O
O
2 NH4

^{31}P NMR

2’-deoxy 2’-fluoro Guanosine Triphosphate (8)

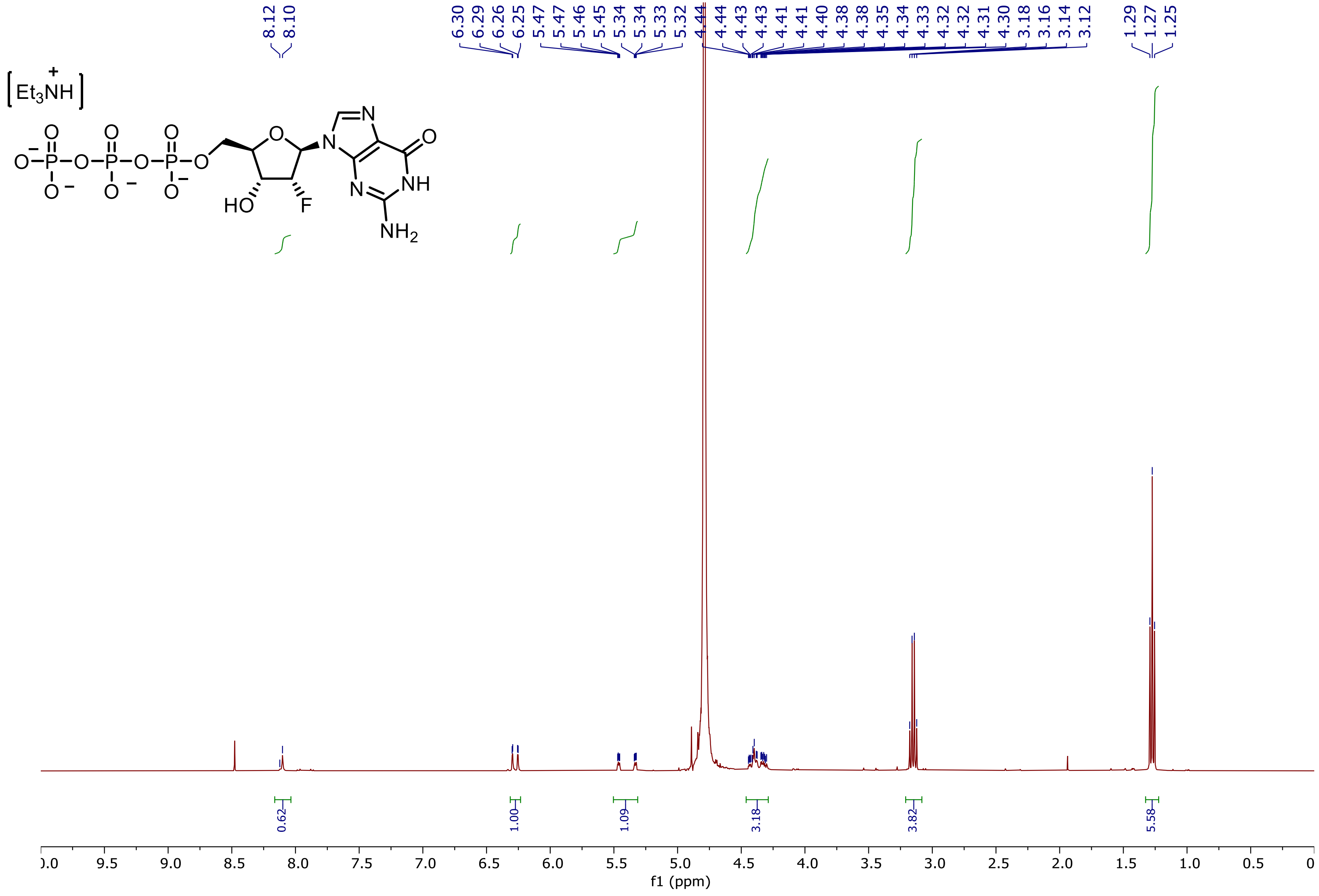

¹H NMR

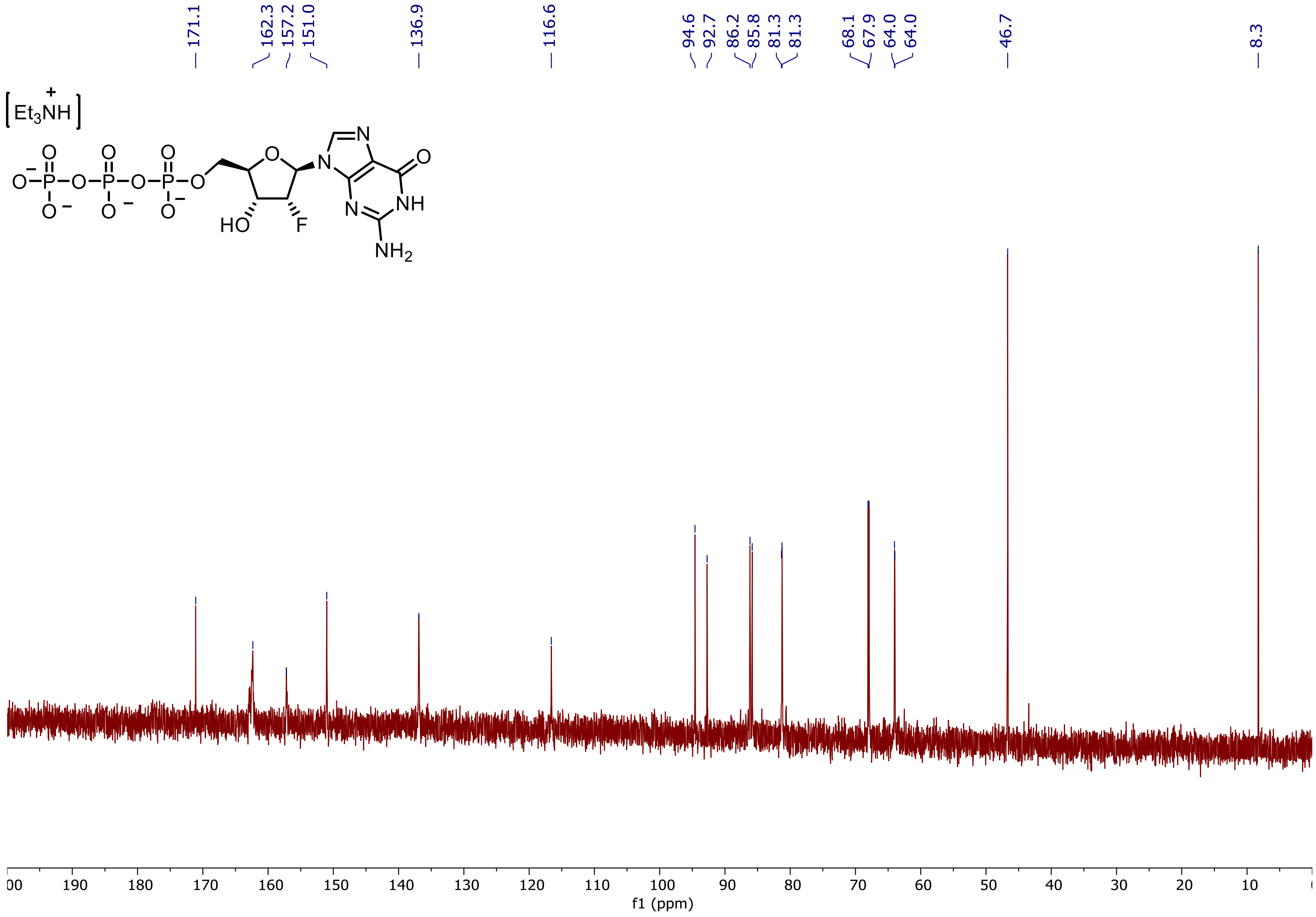
171.1
162.3
157.2
151.0
136.9
116.6
94.6
92.7
86.2
85.8
81.3
81.3
68.1
67.9
64.0
64.0
46.7
8.3
Et3NH
HO
F
NH
NH2
f1 (ppm)

^{13}C NMR

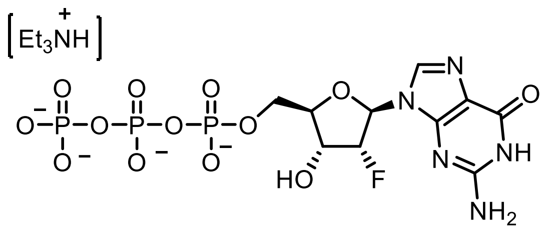

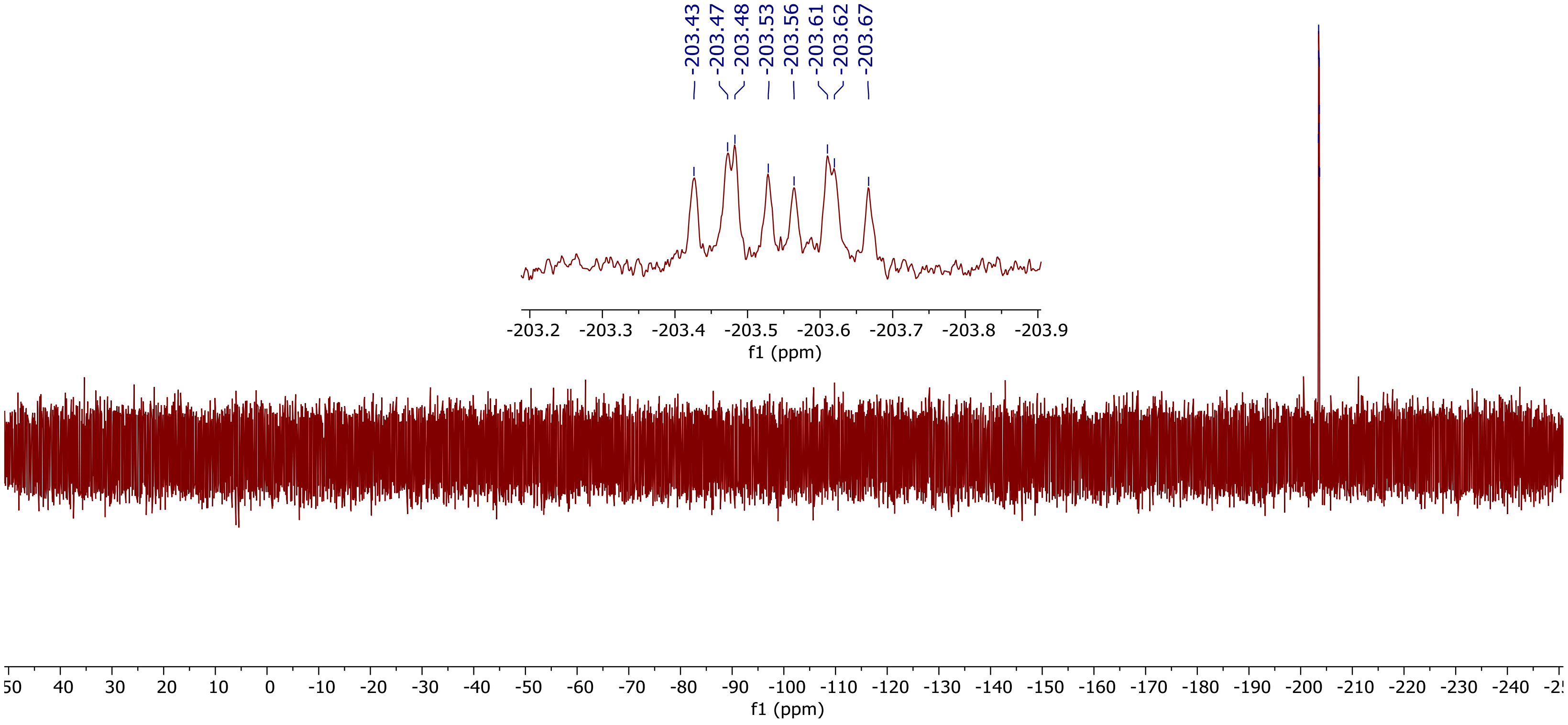

^{19}F NMR

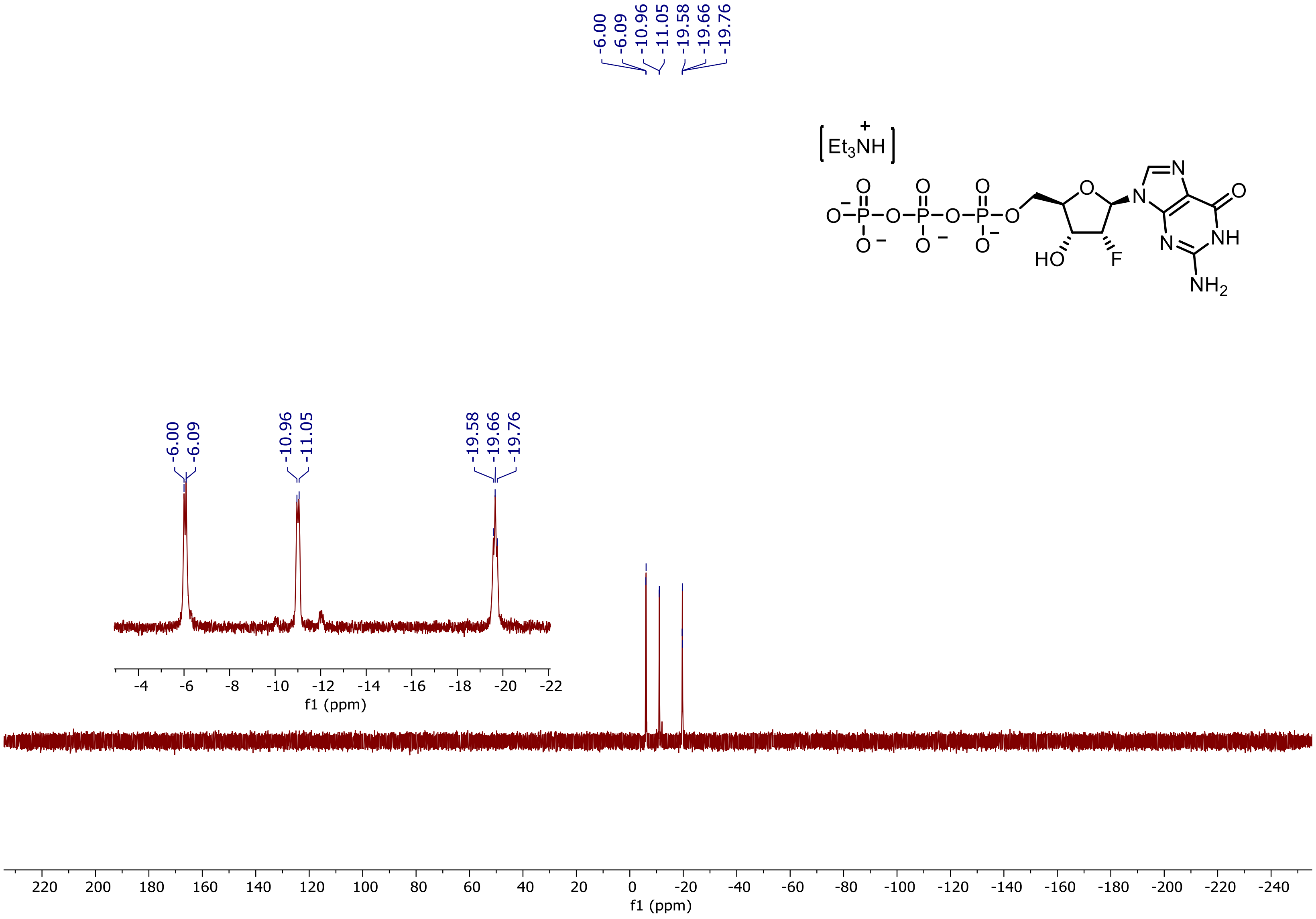

^{31}P NMR

8-CF$_3$-Guanosine Triphosphate (9)

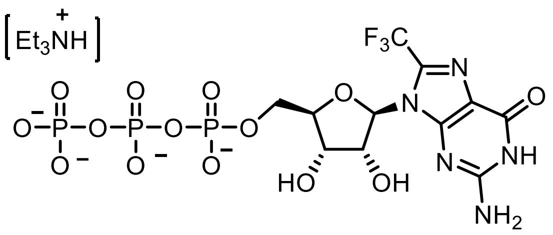

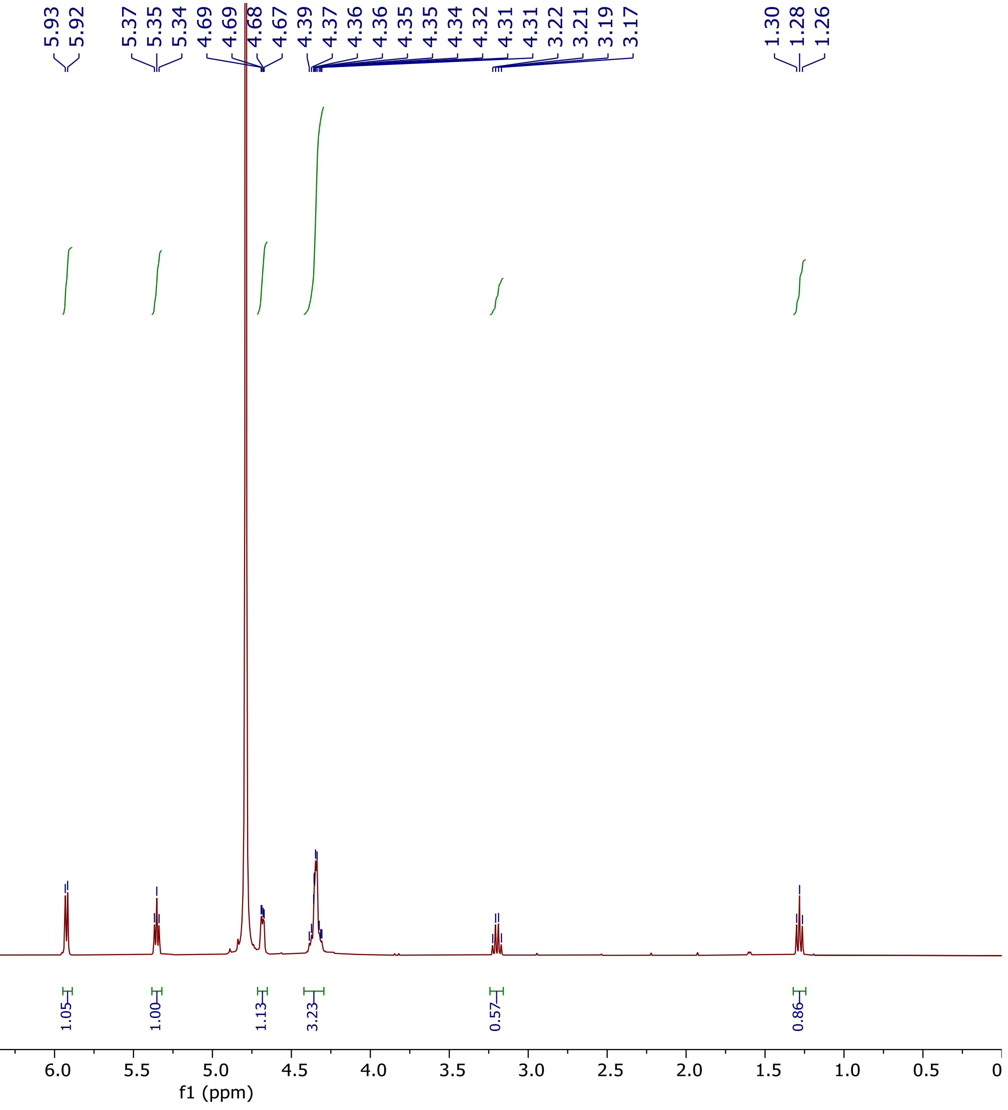

^{1}H NMR

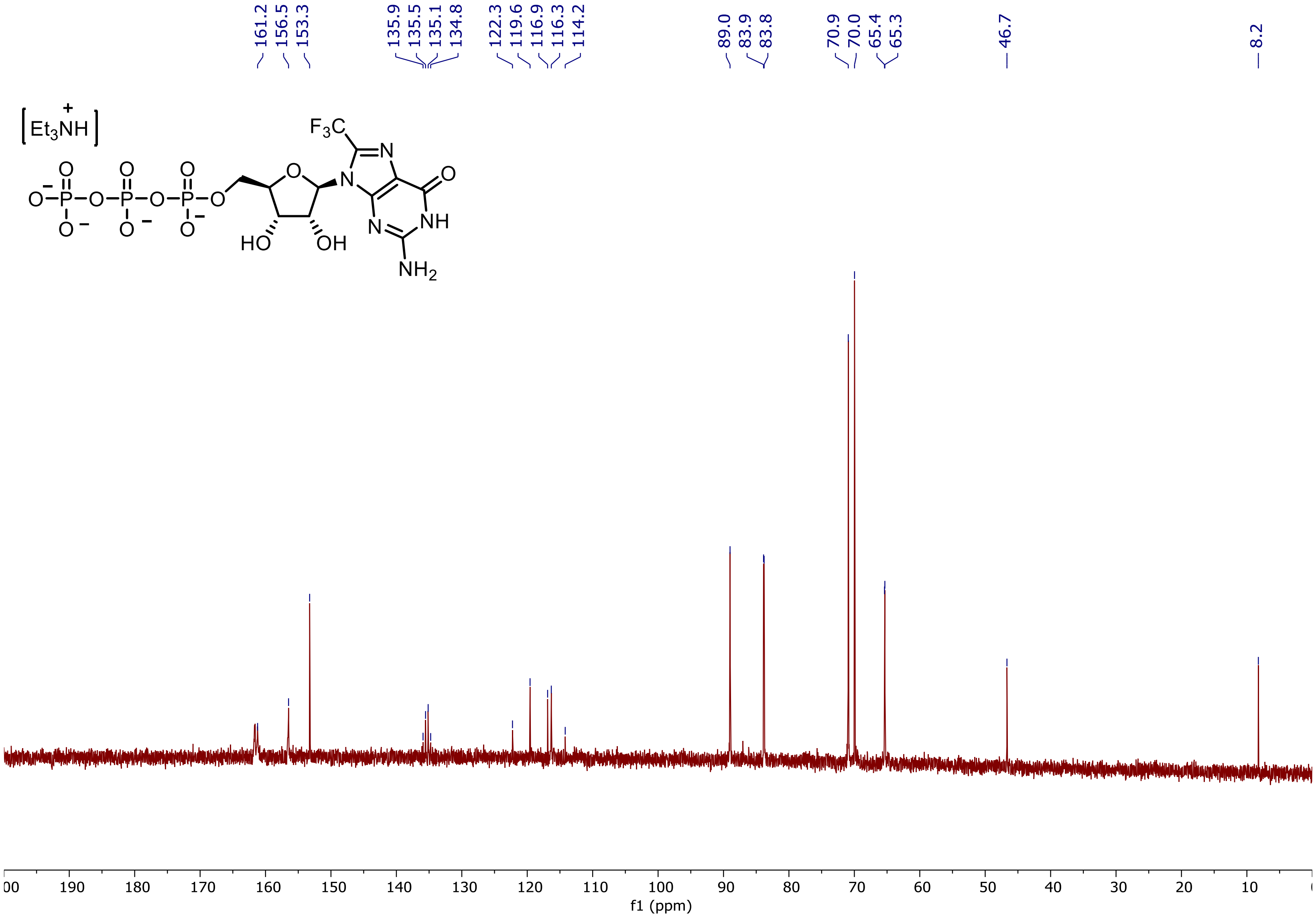
161.2
156.5
153.3
135.9
135.5
135.1
134.8
122.3
119.6
116.9
116.3
114.2
89.0
83.9
83.8
70.9
70.0
65.4
65.3
46.7
8.2
f1 (ppm)

^{13}C NMR

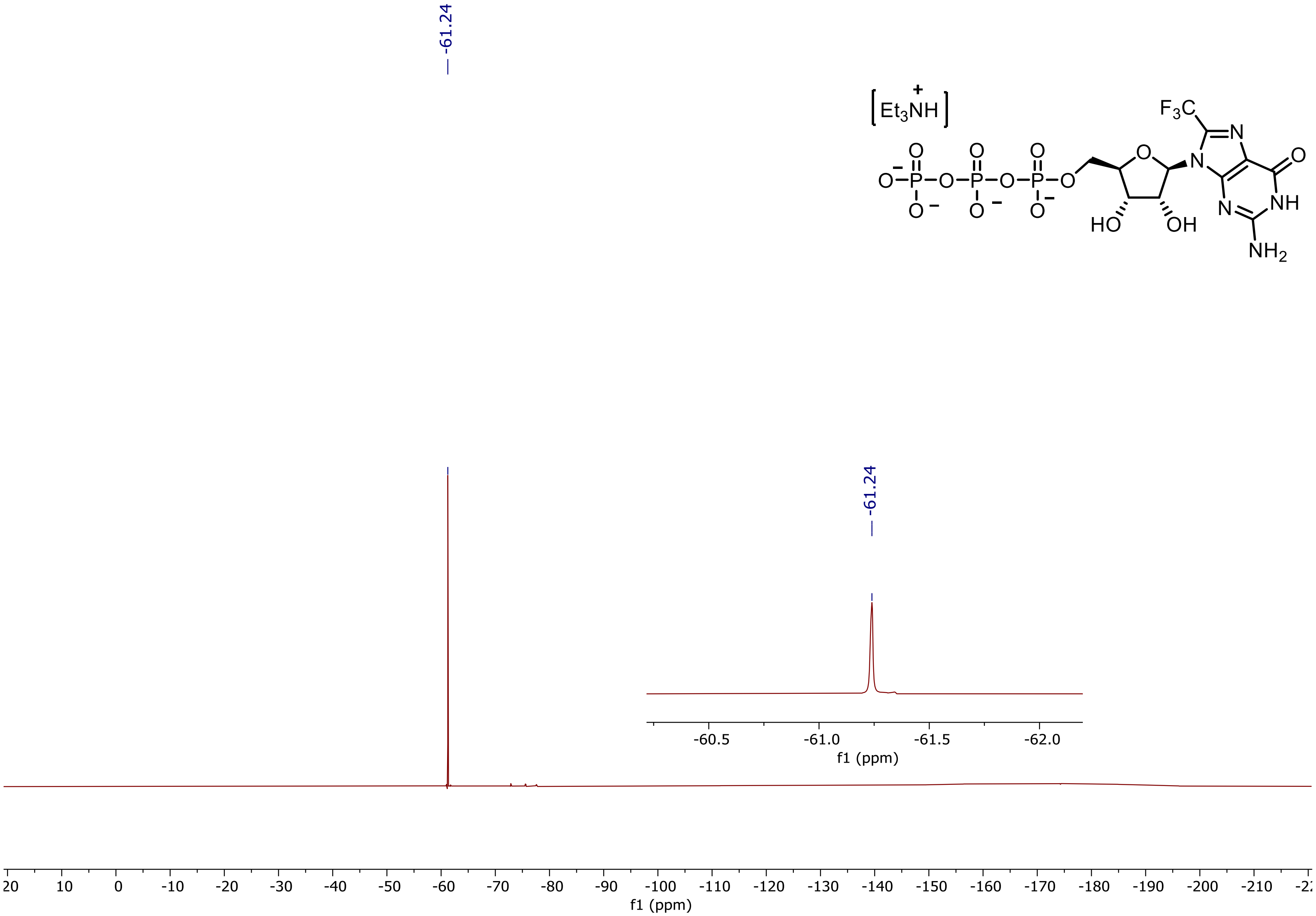

^{19}F NMR

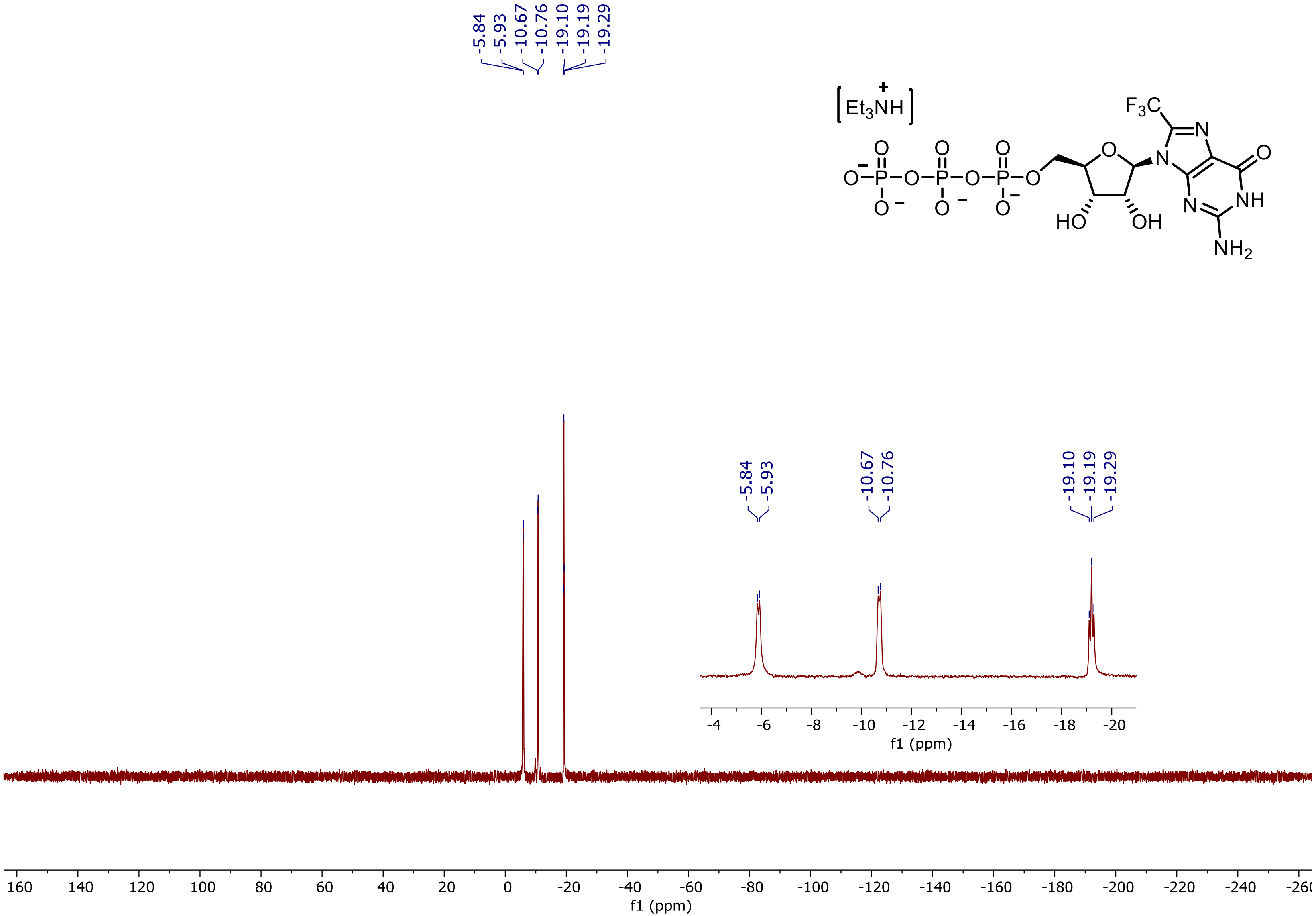
-5.84
-5.93
-10.67
-10.76
-19.10
-19.19
-19.29
Et3NH +
F3C
N
O
NH
NH2
HO
OH
-4 -6 -8 -10 -12 -14 -16 -18 -20
f1 (ppm)
160 140 120 100 80 60 40 20 0 -20 -40 -60 -80 -100 -120 -140 -160 -180 -200 -220 -240
f1 (ppm)

^{31}P NMR

Guanosine Diphosphate-3’’-deoxy-3’’-fluoro-mannose (10)

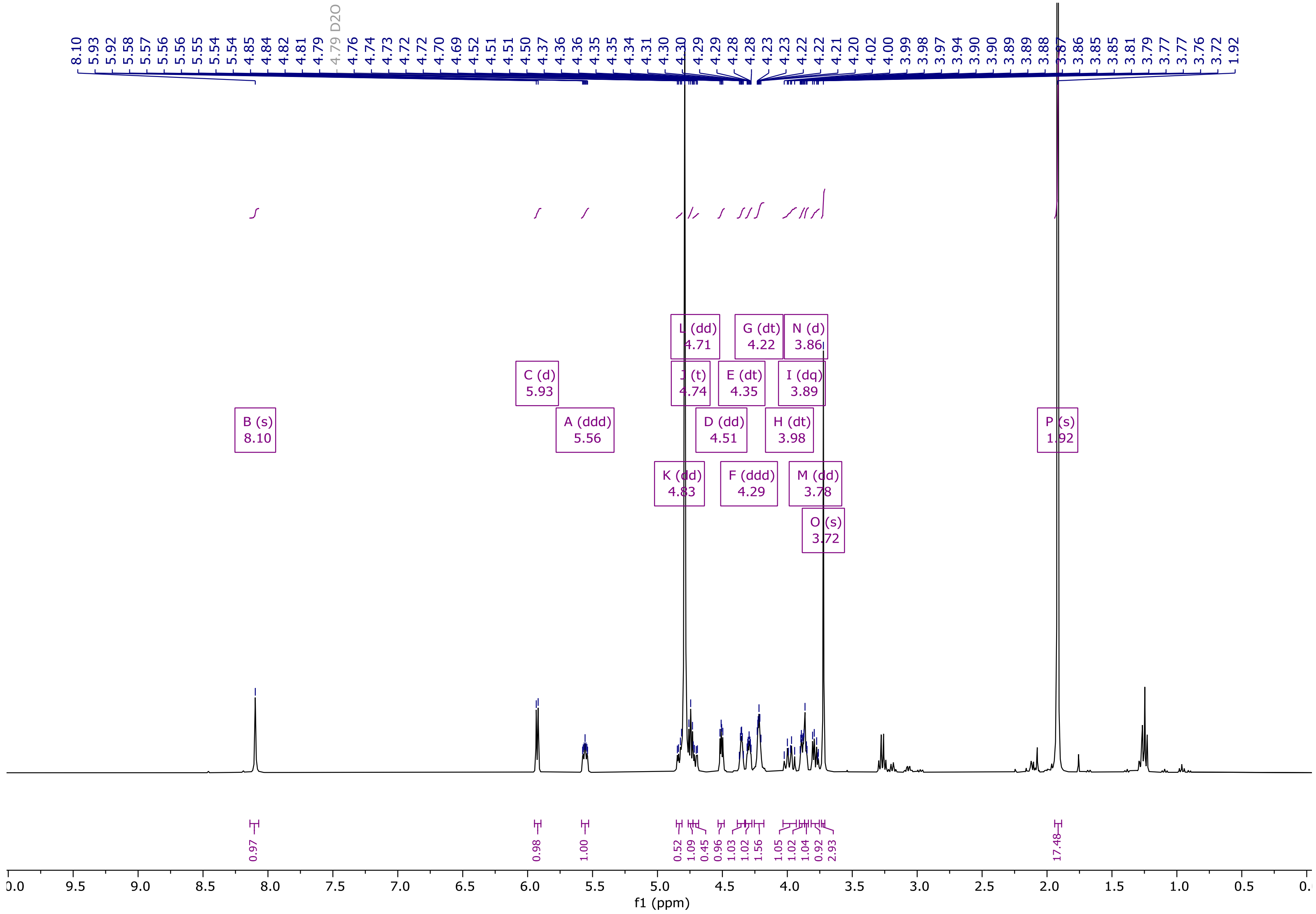

^{1}H NMR

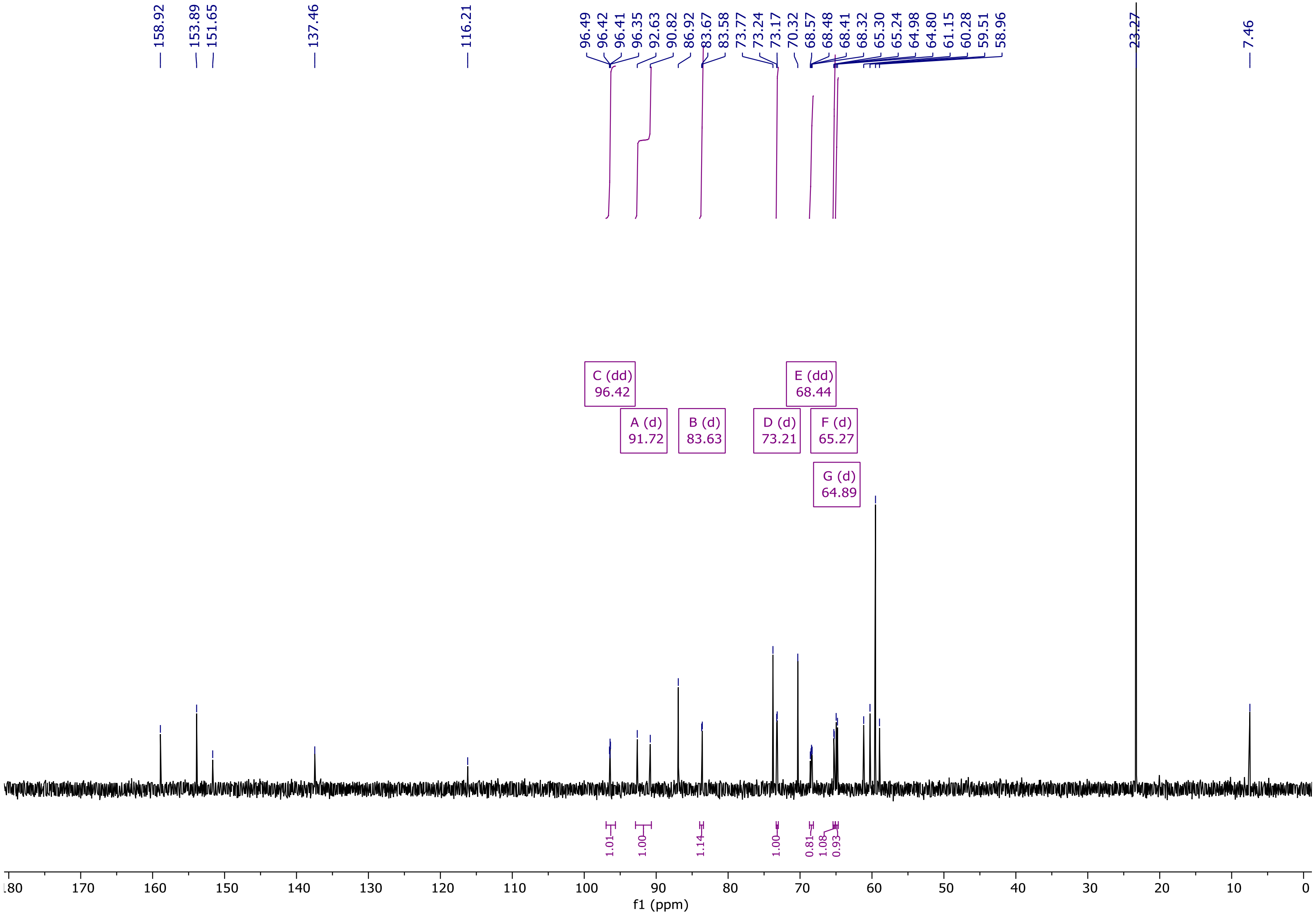
158.92
153.89
151.65
137.46
116.21
96.49
96.42
96.41
96.35
92.63
90.82
86.92
83.67
83.58
73.77
73.24
73.17
70.32
68.57
68.48
68.41
68.32
65.30
65.24
64.98
64.80
61.15
60.28
59.51
58.96
23.27
7.46
C (dd)
96.42
E (dd)
68.44
A (d)
91.72
B (d)
83.63
D (d)
73.21
F (d)
65.27
G (d)
64.89
1.01
1.00
1.14
1.00
0.81
1.08
0.93
f1 (ppm)

^{13}C NMR

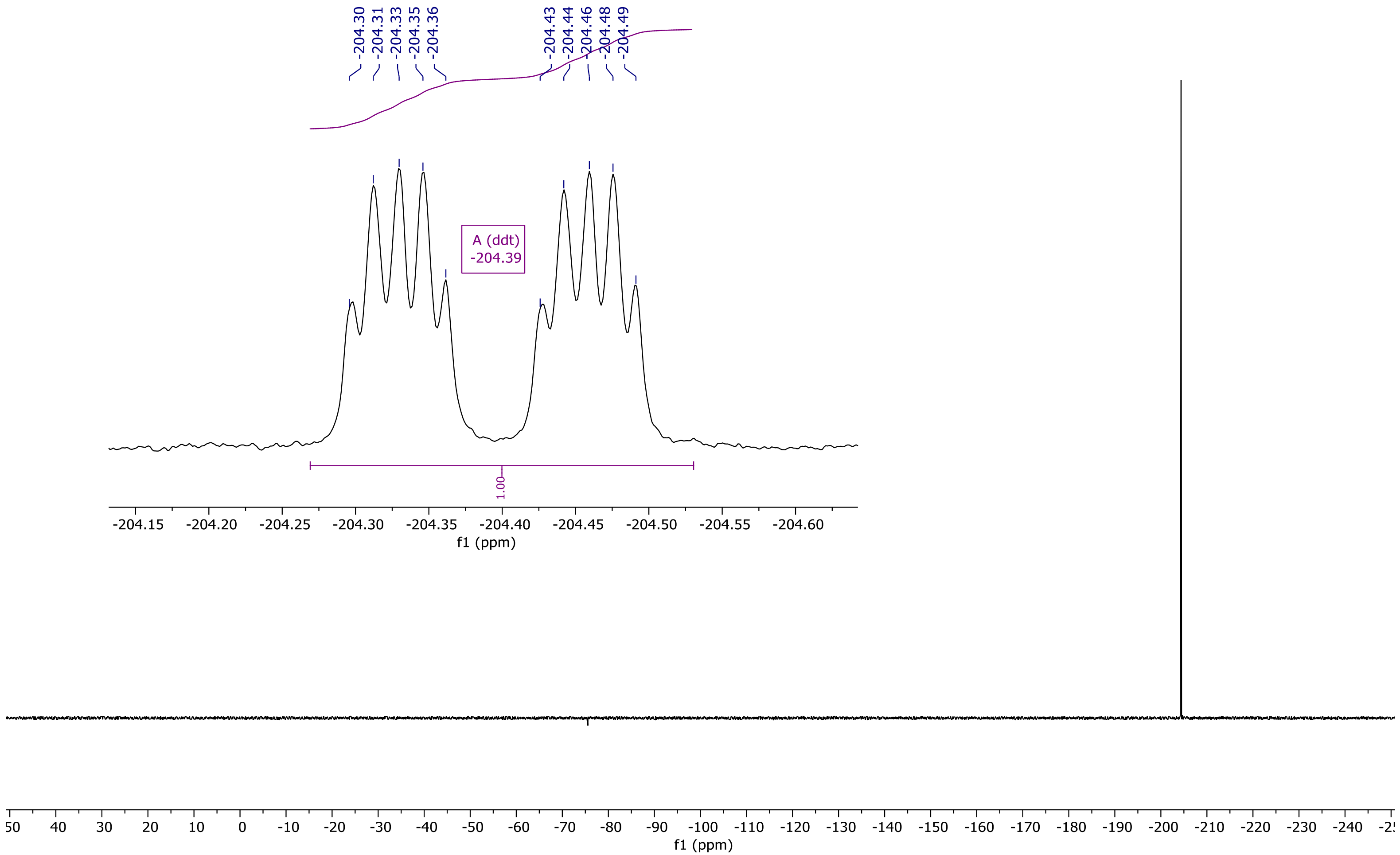
-204.30
-204.31
-204.33
-204.35
-204.36
-204.43
-204.44
-204.46
-204.48
-204.49
A (ddt)
-204.39
1.00
-204.15 -204.20 -204.25 -204.30 -204.35 -204.40 -204.45 -204.50 -204.55 -204.60
f1 (ppm)
50 40 30 20 10 0 -10 -20 -30 -40 -50 -60 -70 -80 -90 -100 -110 -120 -130 -140 -150 -160 -170 -180 -190 -200 -210 -220 -230 -240
f1 (ppm)

^{19}F NMR

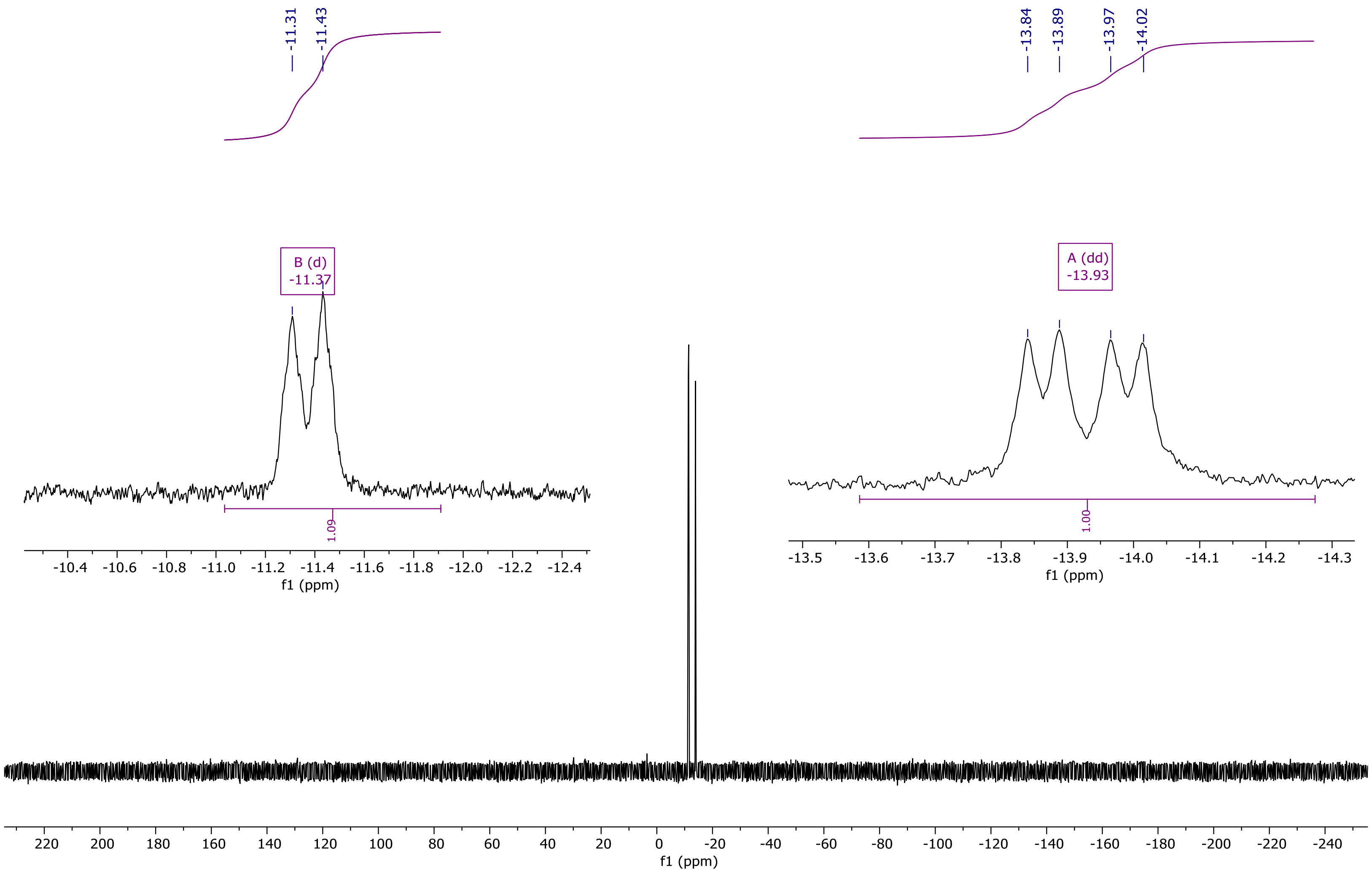

$^{31}P\{^1H\}$ NMR (inset are the ^{31}P NMR with ^1H couplings)

Guanosine Diphosphate-2''-deoxy-2''-fluoro-mannose (11)

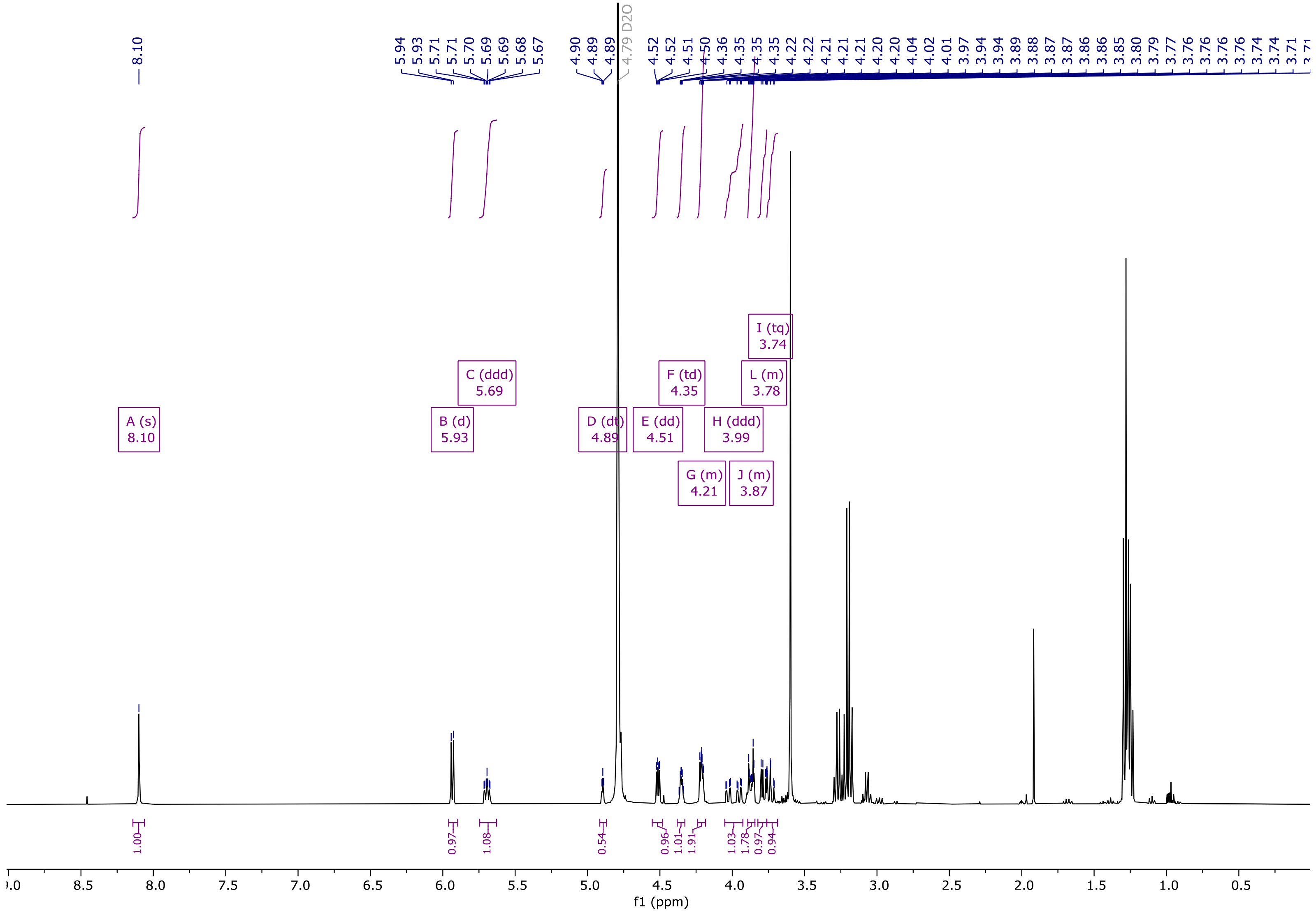

^{1}H NMR

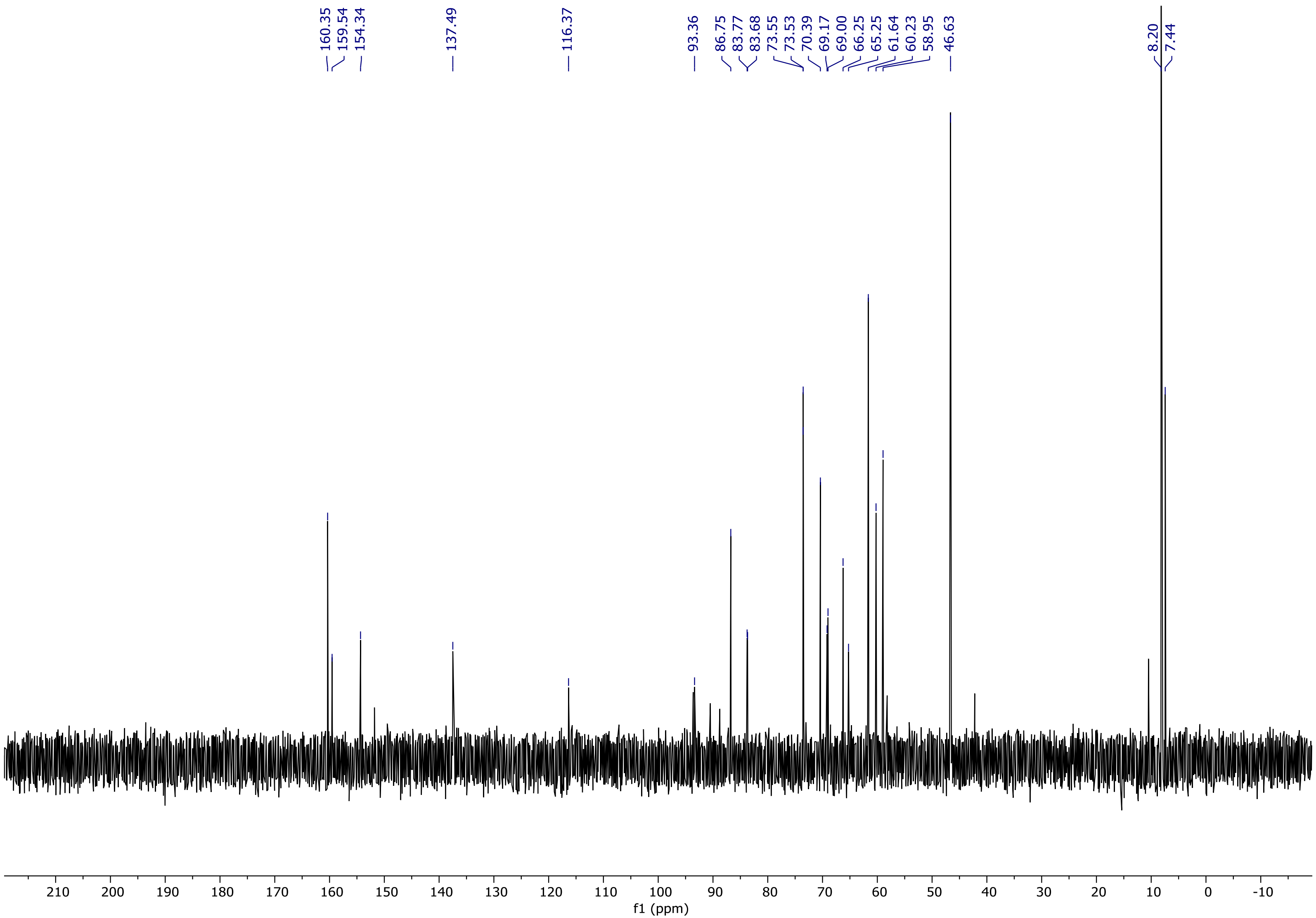
160.35
159.54
154.34
137.49
116.37
93.36
86.75
83.77
83.68
73.55
73.53
70.39
69.17
69.00
66.25
65.25
61.64
60.23
58.95
46.63
8.20
7.44
210 200 190 180 170 160 150 140 130 120 110 100 90 80 70 60 50 40 30 20 10 0 -10
f1 (ppm)

^{13}C NMR

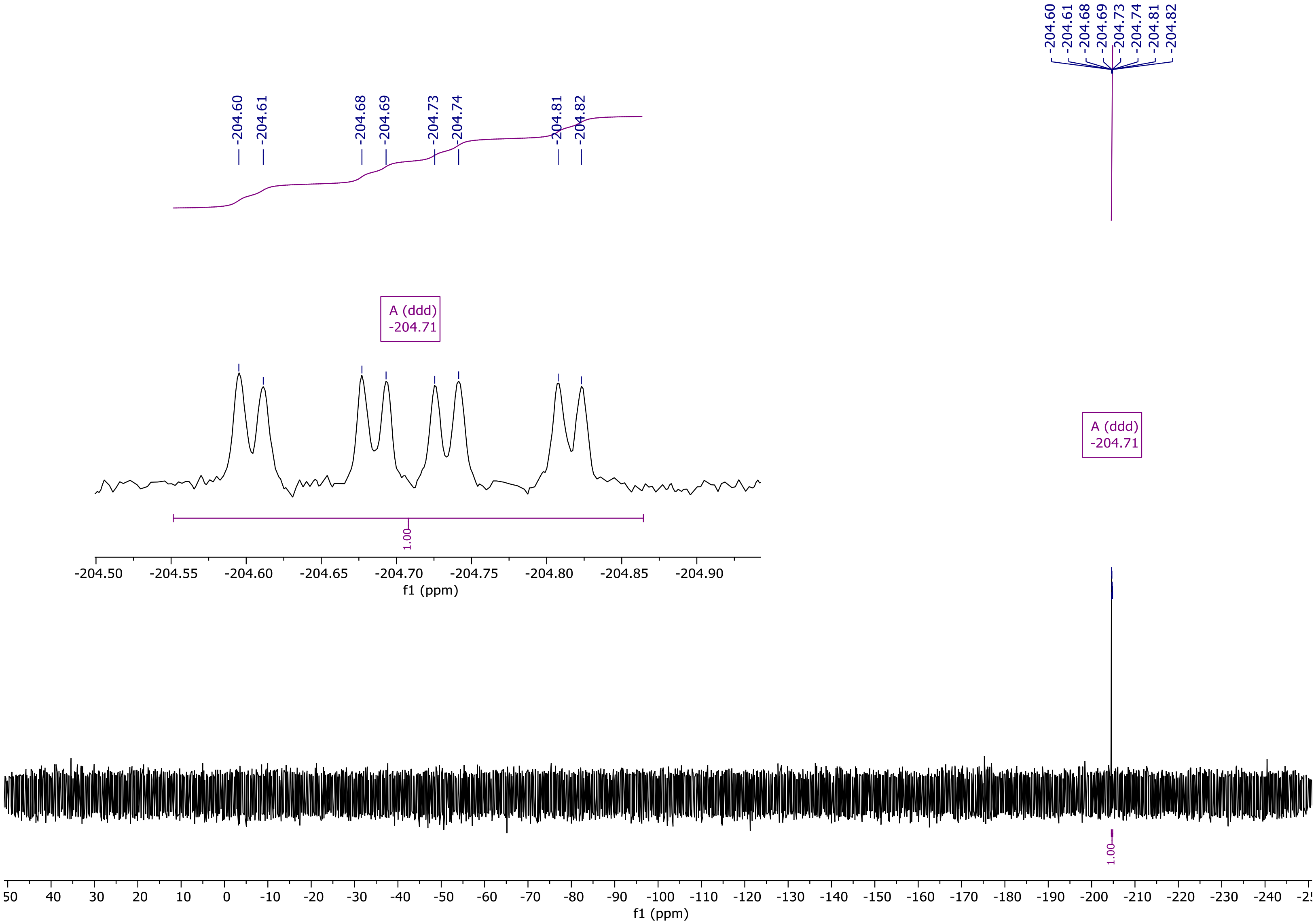

-204.60
-204.61
-204.68
-204.69
-204.73
-204.74
-204.81
-204.82
A (ddd)
-204.71
1.00
-204.50 -204.55 -204.60 -204.65 -204.70 -204.75 -204.80 -204.85 -204.90
f1 (ppm)
50 40 30 20 10 0 -10 -20 -30 -40 -50 -60 -70 -80 -90 -100 -110 -120 -130 -140 -150 -160 -170 -180 -190 -200 -210 -220 -230 -240
f1 (ppm)

^{19}F NMR

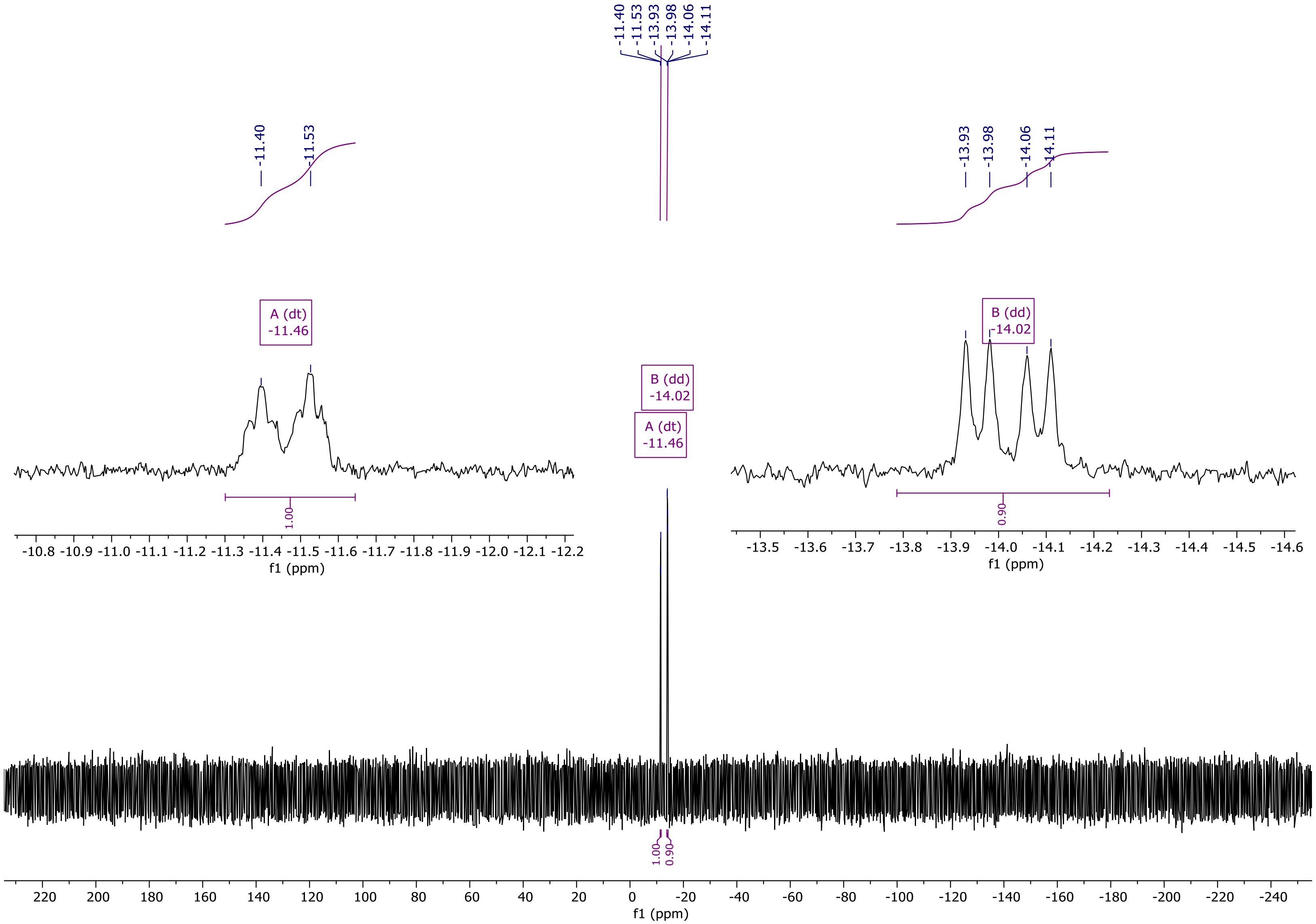

$^{31}P\{^{1}H\}$ NMR (inset are the ^{31}P NMR with ^{1}H couplings)

2’-deoxy-2’-fluoro-Guanosine Diphosphate mannose (13)

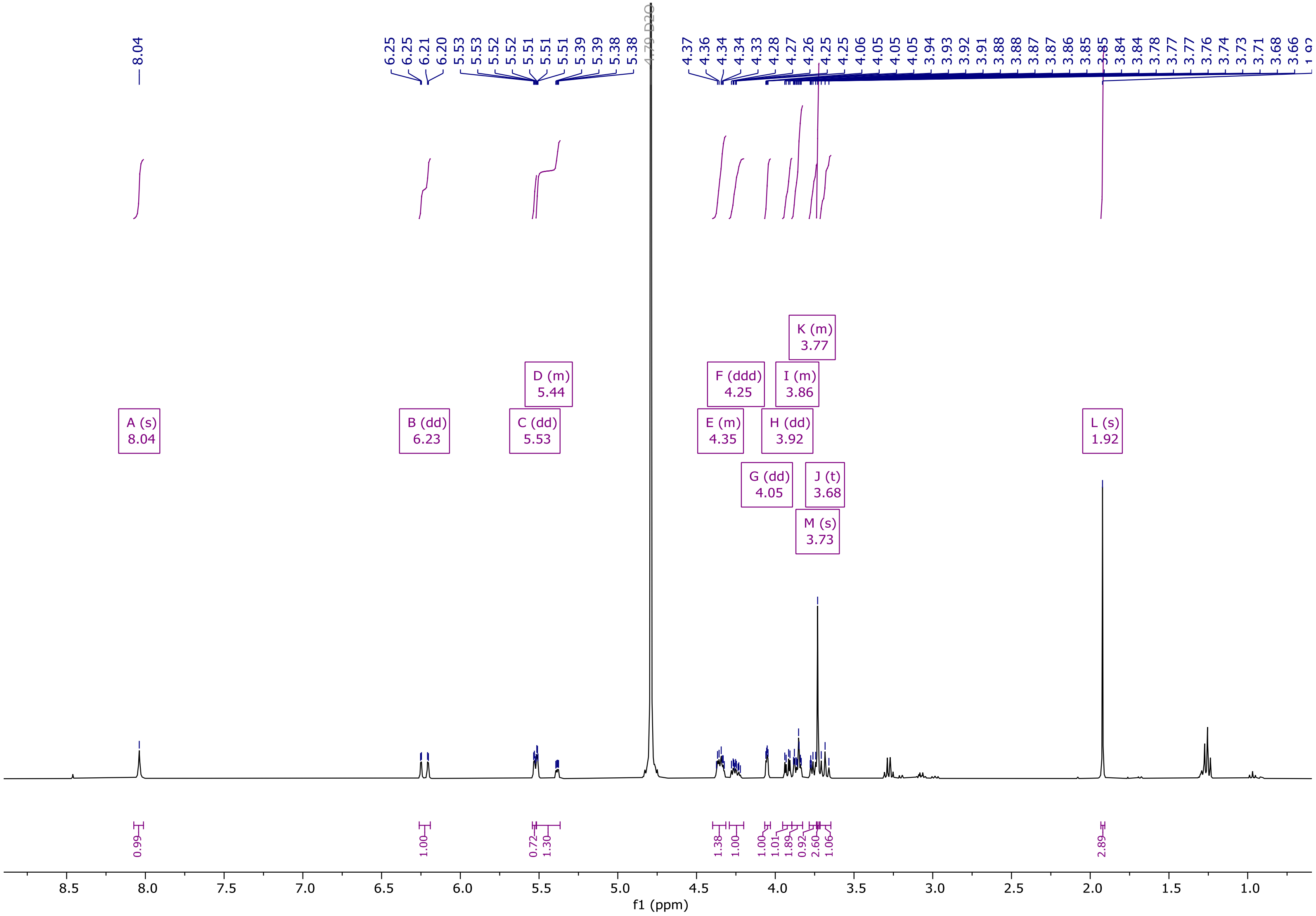

^{1}H NMR

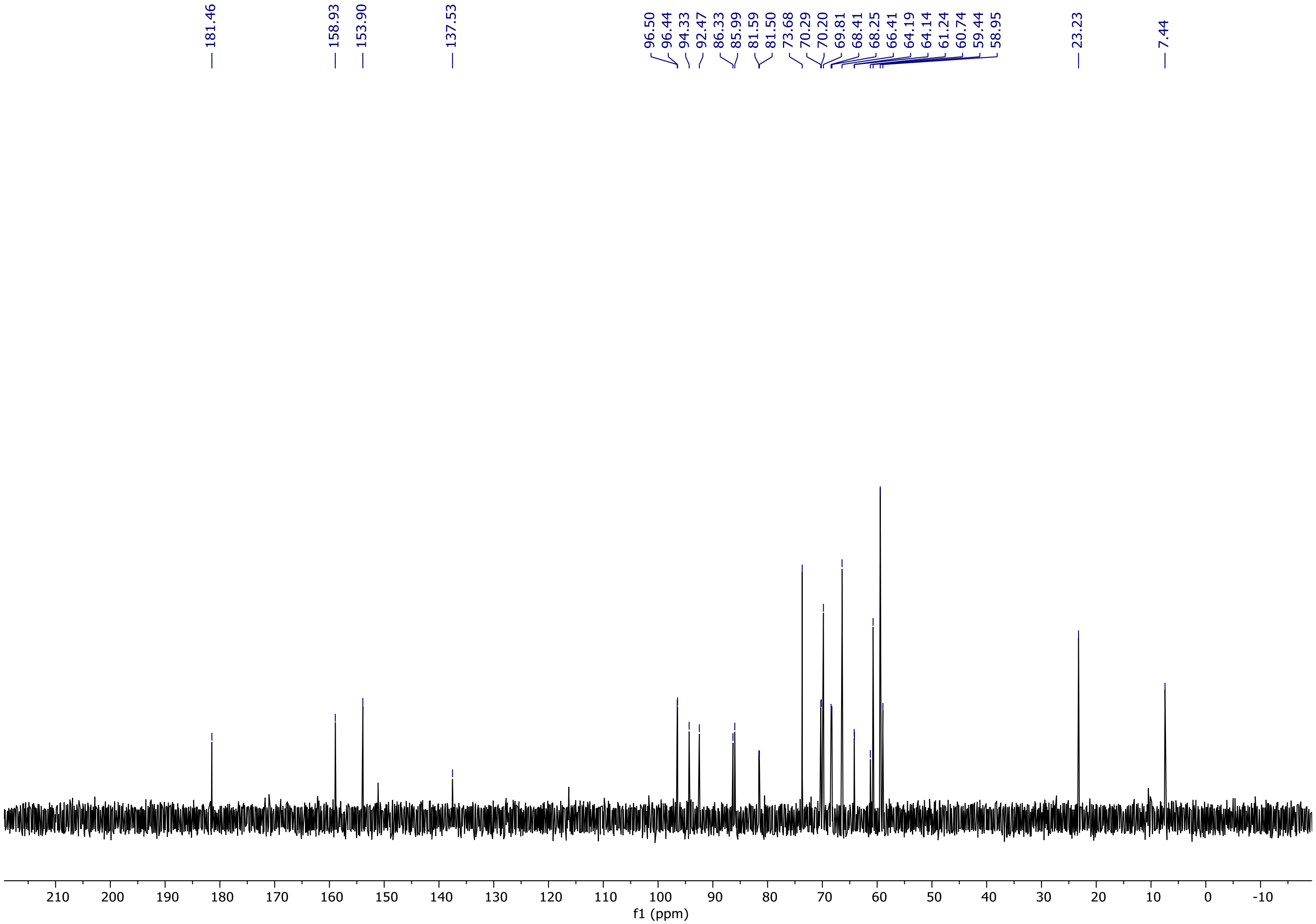

181.46
158.93
153.90
137.53
96.50
96.44
94.33
92.47
86.33
85.99
81.59
81.50
73.68
70.29
70.20
69.81
68.41
68.25
66.41
64.19
64.14
61.24
60.74
59.44
58.95
23.23
7.44
210 200 190 180 170 160 150 140 130 120 110 100 90 80 70 60 50 40 30 20 10 0 -10
f1 (ppm)

^{13}C NMR

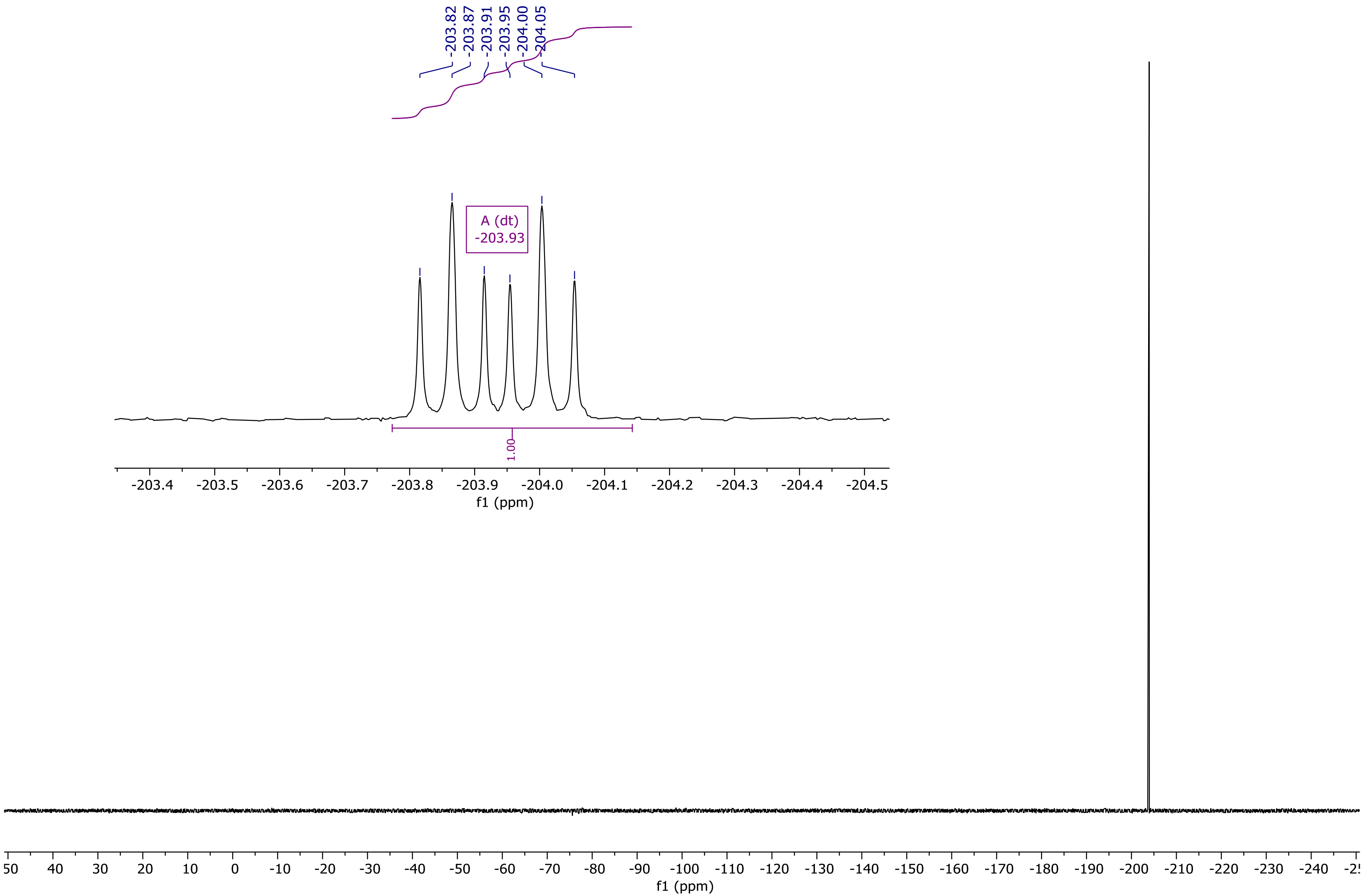
-203.82
-203.87
-203.91
-203.95
-204.00
-204.05
A (dt)
-203.93
1.00
-203.4 -203.5 -203.6 -203.7 -203.8 -203.9 -204.0 -204.1 -204.2 -204.3 -204.4 -204.5
f1 (ppm)
50 40 30 20 10 0 -10 -20 -30 -40 -50 -60 -70 -80 -90 -100 -110 -120 -130 -140 -150 -160 -170 -180 -190 -200 -210 -220 -230 -240
f1 (ppm)

^{19}F NMR

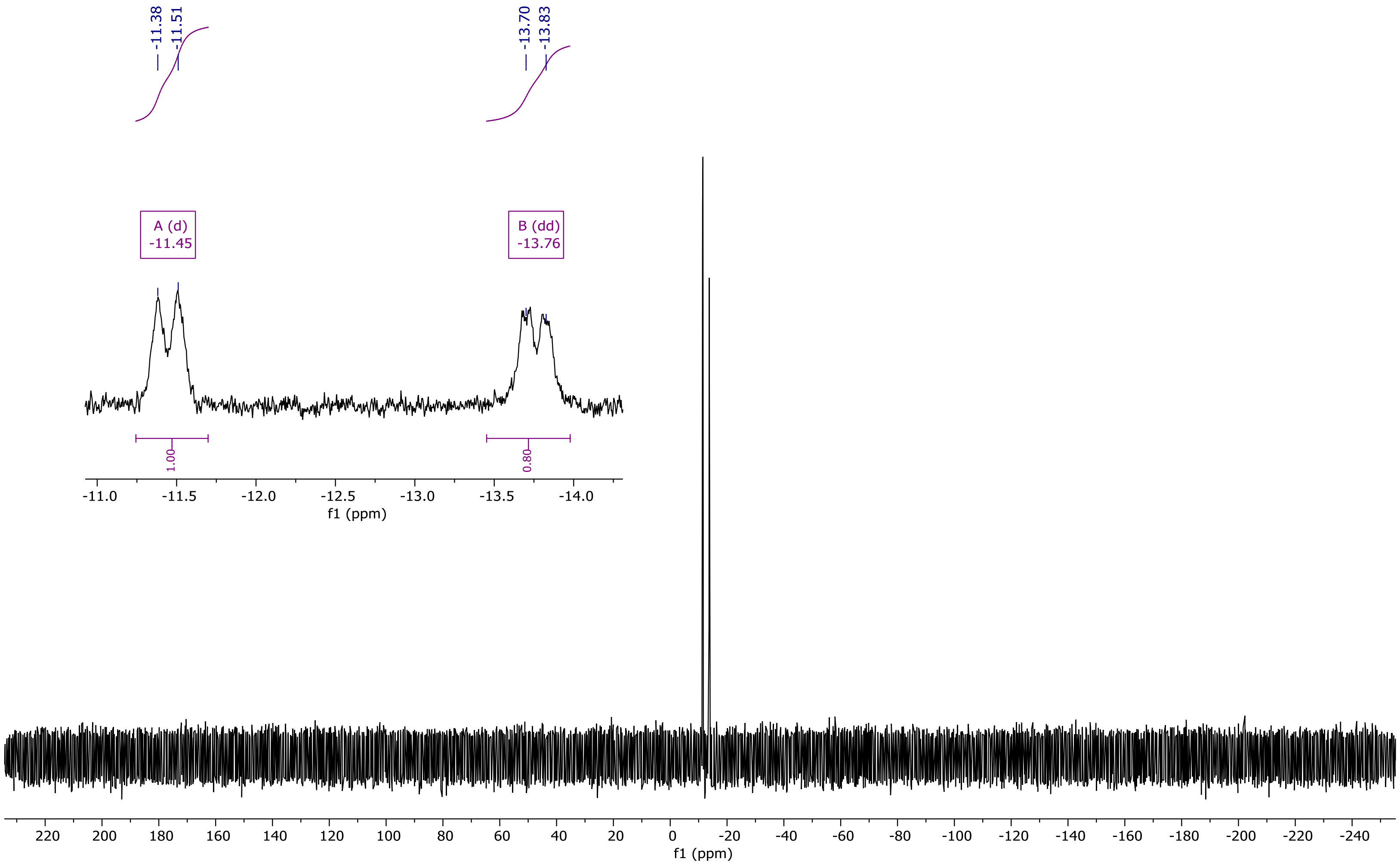

$^{31}P\{^{1}H\}$ NMR (inset are the ^{31}P NMR with ^{1}H couplings)

2’-deoxy-2’-fluoro- Guanosine Diphosphate-2’’-deoxy-2’’-fluoro-mannose (14)

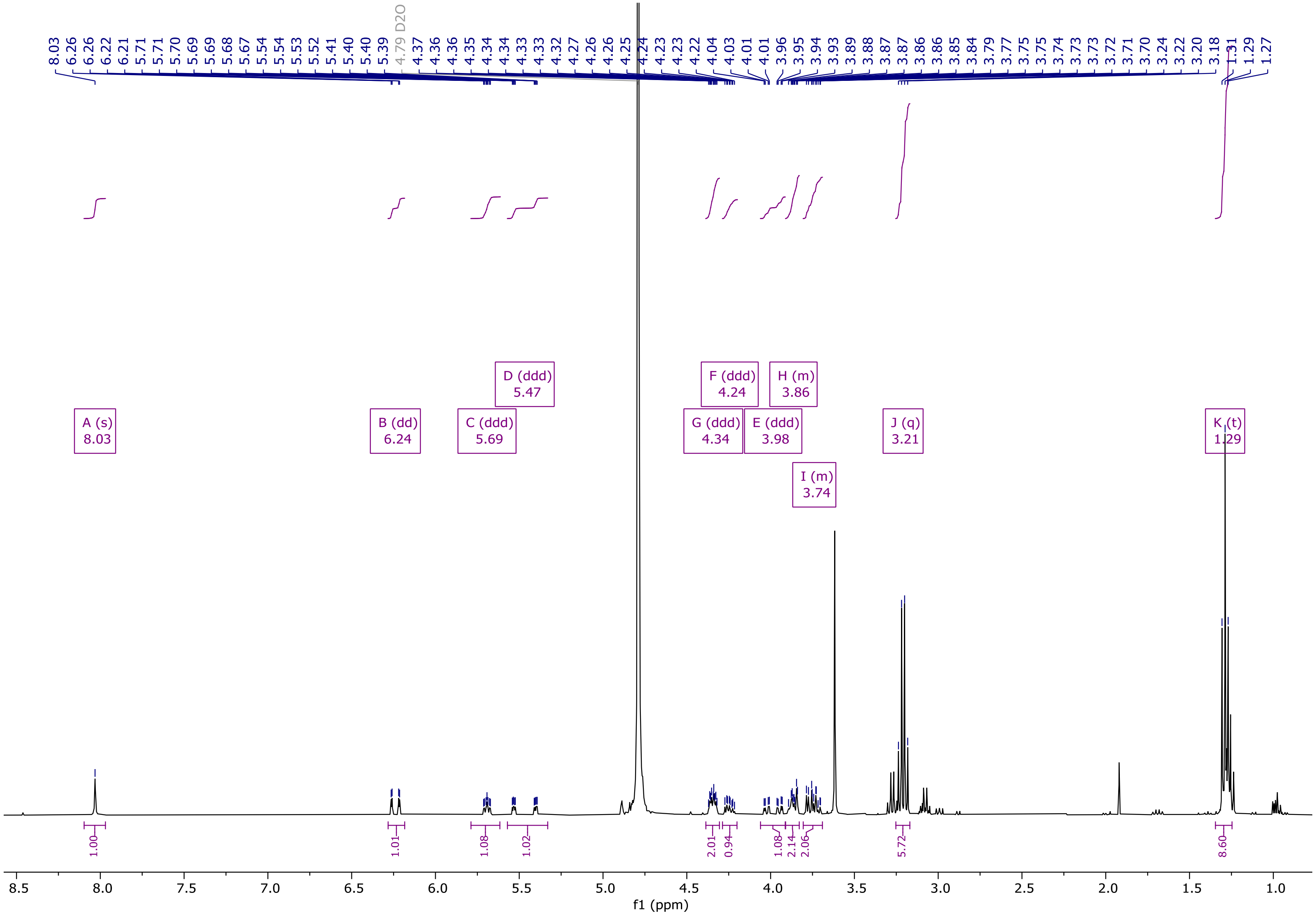

^{1}H NMR

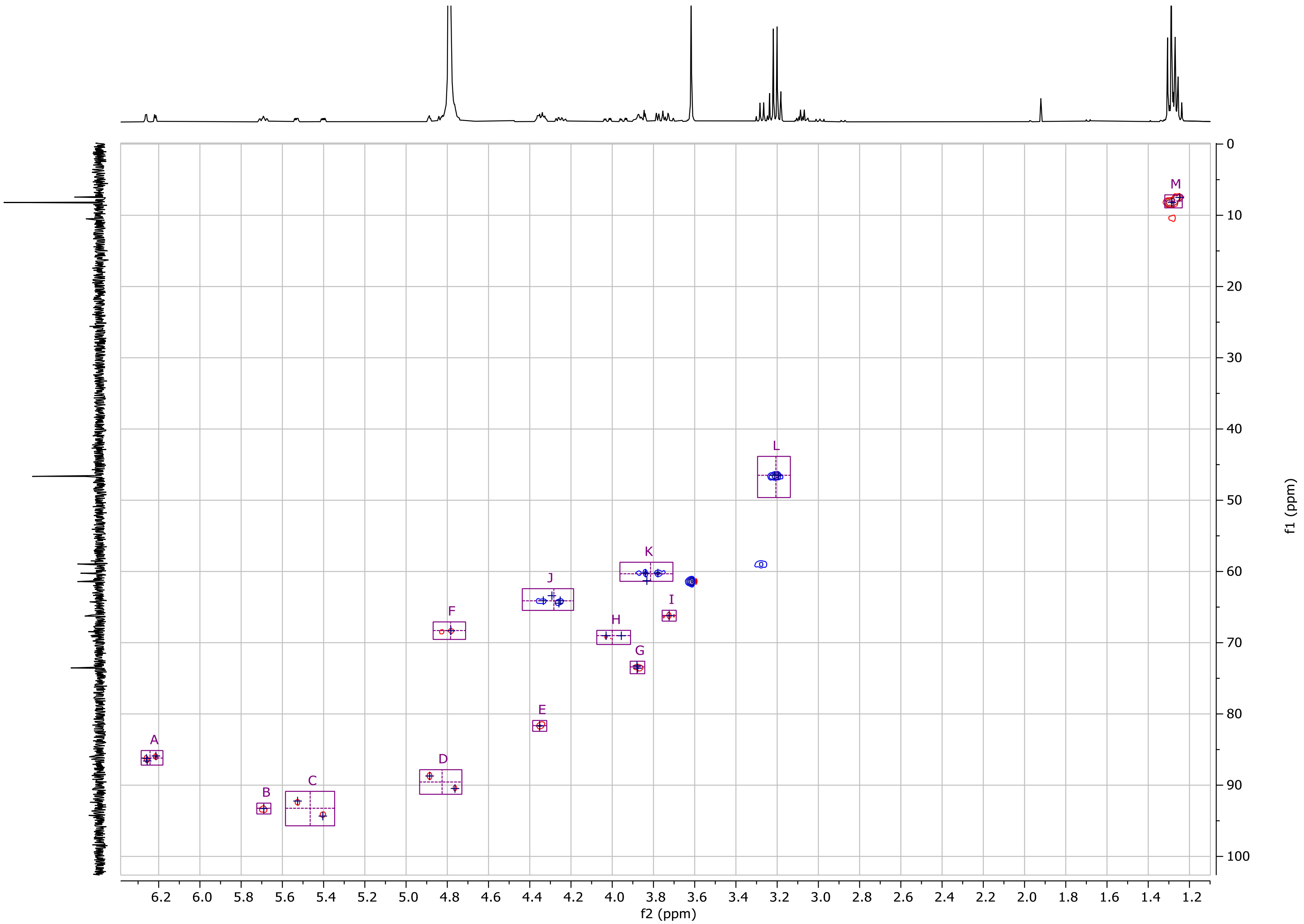

^{1}H-^{13}C HSQC NMR

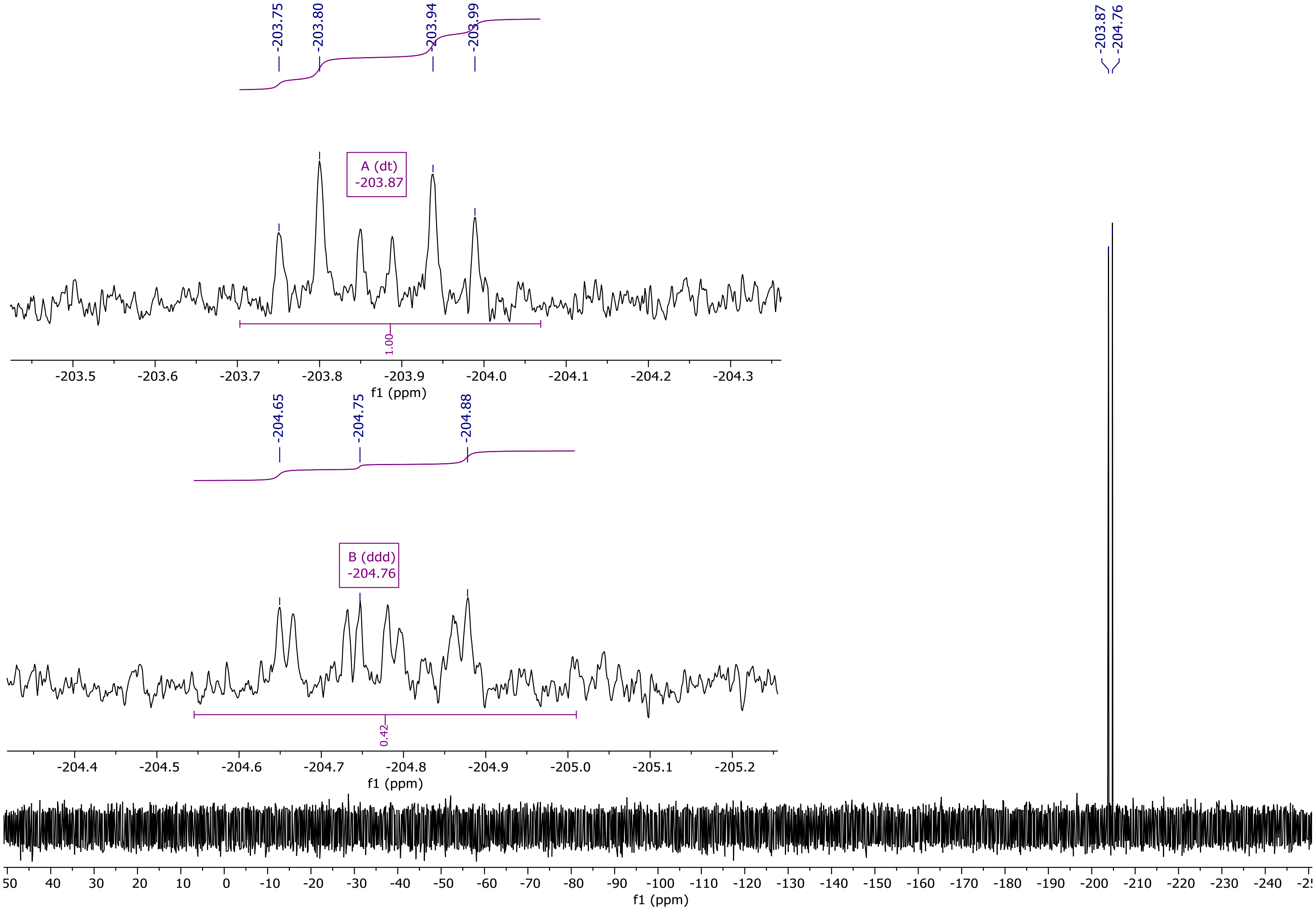

-203.75
-203.80
-203.94
-203.99
A (dt)
-203.87
1.00
-203.5 -203.6 -203.7 -203.8 -203.9 -204.0 -204.1 -204.2 -204.3
f1 (ppm)
-204.65
-204.75
-204.88
B (ddd)
-204.76
0.42
-204.4 -204.5 -204.6 -204.7 -204.8 -204.9 -205.0 -205.1 -205.2
f1 (ppm)
-203.87
-204.76
50 40 30 20 10 0 -10 -20 -30 -40 -50 -60 -70 -80 -90 -100 -110 -120 -130 -140 -150 -160 -170 -180 -190 -200 -210 -220 -230 -240
f1 (ppm)

^{19}F NMR

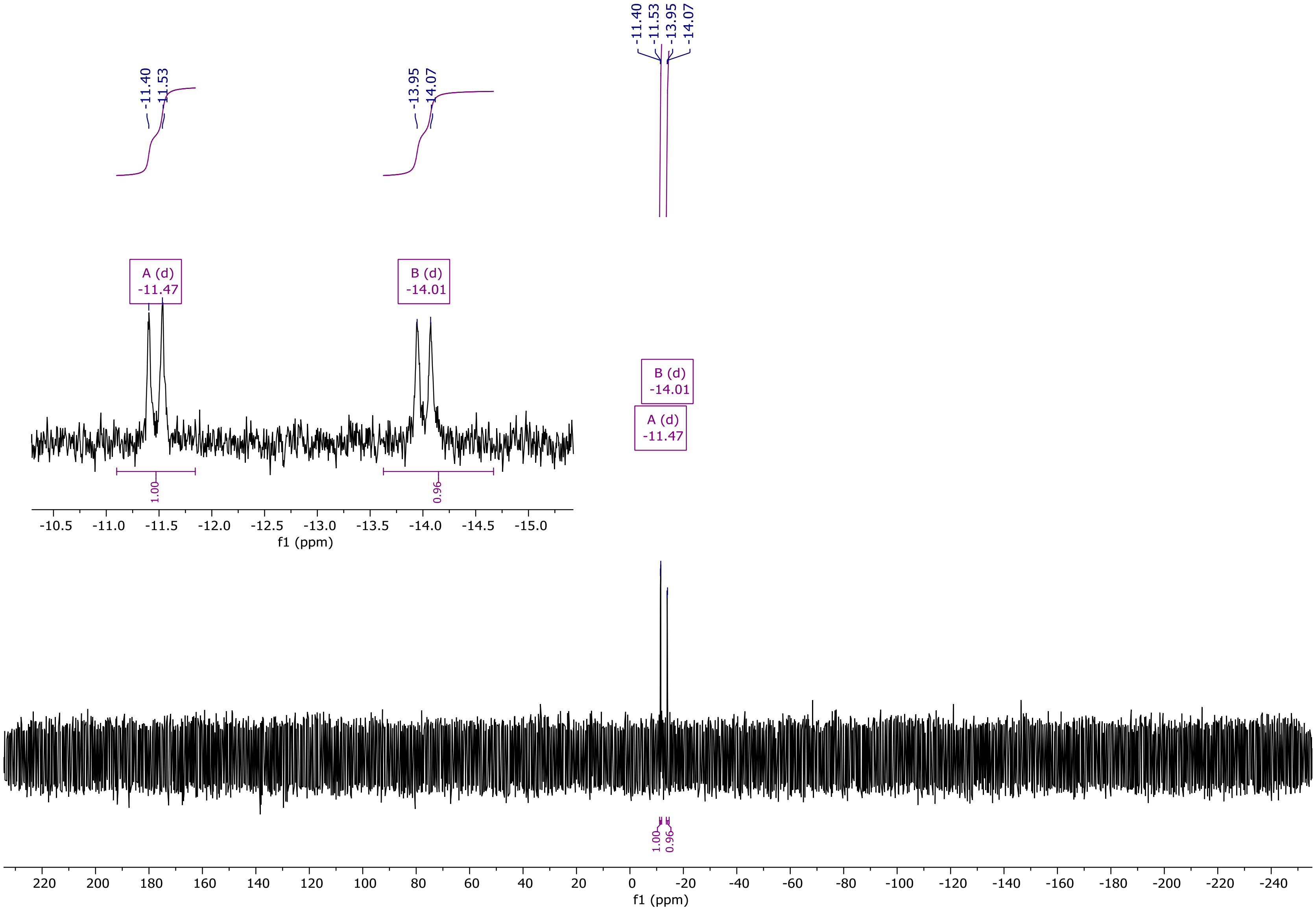

$^{31}P\{^{1}H\}$ NMR (inset are the ^{31}P NMR with ^{1}H couplings)
